# New Alkaloids from the Hydrothermal Vent-Derived Fungus *Aspergillus clavatus* C2WU and Their Mitochondrial Protective and Anti-Photoaging Effects

**DOI:** 10.3390/md24080268

**Published:** 2026-08-03

**Authors:** Jiayu Pan, Chengzeng Zhou, Jihua Wei, David Simeunovic, Weihua Yan, Qizhao Yin, Mengji Zou, Xiaodan Wu, Zhe Feng, Minjie Zhang, Hu Huang, Bin Wu

**Affiliations:** 1 Ocean College, Zhejiang University, Zhoushan 316000, China12434004@zju.edu.cn (Q.Y.);; 2 Proya Cosmetics Co., Ltd., Hangzhou 310012, China; 3 Fujian Provincial Key Laboratory of Screening for Novel Microbial Products, Fujian Institute of Microbiology, Fuzhou 350007, China; 4 Quzhou Institute for Food and Drug Control, Quzhou 324000, China; 5 Analysis Center of Agrobiology and Environment Sciences, Zhejiang University, Hangzhou 310058, China

**Keywords:** fungus, *Aspergillus* sp., secondary metabolites, structural identification, mitochondrial function

## Abstract

Four new compounds (**1**–**4**), including two new quinazoline-containing indole alkaloids, tryptoquivaline Z_1_ (**1**) and clavutoine V (**2**); a new cytochalasan alkaloid, cytochalasin Z_29_ (**3**); and methyl (*S*)-2-(2,5-dihydroxyphenyl)-2-methoxyacetate (**4**), along with one known compound (**5**), were isolated from culture extracts of the hydrothermal vent crab-derived fungus *Aspergillus clavatus* C2WU. The structures of the new compounds, including their absolute configurations, were determined by NMR and MS spectroscopic data analyses and comparison between the calculated and experimental ECD spectra. In vitro, compound **2** (clavutoine V) preserves mitochondrial function by reducing the level of mitochondrial membrane potential (MMP) and increasing mitochondrial ATP production. Furthermore, compound **2** might regulate lipid metabolism by reducing ROS. Complementary molecular dynamics simulations support a cardiolipin-associated membrane-modulation mechanism, suggesting that compound **2** may melt rigid lipid domains to restore membrane electrostatic homeostasis. Compounds **1** (tryptoquivaline Z_1_), **2** (clavutoine V), and **5** (arthriniumnin A) effectively attenuated UVB-induced mitochondrial dysfunction and ROS overproduction in skin cells, demonstrating their anti-photoaging potential. Additionally, compound **4** (methyl (*S*)-2-(2,5-dihydroxyphenyl)-2-methoxyacetate) displayed strong ability to scavenge free radicals with an IC_50_ value of 34.3 μM. It not only reduced UVB-induced ROS production in HaCaT cells but also attenuated glucose-induced AGE formation in HDF cells, further confirming its antioxidant capacity. These findings highlight the potential of hydrothermal vent-derived fungi as a source of bioactive leads for dermatological applications, anti-aging interventions, and mitochondrial medicine.

## 1. Introduction

Marine-derived fungi, thriving under extreme and competitive conditions such as fluctuating pH, temperature, pressure, oxygen, light, and salinity, have emerged as a highly promising resource for drug discovery due to their prolific production of structurally novel and biologically active secondary metabolites [1,2]. The exceptional physicochemical parameters of their habitats drive evolutionary adaptations that result in unique biosynthetic pathways and enhanced chemical diversity [3].

Of particular interest are fungi inhabiting deep-sea hydrothermal vents—among the most extreme and dynamic environments on Earth. All of these characteristics make vent areas an extreme environment that is challenging for animals to thrive. The hydrothermal vent crab can adapt to the environment. Crab shells provide a physical substrate for microbial colonization but also a unique source of activation factors and nutrients [4]. Studies have shown that the epiphytic fungi associated with them also possess special metabolic products [5], which often show pharmaceutically relevant bioactivities and may be candidates for the development of new drugs [6]. A variety of new natural products such as cyclopeptides, quinazoline derivatives, and oxepin-containing have been isolated from hydrothermal vent crab-associated fungi, which possess biological activities like antibacterial and anti-cancer properties [7,8].

To date, only a limited number of marine fungi isolated from crabs have been subjected to chemical and pharmacological investigation. In order to find drug candidates from the microorganisms which live at extreme and toxic habitats, natural product-producing fungi from the hydrothermal vent crab were cultured in our laboratory. This study aimed to isolate and identify novel secondary metabolites from the hydrothermal vent crab-associated fungus *Aspergillus clavatus* C2WU, and to evaluate their hepatoprotective effects in an in vitro model of lipotoxicity. In addition, the antifungal, antibacterial, and antioxidant activities of the isolated compounds were also investigated.

## 2. Results

**Figure 1:**
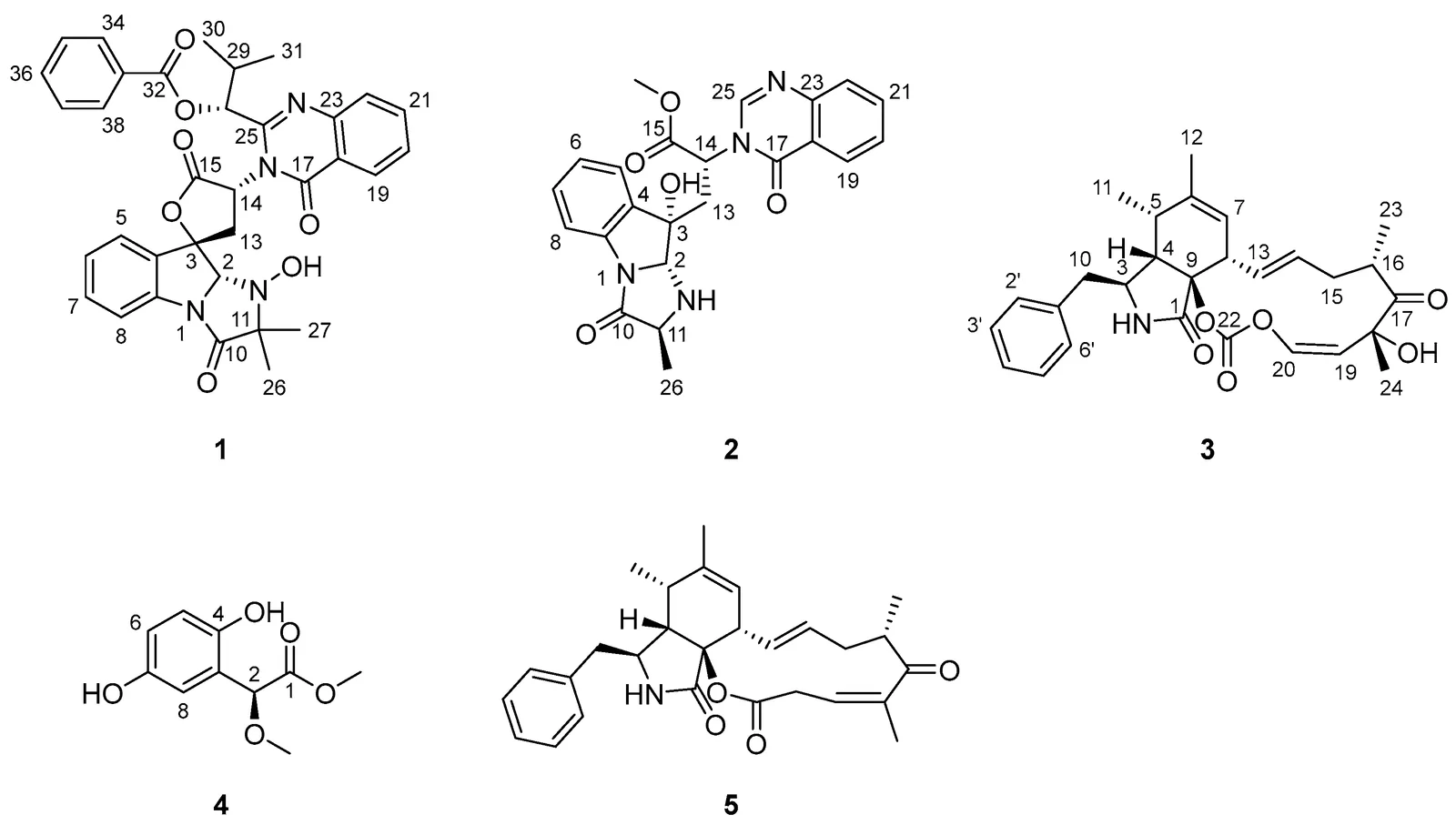
Compounds **1**–**5** from *A. clavatus* C2WU.

Compound numbering and nomenclature. The new compounds are designated as compounds **1**–**4**, and the known compound as **5** (arthriniumnin A). Compound **1**: tryptoquivaline Z_1_; compound **2**: clavutoine V; compound **3**: cytochalasin Z_29_; compound **4**: methyl (*S*)-2-(2,5-dihydroxyphenyl)-2-methoxyacetate. A summary of all compounds is provided in Table 1 and Figure 1.

### 2.1. Structure Elucidation

**Figure 2:**
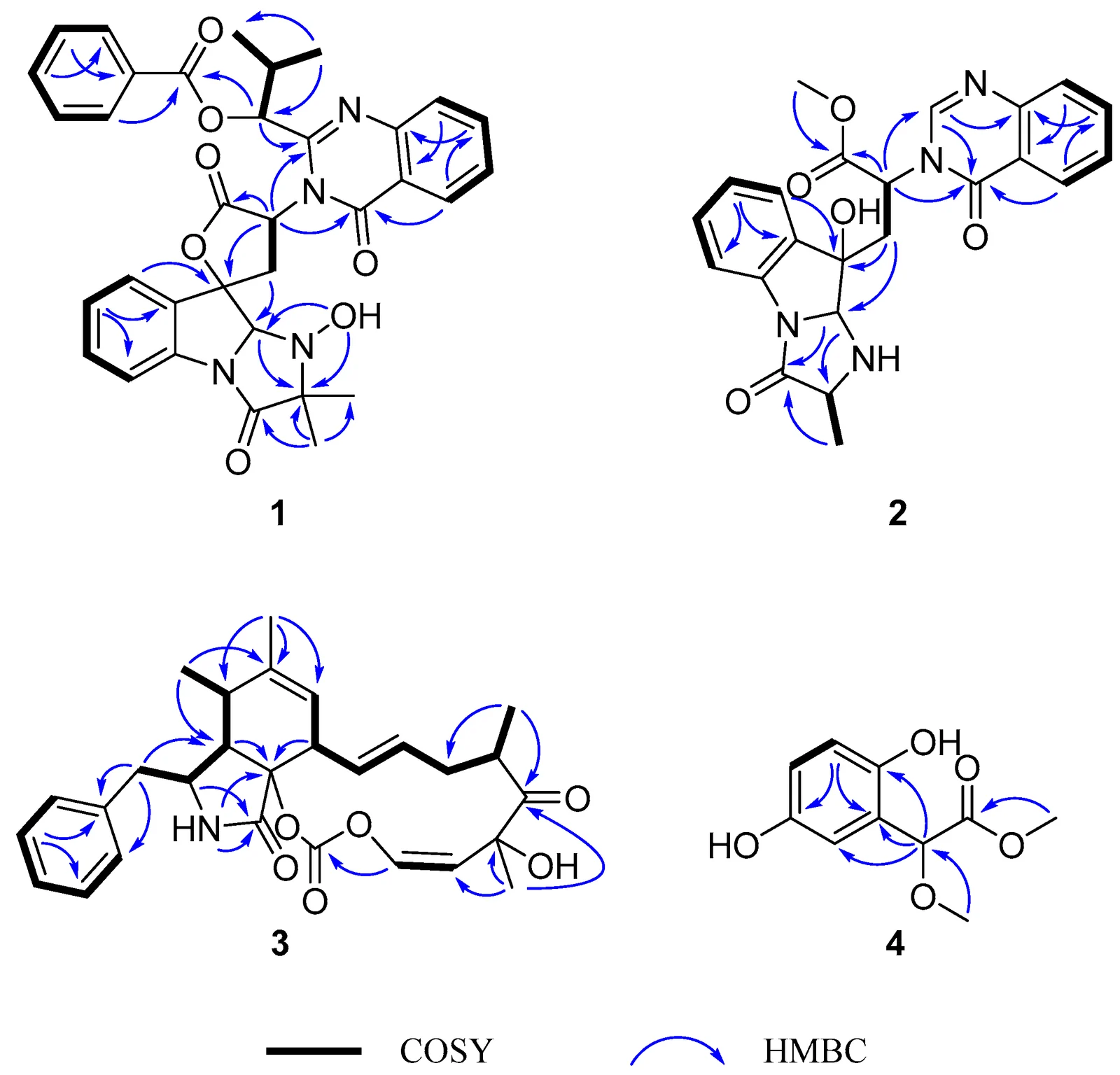
Key ^1^H−^1^H COSY and HMBC correlations of **1**−**4**.

**Figure 3:**
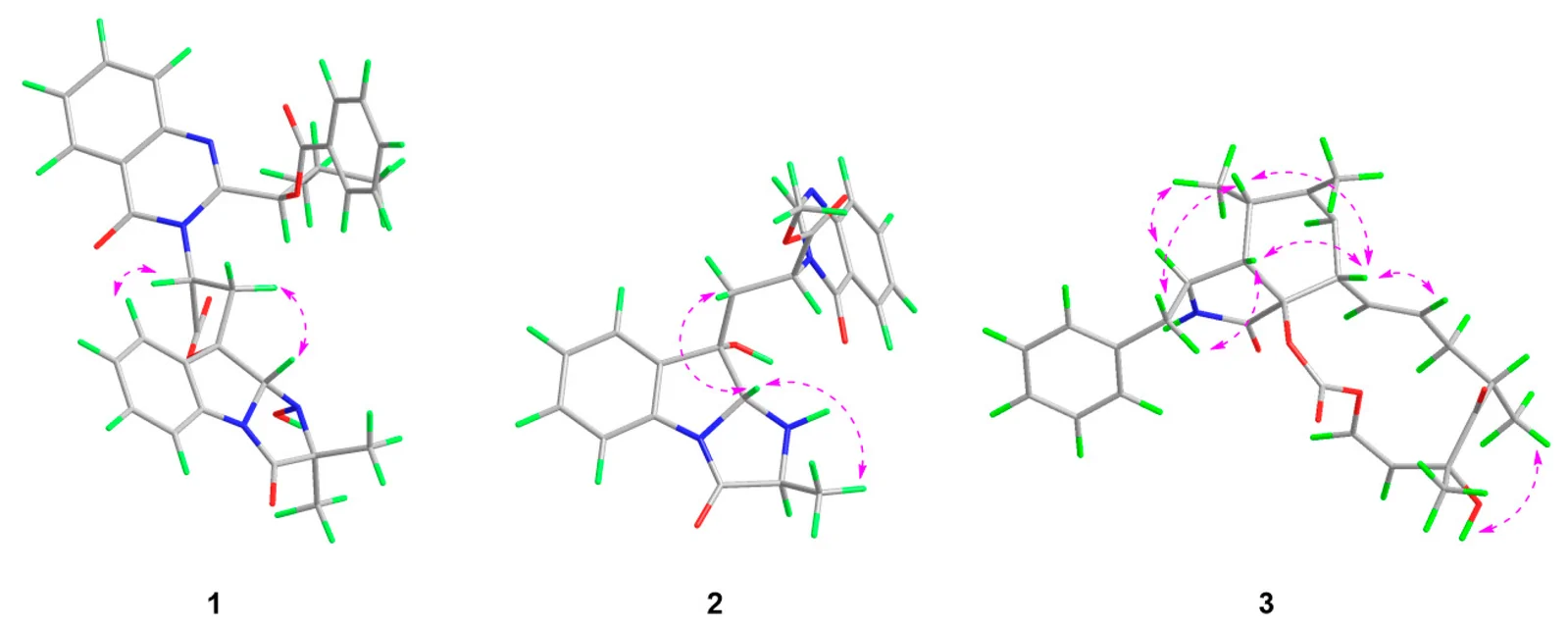
Key NOESY correlations of **1**−**3**.

**Figure 4:**
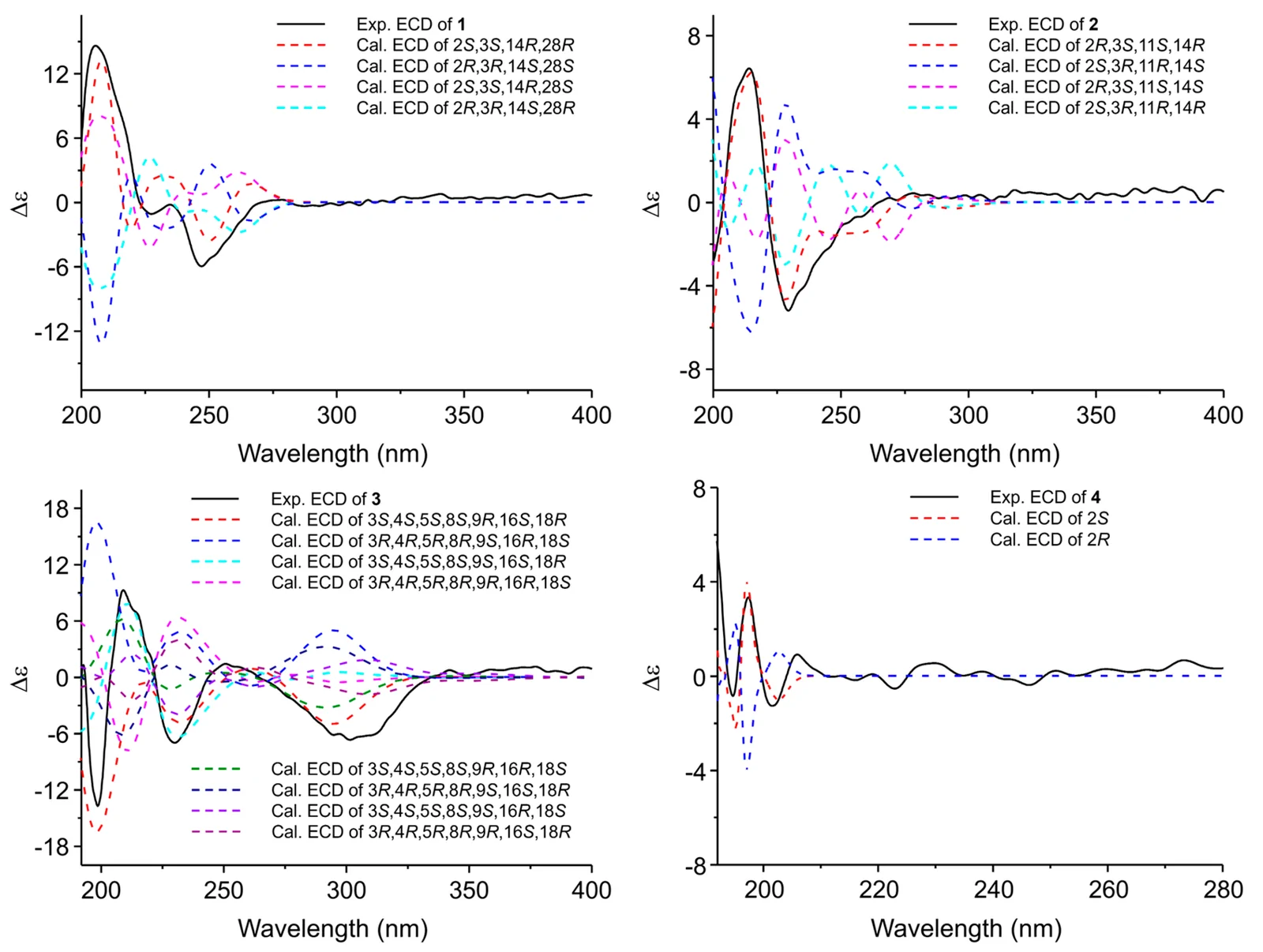
Experimental and calculated ECD spectra of **1**–**4**.

Compound **1** was assigned the molecular formula C_34_H_32_N_4_O_7_ based on the molecular ion peak at *m/z* 609.2345 [M + H]^+^ in the HRESIMS. The ^13^C NMR and HSQC spectra (Table 2, Figures S2 and S3) revealed a total of 34 carbons, including four methyl groups (*δ*_C_ 17.5, 18.3, 18.8, 23.4), 18 aromatic unsaturated carbons (*δ*_C_ 115.3, 120.2, 125.8, 126.5, 126.9, 127.9, 128.7, 129.1, 129.6, 129.6, 129.9, 130.4, 132.0, 134.5, 135.1, 136.3, 137.6, 146.7), four methines (*δ*_C_ 32.9, 55.3, 76.8, 86.7), and four amide or ester carbonyls (*δ*_C_ 161.0, 166.4, 170.7, 171.1). The NMR data of **1** was similar to those previously reported for tryptoquivaline [9]. Therefore, it was initially speculated that **1** was a quinazoline-containing indole alkaloid. The main difference was the absence of signals for monosubstituted benzene ring in tryptoquivaline. Based on the COSY correlations of H_3_-30 (*δ*_H_ 1.24) and H_3_-31 (*δ*_H_ 1.03) coupled with a methine H-29 (*δ*_H_ 2.62) and HMBC correlations of H-34 (*δ*_H_ 7.98) to C-32 (*δ*_C_ 166.4), H-28 (*δ*_H_ 6.11) to C-25 (*δ*_C_ 155.3), C-32, H-30 to C-29 (*δ*_C_ 32.9) and H-31 to C-28 (*δ*_C_ 76.8) (Figure 2, Figures S4 and S5), it can be inferred that the 2-methylpropyl benzoate moiety was attached to the quinazoline core via C-25. The relative configuration of **1** was first established by NOESY analysis (Figure 3 and Figure S6). Key correlations between H-2 (*δ*_H_ 5.24) and H_b_-13 (*δ*_H_ 2.99), and between H-5 (*δ*_H_ 8.04) and H-14 (*δ*_H_ 6.16), defined the spatial proximity of these protons. These correlations rule out alternative configurations at C-2, C-3, and C-14, because any inversion at these centres would require different NOE contacts. The relative configuration was assigned as either 2S*, 3S*, 14R*, 28R* or 2S*, 3S*, 14R*, 28S*. The absolute configuration was then confirmed by ECD calculation (Figure 4), which showed excellent agreement with the 2*S*,3*S*,14*R*,28*R*. The chemical structure of compound **1** was established as a new quinazoline-containing indole alkaloid named tryptoquivaline Z_1_ (**1**).

Compound **2** was isolated as white powder. Its molecular formula was determined to be C_23_H_22_N_4_O_5_ based on the molecular ion peak at *m*/*z* 435.1663 [M + H]^+^ in the HRESIMS spectrum, suggesting 15 unsaturation degrees. The ^1^H NMR spectrum of **2** (Table 2) showed resonances for 21 protons, including an exchangeable proton at *δ*_H_ 5.46. The ^13^C NMR, HMBC, and HSQC spectra confirmed the presence of 23 carbons (Table 2). The ^13^C NMR (Table 2) signals were assigned to two methyl groups (*δ*_C_ 18.3, 53.3), one methylene (*δ*_C_ 36.1), 12 aromatic unsaturated carbons (*δ*_C_ 115.6, 122.1, 124.9, 125.3, 126.7, 127.7, 127.7, 130.1, 135.2, 138.0, 139.1, 148.3), 3 methines (*δ*_C_ 57.9, 61.0, 83.0), and three amide or ester carbonyls (*δ*_C_ 160.4, 170.0, 175.5). All the carbon and the corresponding proton signals were assigned by extensive analysis of the HSQC spectrum (Figure S10). The ^1^H NMR and ^13^C NMR data of **2** were similar to **1**. They were indole alkaloids of the same type containing quinazoline. The multiplicity of the aromatic proton signals in the ^1^H NMR and ^13^C NMR revealed the presence of two benzene rings. The HMBC correlations from H-25 (*δ*_H_ 8.41) to C-14 (*δ*_C_ 57.9), C-17 (*δ*_C_ 160.4), as well as from H-19 (*δ*_H_ 8.13) to C-17, permitted the identification of *N*-substituted quinazolin 4-one unit (Figure 2 and Figure S12). Analysis of the HMBC spectrum indicated that another 1,2-disubstituted benzene ring belonged to the indoline moiety, which was substantiated by the correlations from H-2 (*δ*_H_ 5.32) and H-5 (*δ*_H_ 7.32) to C-3 (*δ*_c_ 76.5). An exchangeable proton at *δ*_H_ 5.46 (s) was assigned to the 3-OH, which showed HMBC correlations to C-2 (*δ*_c_ 83.0), C-3 (*δ*_c_ 76.5), and C-13 (*δ*_c_ 36.1). Cross-peaks in the HMBC spectrum from H-14 (*δ*_H_ 5.19) and 15-OCH_3_ (*δ*_c_ 53.3) to C-15 (*δ*_c_ 170.0) established partial fragment. All of above fragments are connected via C-14 and N-16, as well as C-3 and C-13 (*δ*_c_ 36.1), as evidenced by the HMBC correlations. And **2** was quite similar to the clavutoine N that previously reported [10], except that there was one less methyl group compared to clavutoine N located at C-11. The relative configuration of **2** was established by NOESY correlations (Figure 3 and Figure S13). The correlations between H-2 (*δ*_H_ 5.32) and H_3_-26 (*δ*_H_ 1.35), and between H-2 and H_b_-13 (*δ*_H_ 2.60), placed these protons on the same face. The relative configuration was defined as 2R*, 3S*, 11S*, 14R* or 2R*, 3S*, 11S*, 14S*. The ECD spectrum of the proposed 2*R*, 3*S*, 11*S*, 14*R* matched the experimental curve (Figure 4), confirming the absolute configuration. Based on the above analyses, the chemical structure of compound **2** was established as a new indole alkaloid named clavutoine V (**2**).

Compound **3** was a white powder. The molecular formula was established as C_28_H_33_NO_6_ based on the molecular ion peak at *m*/*z* 480.2379 [M + H]^+^ in the HRESIMS spectrum, indicating 13 unsaturation degrees. The ^1^H-NMR spectrum (Table 3) showed the presence of an amide NH (*δ*_H_ 8.13), two methyl doublets (*δ*_H_ 0.60, and 0.94), and two methyl singlets (*δ*_H_ 1.28, and 1.62). Five olefinic H-atoms (*δ*_H_ 5.17, 5.29, 5.35, 5.53, and 6.45) and a phenyl ring (*δ*_H_ 7.14–7.26) were also observed. The 13C-NMR and HSQC spectra of **3** (Table 3, Figures S16 and S17) revealed the presence of four methyl (*δ*_C_ 13.7, 19.8, 20.2, 28.0), two methylene (*δ*_C_ 37.5, 43.2), and six aromatic unsaturated carbons (*δ*_C_ 127.0, 128.9, 128.9, 130.1, 130.1, 138.4), as well as seven quaternary C-atoms, including a carbonate CO_3_ (*δ*_C_ 150.1), an amide CO (*δ*_C_ 170.1), and a ketone CO group (*δ*_C_ 217.6). Interpretation of the 1D-NMR spectra led to the conclusion that, with respect to that of phenochalasin A [11], **3** had the same 10-phenylperhydroisoindol-1-one skeleton, except for the absence of the 4′-OH. The proton coupling constant was 12.4 Hz between H-19 (*δ*_H_ 5.35) and H-20 (*δ*_H_ 6.45), indicating that the olefin has the *cis* configuration. According to the NOESY correlations between 18-OH (*δ*_H_ 5.83) with H_3_-23 (*δ*_H_ 0.94), H-8 (*δ*_H_ 2.80) with H-4 (*δ*_H_ 2.67), H-5 (*δ*_H_ 2.42), H-14 (*δ*_H_ 5.29), H-3 (*δ*_H_ 2.94) with H_3_-11 (*δ*_H_ 0.6), H_2_-10 (*δ*_H_ 2.84) with H-5, H-4 (Figure 3 and Figure S20), the relative configuration of **3** was assigned as one of four possibilities: 3S*, 4S*, 5S*, 8S*, 9R*, 16S*, 18R*; 3S*, 4S*, 5S*, 8S*, 9S*, 16S*, 18R*; 3S*, 4S*, 5S*, 8S*, 9R*, 16R*, 18S*; or 3S*, 4S*, 5S*, 8S*, 9S*, 16R*, 18S*. The absolute configuration of **3** was assigned as 3*S*,4*S*,5*S*,8*S*,9*R*,16*S*,18*R*, which is the same as that of cytochalasin E [12], as supported by ECD calculations (Figure 4). Taken together, the structure of compound **3** was elucidated as a new cytochalasan alkaloid named cytochalasin Z_29_ (**3**) (Figure 1).

The molecular formula of compound **4** was determined to be C_10_H_12_O_5_ based on the molecular ion peak at *m*/*z* 235.0574 [M + Na]^+^ in the HRESIMS spectrum, suggesting five unsaturation degrees. The ^1^H NMR spectrum of **4** (Table 4) showed resonances for 10 protons. The ^13^C NMR and HSQC spectra confirmed the presence of 10 carbons (Table 4, Figures S23 and S24). The ^1^H NMR (Table 4) signals were assigned to two methoxy groups (*δ*_C_ 52.2, 57.3), one methine (*δ*_C_ 76.1), and 1,2,4-trisubstituted benzene ring (*δ*_C_ 114.5, 116.7, 116.7, 123.7, 148.1, 150.3). The linkage between C-1, C-2 and C-3 (*δ*_C_ 123.7) was indicated by HMBC coupling from H-2 (*δ*_H_ 5.01) to C-1, C-3, C-4 (*δ*_C_ 148.1), and C-8 (*δ*_C_ 114.5). Analyses of 1-OCH_3_(*δ*_H_ 3.57) to C-1 (*δ*_C_ 171.7) and 2-OCH_3_ (*δ*_H_ 3.22) to C-2 (*δ*_C_ 76.1) in HMBC correlation revealed the connection position of the methoxy groups at C-1 and C-2 (Figure S26). Based on the splitting patterns and coupling constants of H-5, H-6 and H-8 (d, *δ*_H_ 6.64, *J* = 8.6 Hz; dd, *δ*_H_ 6.52, *J* = 8.6 Hz, 2.3 Hz; d, *δ*_H_ 6.55, *J* = 2.3 Hz), along with ^13^C NMR (Table 4) signals of C-4 (*δ*_C_ 148.1) and C-7 (*δ*_C_ 150.3) in the ^13^C NMR, the hydroxyl groups at the C-4 and C-7 can be determined. The absolute configuration of **4**, named methyl (*S*)-2-(2,5-dihydroxyphenyl)-2-methoxyacetate (**4**), was assigned as 2*S* based on the calculated ECD spectrum of (*S*)-**4** was in agreement with the experimental one (Figure 4).

Spectroscopic comparison with previously reported data enabled the identification of the known compound arthriniumnin A (**5**) [13].

**Scheme 1:**
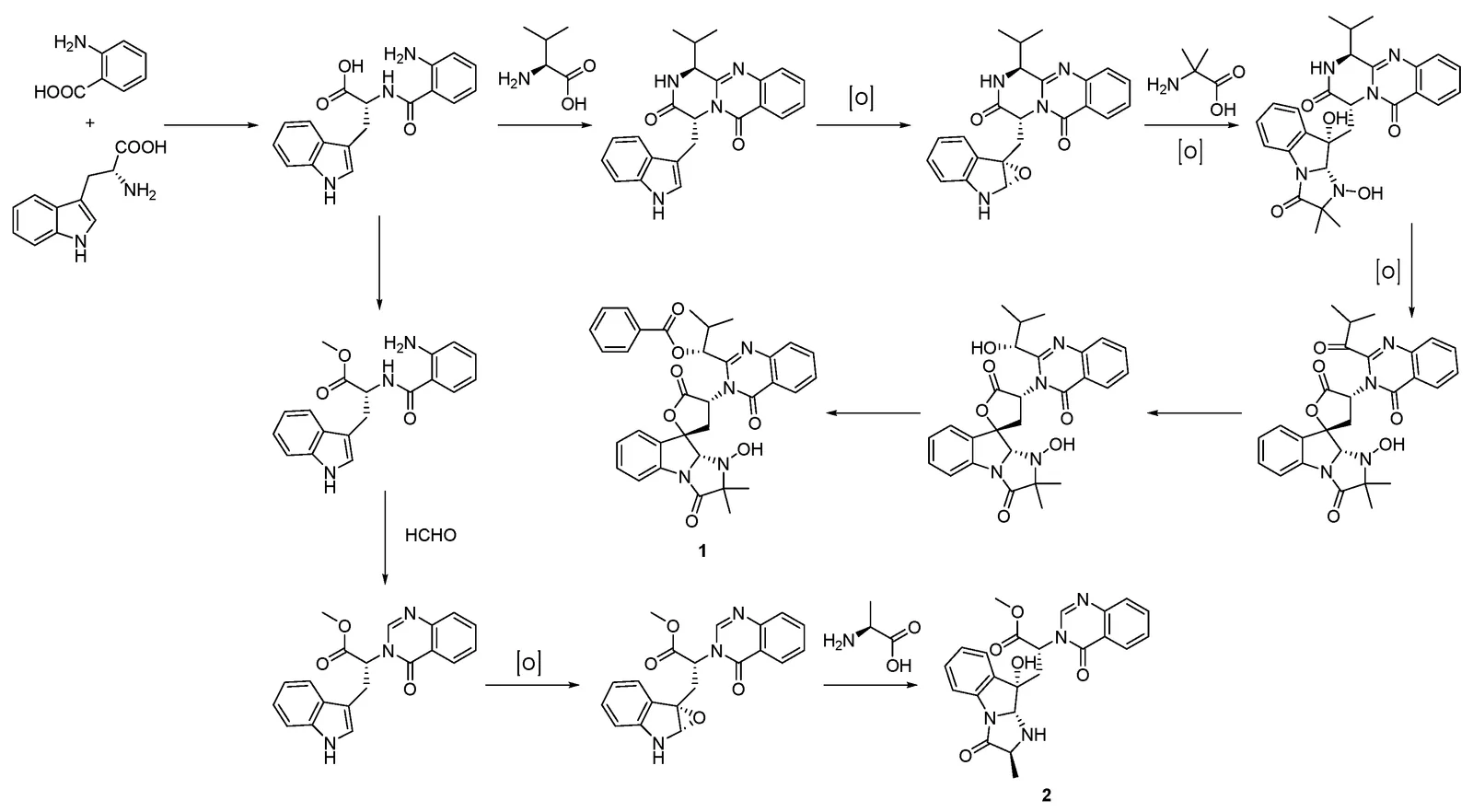
Proposed biosynthesis of **1** and **2**.

In this work, we proposed a hypothetical biosynthetic pathway for compounds **1** and **2** (Scheme 1). The pathway is initiated by the condensation of tryptophan (Trp) and anthranilic acid (Ant) along with valine (Val) by enzyme mediation to generate indoloquinazoline alkaloids. Epoxidation of the indole ring is a key enzymatic step for the formation of an imidazoindolone ring, in which the substrates include α-aminoisobutyric acid (Aib) for compound **1** and alanine (Ala) for compound **2**. Subsequent enzymatic oxidation of the pyrazinoquinazoline moiety furnishes the tryptoquivaline-type analogues featuring a spiro-γ-lactone unit. Compound **1** is then generated through sequential reduction and acylation reactions. For compound **2**, the Trp-Ant dipeptide undergoes *O*-methylation followed by condensation with formaldehyde to form another precursor. Subjecting this precursor to a similar epoxidation process and subsequent incorporation of alanine (Ala) ultimately yields compound **2**.

### 2.2. Antimicrobial, Antioxidant and Anti-Glycation Activities

**Figure 5:**
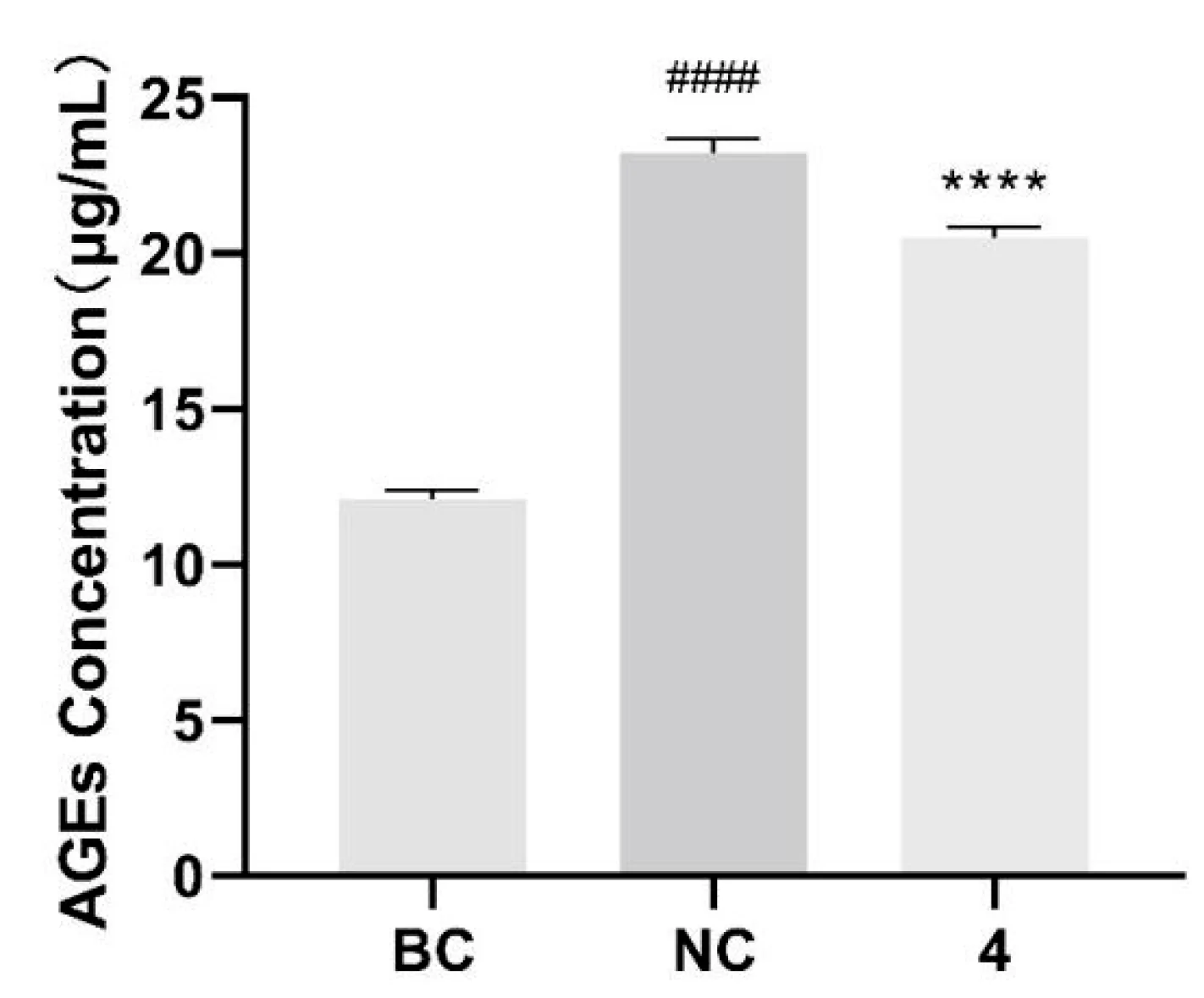
Compound **4** alleviates glucose-induced AGE production in HDF cells. HDF cells were treated with 125 mM glucose in the presence or absence of compound **4** (10 μM) for 24 h. AGE levels in the culture supernatants were measured by ELISA. Data are presented as mean ± SD (*n* = 3) (^####^ *p* < 0.0001 vs. BC group, **** *p* < 0.0001 vs. NC group).

Compounds **1**–**5** were tested for their anti-bacterial activity against Gram-positive (*Staphylococcus aureus*, *Staphylococcus epidermidis*) and Gram-negative (*Escherichia coli*, *Acinetobacter baumannii*, *Proteus mirabilis*) species, as well as anti-fungal activity against *Cryptococcus neoformans*, *Candida albicans*, and agricultural pathogens (*Pestalotiopsis theae*, *Neofusicoccum grevilleae*, *Colletotrichum musae*, *Thanatephorus cucumeris*); none of the compounds exhibited activity at 100 μM. Compounds **1**–**5** were also evaluated for their antioxidant capacity, and only **4** showed a relatively potent activity to scavenge free radicals with an IC_50_ value of 34.3 μM, comparable to ascorbic acid (IC_50_ = 30.5 μM). Additionally, compound **4** decreased glucose-induced AGE formation in dermal fibroblasts by 11.74% (Figure 5) at a 10 μM concentration, indicating a modest but significant anti-glycation effect.

### 2.3. Effects of Compounds ***1***–***5*** on MMP in PA-Induced AML-12 Cells

Mitochondria serve as the primary energy generators in cells and play a critical role in regulating cellular metabolism, cell cycle progression, and apoptosis [14,15]. Given that mitochondrial dysfunction is a key contributor to high-fat-induced hepatic injury [16], we next investigated whether the tested compounds could ameliorate mitochondrial dysfunction in a palmitic acid (PA)-induced AML-12 hepatocyte injury model. In this study, an AML-12 hepatocyte injury model induced by palmitic acid (PA) was established to mimic high-fat-induced hepatic damage. The treatment concentration of PA was chosen at 250 μM according to the previous study [17]. Meanwhile, cell viability assays confirmed that these compounds exhibited no cytotoxicity toward AML-12 cells at concentrations of 25, 12.5, and 6.25 μM.

**Figure 6:**
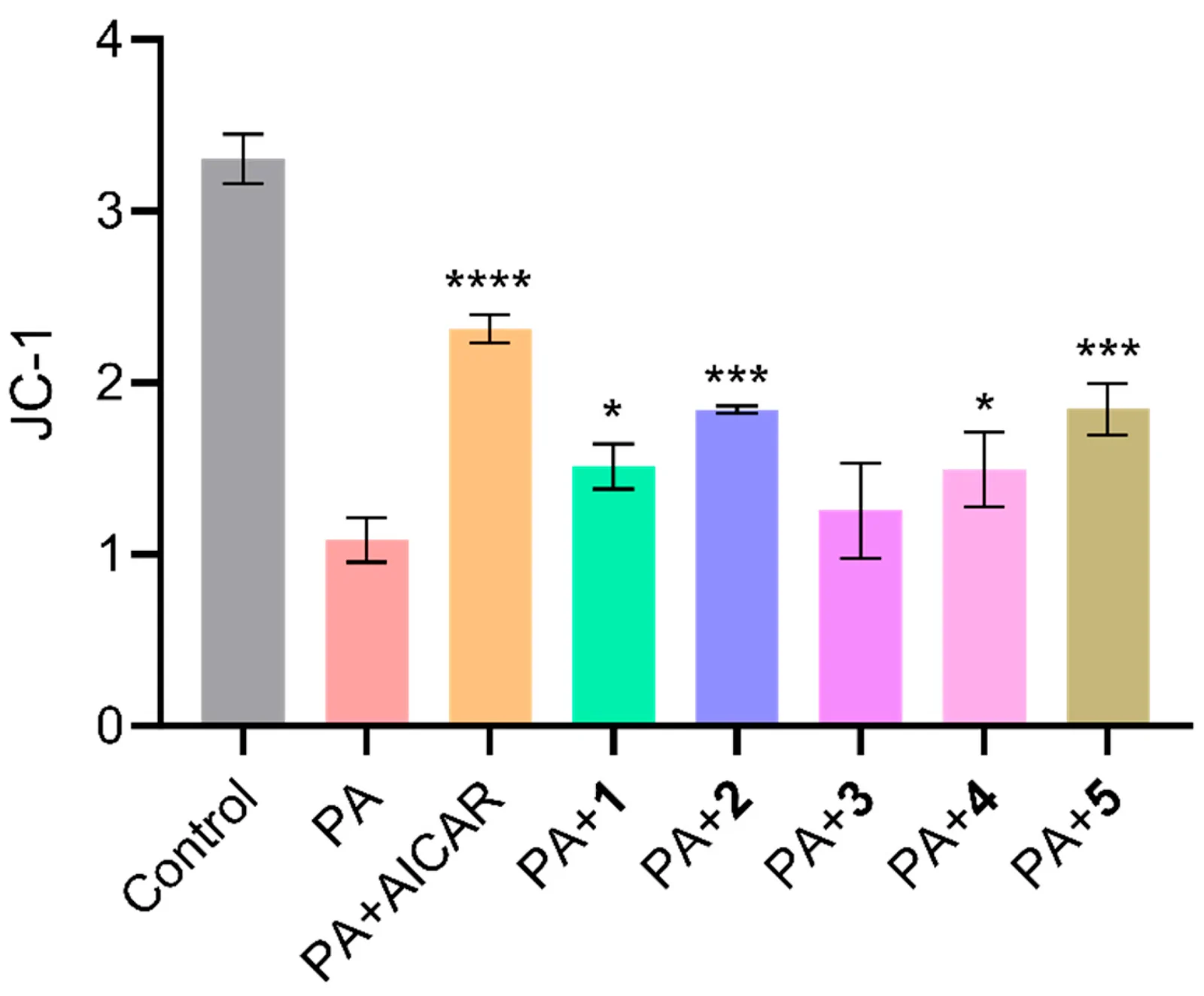
Effects of compounds **1**–**5** on mitochondrial membrane potential (MMP) in PA-induced AML-12 cells. AML-12 cells were treated with 250 μM palmitic acid (PA) in the presence or absence of the indicated compounds (12.5 μM) for 24 h. Acadesine (AICAR) was used as a positive control. MMP was assessed by JC-1 staining, and the red/green fluorescence intensity ratio was analyzed. A higher ratio indicates preserved MMP; a lower ratio indicates mitochondrial depolarization. Data are presented as mean ± SD (*n* = 3) (* *p* < 0.05, *** *p* < 0.001, **** *p* < 0.0001 vs. PA group).

To preliminarily assess the effects of the compounds on mitochondrial function, we performed JC-1 staining in AML-12 cells, as the JC-1 red/green fluorescence ratio is a sensitive and reliable indicator of MMP. A higher red/green ratio reflects preserved MMP and better mitochondrial function, whereas a decreased ratio indicates mitochondrial depolarization and dysfunction. In the model group, exposure to PA resulted in a marked decrease in the JC-1 ratio to 1.08, confirming the successful establishment of mitochondrial dysfunction in AML-12 cells. Among all tested compounds co-treated with PA, compounds **2** and **5** exhibited the most pronounced protective effect, significantly restoring the JC-1 ratio by approximately 34% compared to the model group (Figure 6). Acadesine was used as a positive control, exhibiting a 54% recovery compared to the PA-treated group. Although compounds **1**, **2**, **4** and **5** showed hepatoprotective activity, their effects were inferior to that of acadesine. Based on these screening results, compound **2** was selected for further mechanistic studies on mitochondrial function and oxidative stress. For subsequent experiments, three concentrations of compound **2** (6.25, 12.5, and 25 μM) were used.

### 2.4. Compound ***2*** Attenuates PA-Induced ROS Accumulation in AML-12 Cells

**Figure 7:**
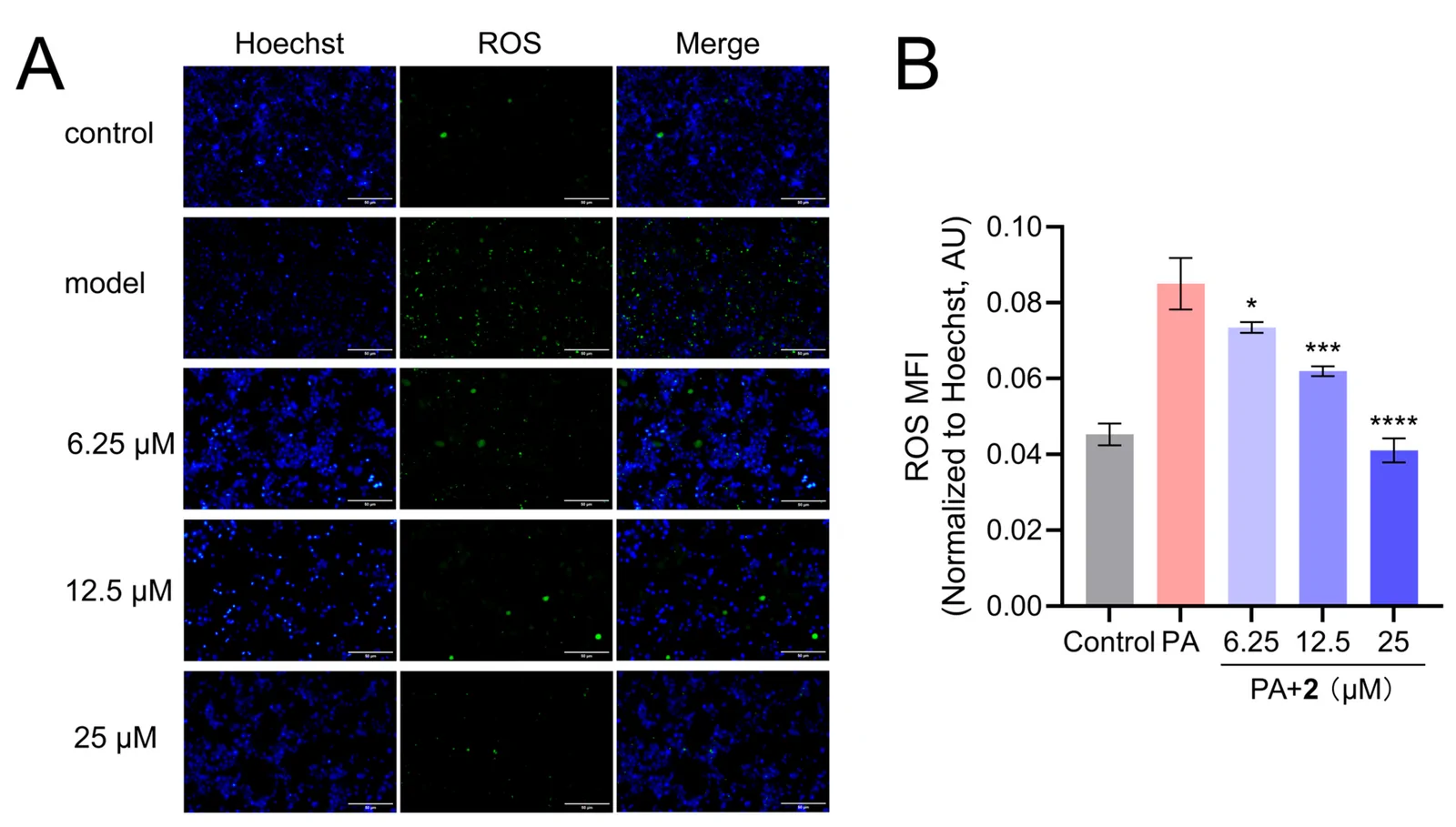
Compound **2** attenuates PA-induced ROS accumulation in AML-12 cells. (**A**) Representative fluorescence images of Hoechst 33342 (blue, nuclei) and ROS (green, DCFH-DA) staining in AML-12 cells treated with 250 μM PA in the presence or absence of compound **2** (6.25, 12.5, and 25 μM) for 24 h. Scale bar: 50 μm. (**B**) Quantitative analysis of ROS levels. The mean fluorescence intensity (MFI) of ROS was normalized to Hoechst fluorescence and expressed as arbitrary units (AUs). Data are presented as mean ± SD (*n* = 3) (* *p* < 0.05, *** *p* < 0.001, **** *p* < 0.0001 vs. PA group).

Excessive reactive oxygen species (ROS) accumulation leads to oxidative stress, cellular damage, and mitochondrial dysfunction, which are key features of high-fat-induced hepatocyte injury. Notably, Mitochondrial respiration is a major source of intracellular ROS [14,18]. To evaluate whether compound **2** could alleviate PA-induced oxidative stress in AML-12 cells, intracellular ROS levels were measured using the DCFH-DA fluorescent probe (Figure 7A). Hoechst 33342 was used to counterstain nuclei for normalization, and the results were expressed as mean fluorescence intensity (MFI) per nucleus. As shown in Figure 7B, exposure to 250 μM PA for 24 h significantly increased ROS levels compared to the control group. In contrast, co-treatment with compound **2** dose-dependently reduced the PA-induced ROS elevation. Notably, at the concentration of 25 μM, compound **2** restored ROS levels most effectively, nearly approaching those of the control group. These results indicate that compound **2** attenuates PA-induced oxidative stress in AML-12 cells, suggesting a protective role against lipotoxicity-related mitochondrial damage.

### 2.5. Compound ***2*** Restores ATP Production in PA-Treated AML-12 Cells

**Figure 8:**
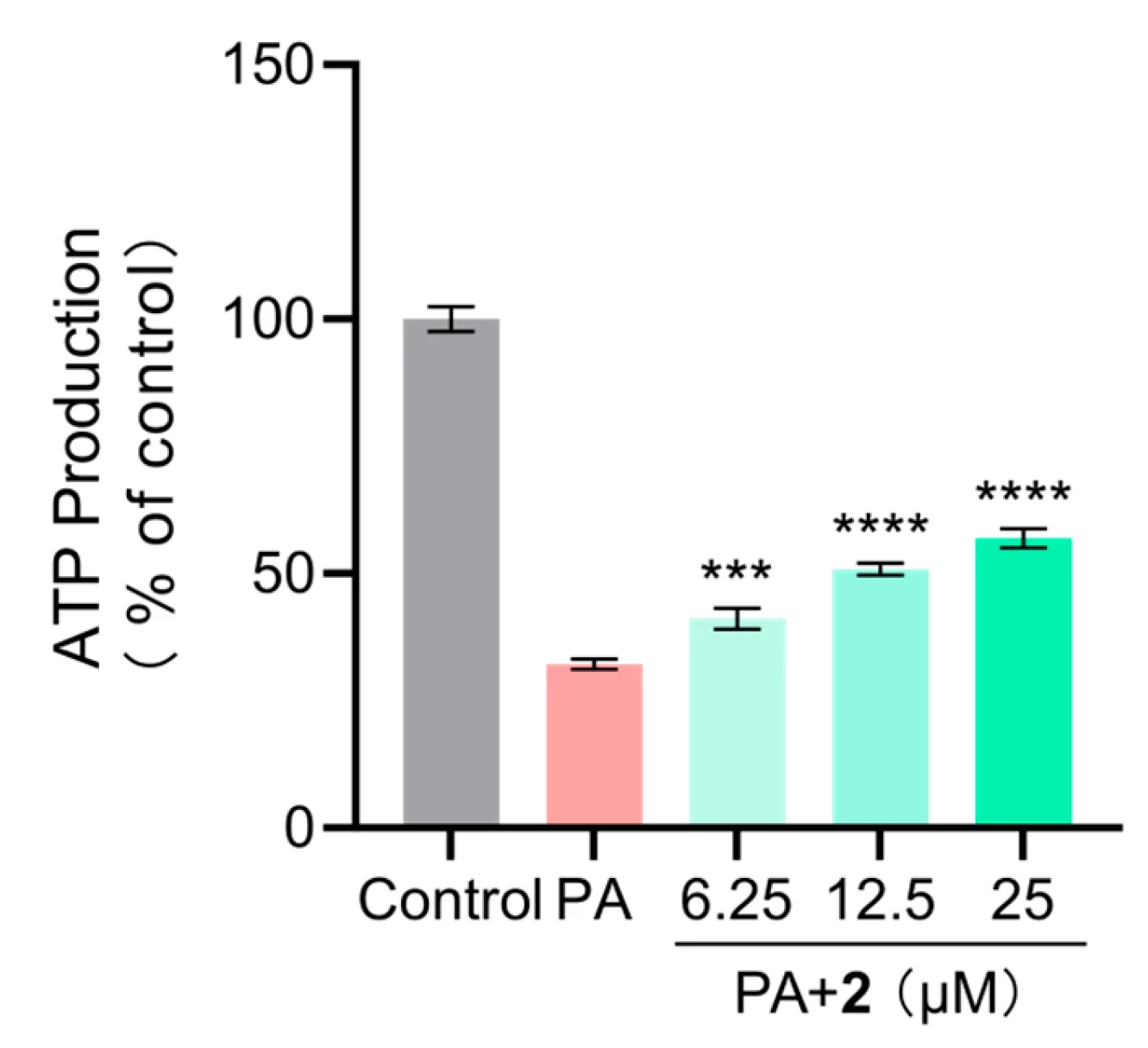
Compound **2** restores ATP production in PA-treated AML-12 cells. Cells were treated with 250 μM PA in the presence or absence of compound **2** (6.25, 12.5, and 25 μM) for 24 h. Data are expressed as percentage of the control group and presented as mean ± SD (*n* = 3) (*** *p* < 0.001, **** *p* < 0.0001 vs. PA group).

We next investigated whether compound **2** could restore ATP production in the PA-induced AML-12 hepatocyte injury model. As shown in Figure 8, exposure to 250 μM PA significantly reduced ATP levels to approximately 31.8% of the control group, indicating severe mitochondrial dysfunction. Co-treatment with compound **2** dose-dependently restored ATP production, with relative ATP levels of 43.4%, 49.5%, and 56.4% at concentrations of 6.25, 12.5, and 25 μM, respectively (all *p* < 0.001 vs. PA group). Collectively, these findings demonstrate that compound **2** attenuates PA-induced mitochondrial dysfunction by preserving ATP production and maintaining MMP in AML-12 cells.

### 2.6. Effects of Compounds ***1***, ***2***, and ***5*** on MMP in UVB-Induced HaCaT Cells

**Figure 9:**
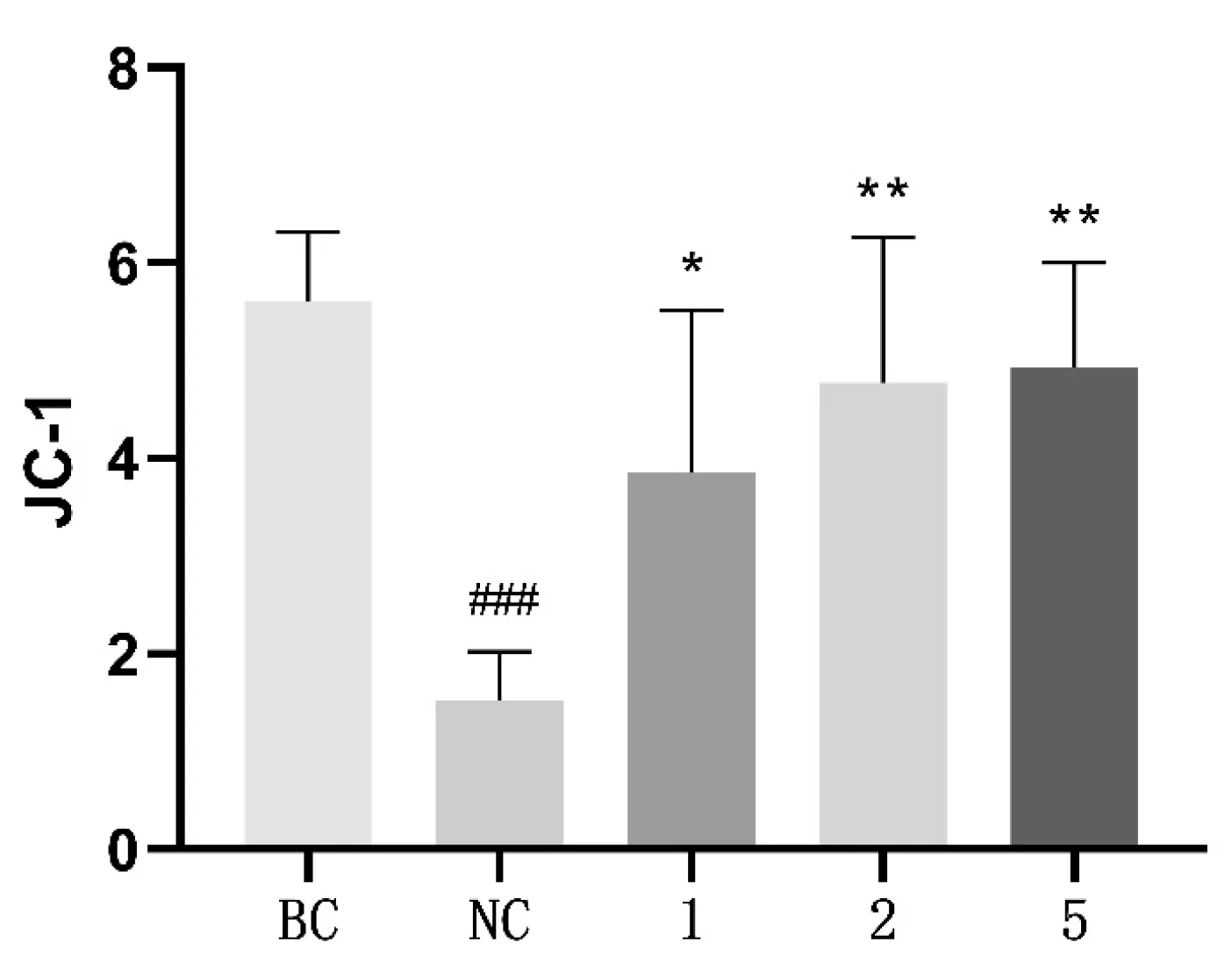
Effects of compounds **1**, **2**, and **5** on mitochondrial membrane potential (MMP) in UVB-induced HaCaT cells. HaCaT cells were exposed to UVB irradiation (30 mJ/cm^2^) and subsequently treated with compounds **1**, **2**, or **5** (10 μM) for 24 h. MMP was assessed by JC-1 staining, and the red/green fluorescence intensity ratio was analyzed. BC: blank control (no UVB, no treatment). NC: negative control (UVB only). Data are presented as mean ± SD (*n* = 3) (^###^ *p* < 0.001 vs. BC group; * *p* < 0.05, ** *p* < 0.01 vs. NC group).

In this study, a photodamage model was established in HaCaT cells using UVB irradiation. Compared with the blank control group, the red/green fluorescence ratio in the UVB model group was significantly decreased (*p* < 0.05), indicating that UVB irradiation at 30 mJ/cm^2^ reduced the mitochondrial membrane potential (MMP) and impaired mitochondrial function, thereby confirming the successful establishment of the photodamage model. Cytotoxicity assays confirmed that none of the tested compounds exhibited cytotoxic effects on HaCaT cells at a concentration of 10 μM. As shown in Figure 9, compared with the model control group, treatment with compounds **1**, **2**, and **5** significantly increased the red/green fluorescence ratio, restoring it to a level comparable to that of the blank control group. Compared with the model control group, the treatment groups showed a significant change in JC-1 relative fluorescence intensity, indicating that compounds **1**, **2**, and **5** possess anti-mitochondrial damage activity. These compounds protect skin cells from UV-induced damage and exert anti-photoaging effects.

### 2.7. Compounds ***1***, ***2*** and ***4*** Attenuate UVB-Induced ROS Accumulation in HaCaT Cells

**Figure 10:**
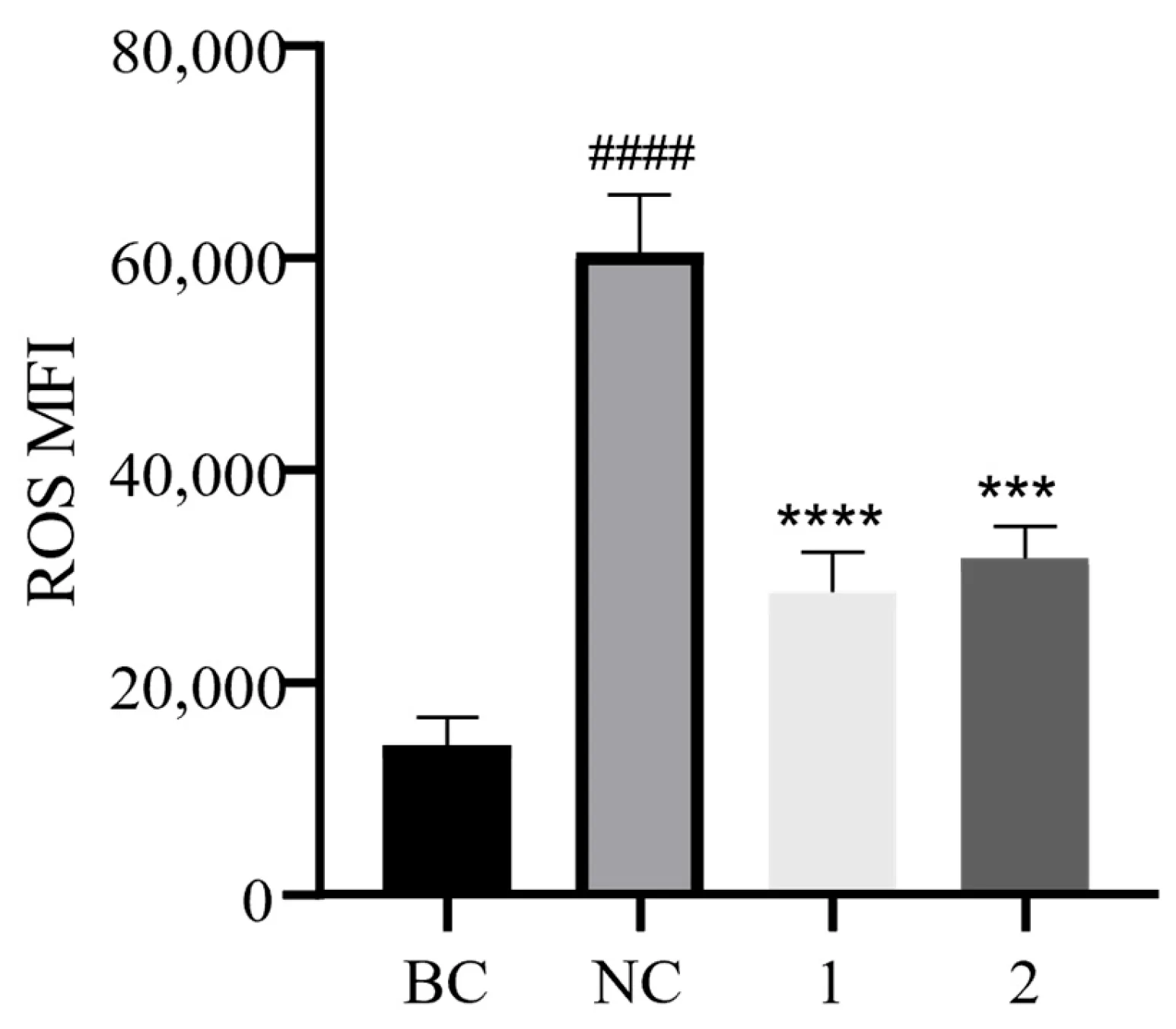
Effects of compounds **1** and **2** on UVB-induced ROS production in HaCaT cells. HaCaT cells were exposed to UVB irradiation (30 mJ/cm^2^) and subsequently treated with compound **1** or **2** (10 μM) for 4 h. Intracellular ROS levels were measured by flow cytometry using DCFH-DA staining (525 nm channel). BC: blank control (no UVB, no treatment). NC: negative control (UVB only). The geometric mean (GE) fluorescence intensity was used to quantify ROS levels. Data are presented as mean ± SD (*n* = 3) (^####^ *p* < 0.0001 vs. BC group, *** *p* < 0.001, **** *p* < 0.001 vs. NC group).

**Figure 11:**
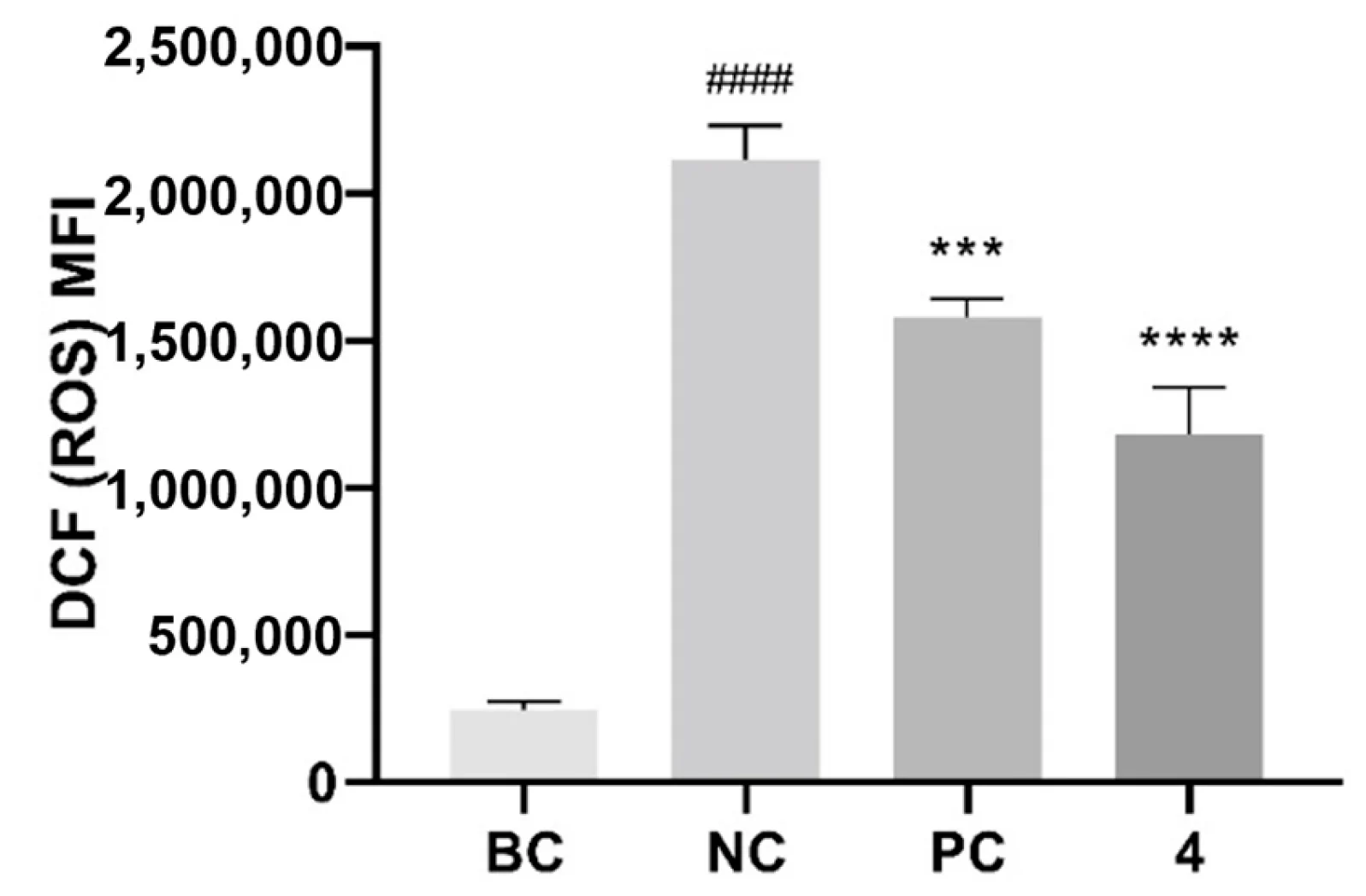
Effects of compound **4** on UVB-induced ROS production in HaCaT cells. HaCat cells were exposed to UVB irradiation (200 mJ/cm^2^) and subsequently treated with compound **4** (10 μM) or vitamin C (PC, 20 μg/mL) for a designated period. Intracellular ROS levels were measured by flow cytometry using DCFH-DA staining (525 nm channel). BC: blank control (no UVB, no treatment). NC: negative control (UVB only). Data are presented as mean ± SD (*n* = 3) (^####^ *p* < 0.0001 vs. BC group; *** *p* < 0.001, **** *p* < 0.0001 vs. NC group).

Compared with the blank control group, the mean fluorescence intensity of the UVB model group was significantly increased (*p* < 0.05), indicating that UVB irradiation at 30 mJ/cm^2^ successfully induced excessive intracellular ROS production. As shown in Figure 10, treatment with compound **1** or **2** significantly reduced the mean fluorescence intensity compared with the model group (*p* < 0.05), with ROS scavenging rates of 68.85% and 62.19%, respectively. Compound **4** reduced UVB-induced intracellular ROS levels in HaCaT cells, achieving a scavenging rate of 49.92%, which was superior to that of vitamin C (28.65% at 20 μg/mL) (Figure 11). These results demonstrate that compounds **1, 2** and **4** possess significant antioxidant activity and effectively scavenge UVB-induced excessive ROS.

### 2.8. System Equilibration and Global Membrane Modulation

To gain molecular-level insight into how compound **2** might influence mitochondrial membrane properties, we performed exploratory molecular dynamics (MD) simulations on a model membrane mimicking the lipid composition of the inner mitochondrial membrane under lipotoxic conditions (containing palmitic acid). These simulations are hypothesis-generating and should be interpreted with appropriate caution. To ensure thermodynamic stability prior to sampling, the root-mean-square deviation (RMSD) of the lipid phosphorus headgroups was monitored over the 1 μs trajectories (Supplementary Figure S32). The control membrane reached equilibrium at 558.5 ns, whereas the experimental system containing compound **2** equilibrated at 604 ns. Subsequent analyses were conducted utilizing the final, fully equilibrated 394 ns of both simulations. Representative convergence plots (RMSD over time) are provided in Figures S32 and S33. The observed changes in membrane structural parameters, while consistent with the in vitro functional rescue, are modest and derived from a single trajectory per system; replicate simulations and orthogonal biophysical experiments are needed to confirm these findings.

With stable baseline and experimental systems established, we investigated the macroscopic structural effects induced by the compound. Analysis of the global membrane dimensions revealed an increase in the global Area Per Lipid (APL) from 0.645 ± 0.010 nm^2^ in the control system to 0.658 ± 0.010 nm^2^ in the experimental system. Concurrently, the overall membrane thickness decreased from 4.03 ± 0.05 nm in the control state to 3.99 ± 0.05 nm in the experimental state (Supplementary Figure S33).

Together, this lateral expansion and transverse thinning demonstrate that the introduction of compound **2** induces significant macroscopic structural changes. These metrics provide the first line of evidence that compound **2** physically disrupts the tightly packed, palmitic acid-induced (PAL) lipotoxic membrane, expanding the bilayer into a more fluid and relaxed architectural state.

### 2.9. Insertion, Localization, and Orientation of Compound ***2***

**Figure 12:**
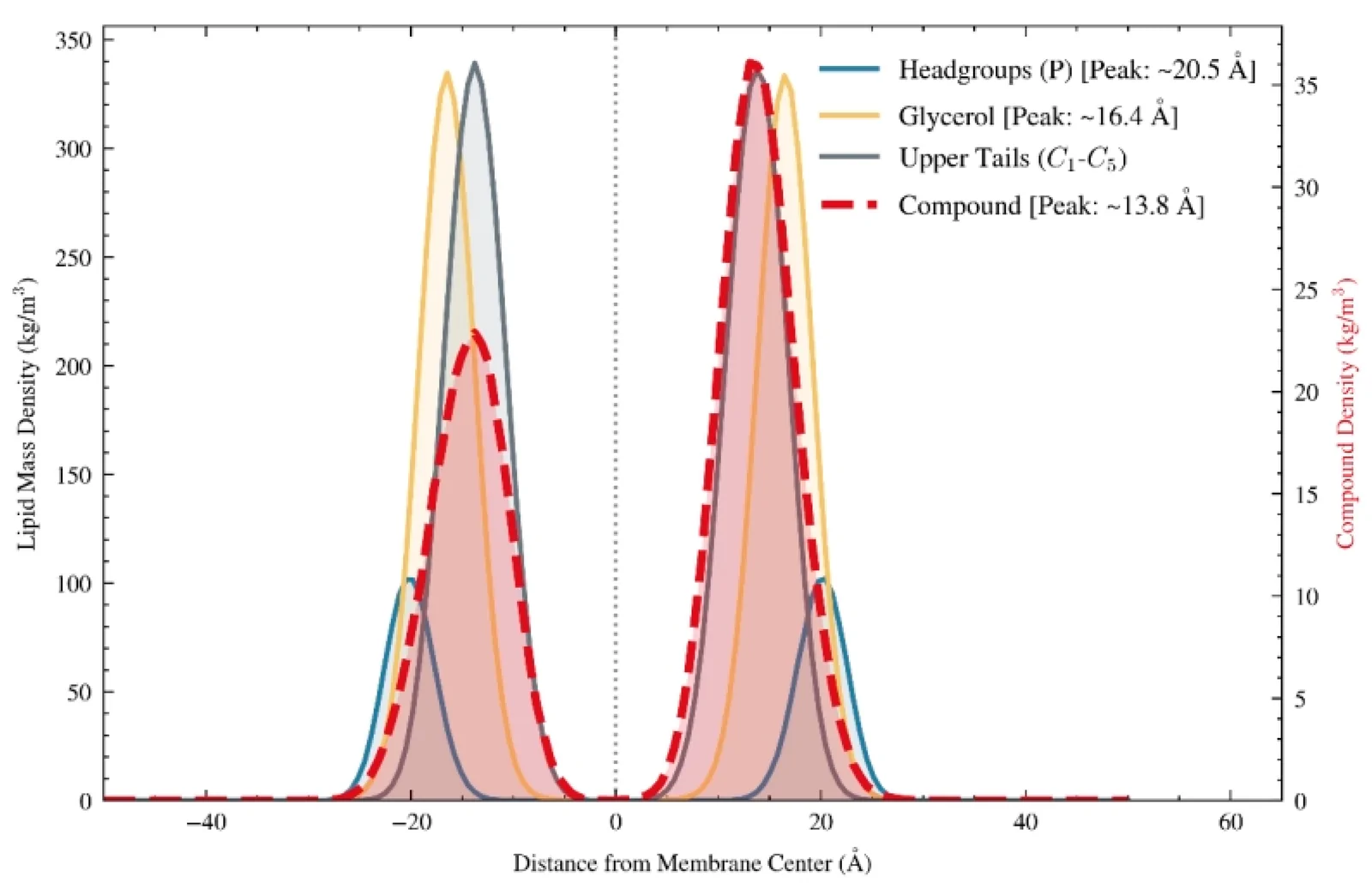
Transmembrane mass density profile defining the spatial localization of compound **2.** One-dimensional partial mass density distributions are plotted along the membrane normal, relative to the bilayer center (0 Å).

Analysis of the density distributions reveals that the compound preferentially partitions into the upper hydrophobic region, localizing just below the lipid–water interface. Specifically, the absolute mass density peak for compound **2** resides at ~13.8 Å from the membrane center. This insertion depth positions the molecule immediately beneath the lipid glycerol backbone, which exhibits a peak density at ~16.4 Å (Figure 12).

Within this shallow hydrophobic pocket, compound **2** adopts a highly stable and specific orientation. Measurements of the compound’s tilt angle relative to the membrane normal show a predominant distribution centering at 88.5 degrees (Supplementary Figure S34). This nearly 90-degree angle indicates that the molecule lies roughly parallel to the membrane plane, intercalating laterally between the upper segments of the lipid tails. This specific depth and lateral alignment are consistent with the compound’s electrostatic properties. The molecule possesses a total dipole moment of 0.99 Debye.

### 2.10. Preferential Interactions and Lipid Specificity

Having established that compound **2** localizes stably in the upper hydrophobic core, we next sought to define its specific intermolecular interaction network. On average, each compound coordinates a local solvation shell of ~9.17 bound lipid molecules. To understand how the compound anchors itself within this microenvironment, specific hydrogen bonding events were mapped using a 3.5 Å and >150° geometric cutoff. Trajectory analysis identified hydrogen atoms attached to C3-OH and C2-NH as the primary interacting factors, acting as a molecular “claw” to anchor the compound to lipid headgroups. In absolute terms, the highest observed hydrogen bond occupancy was formed between the compound’s C3-OH donor and the POPC O22 acceptor, remaining active for 9.0% of the simulated timeframe per compound (1774 frames) (Supplementary Table S5).

However, absolute occupancy is inherently biased by the bulk concentration of the membrane (where POPC constitutes 45% of the total lipids). To mathematically isolate the compound’s true thermodynamic binding preference, Preferential Binding Enrichment Scores were calculated for both hydrogen bonding and general steric/Van der Waals (VdW) contacts using the Enrichment Factor formula. This metric normalizes the local concentration of a lipid species around the compound against its global concentration in the bulk membrane. A score of 1.0 indicates neutral, concentration-driven binding; a score > 1.0 indicates active preferential targeting; and a score < 1.0 indicates active avoidance.

**Figure 13:**
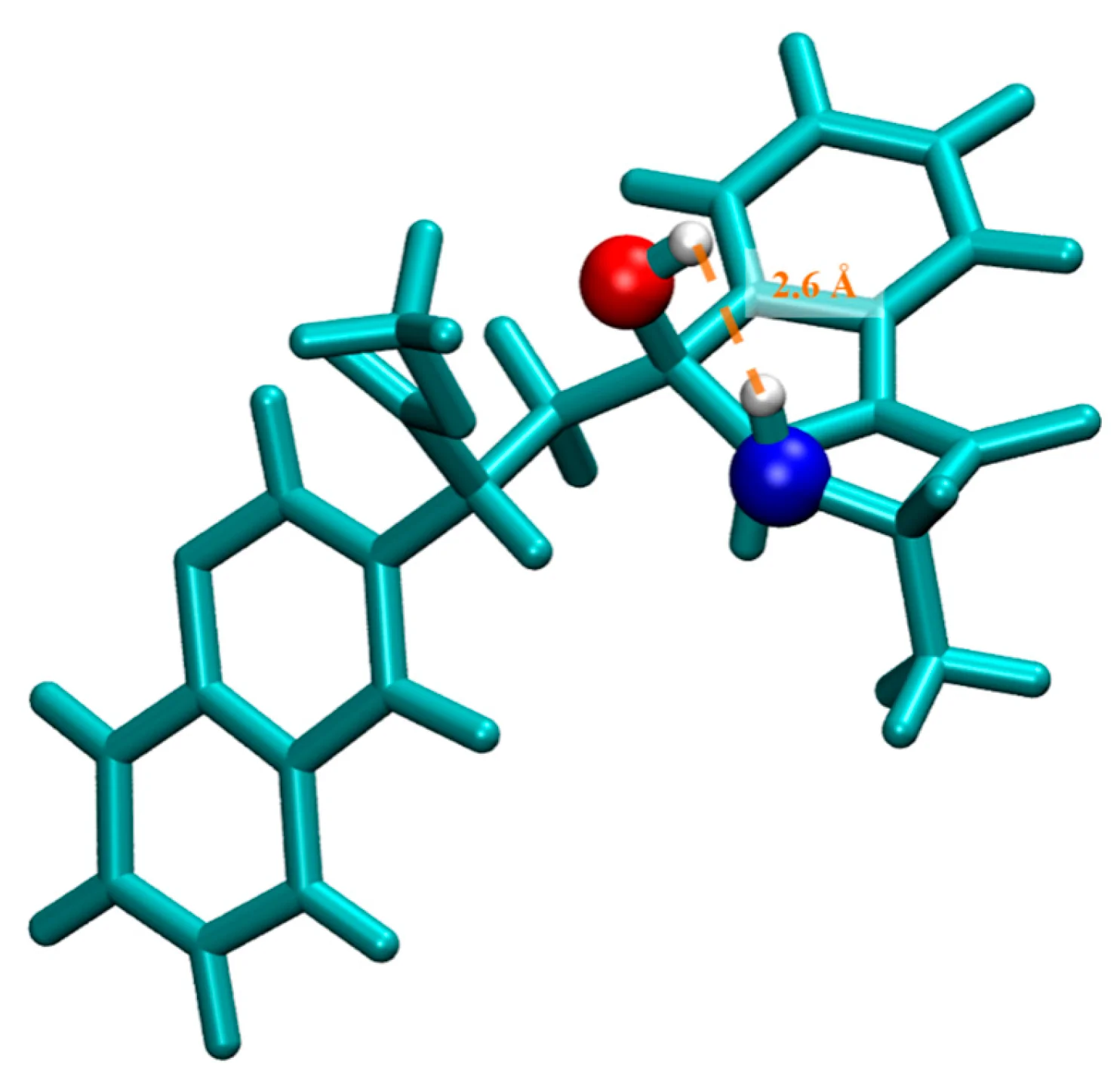
Structural architecture and primary binding interface of compound **2.** The terminal functional groups responsible for cardiolipin (TLCL2) selectivity are highlighted. Enlarged are the C3-OH (red) and C2-NH (blue) atom pairs, which collectively form the sterically complementary “clamp” motif. This specific dual-anchor configuration enables the simultaneous multi-point coordination of both the superficial and deep interfacial regions of the TLCL2 headgroup.

**Figure 14:**
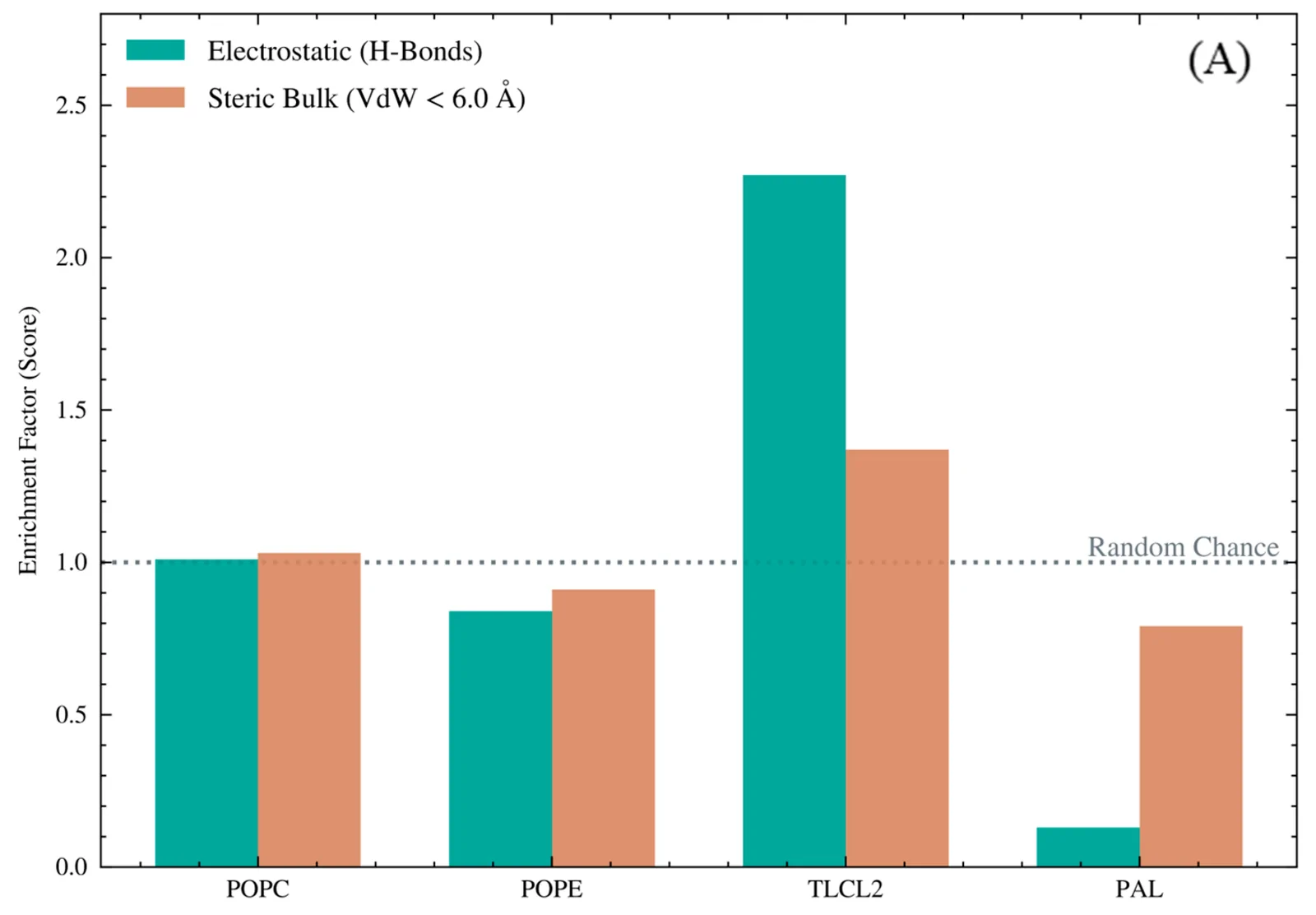

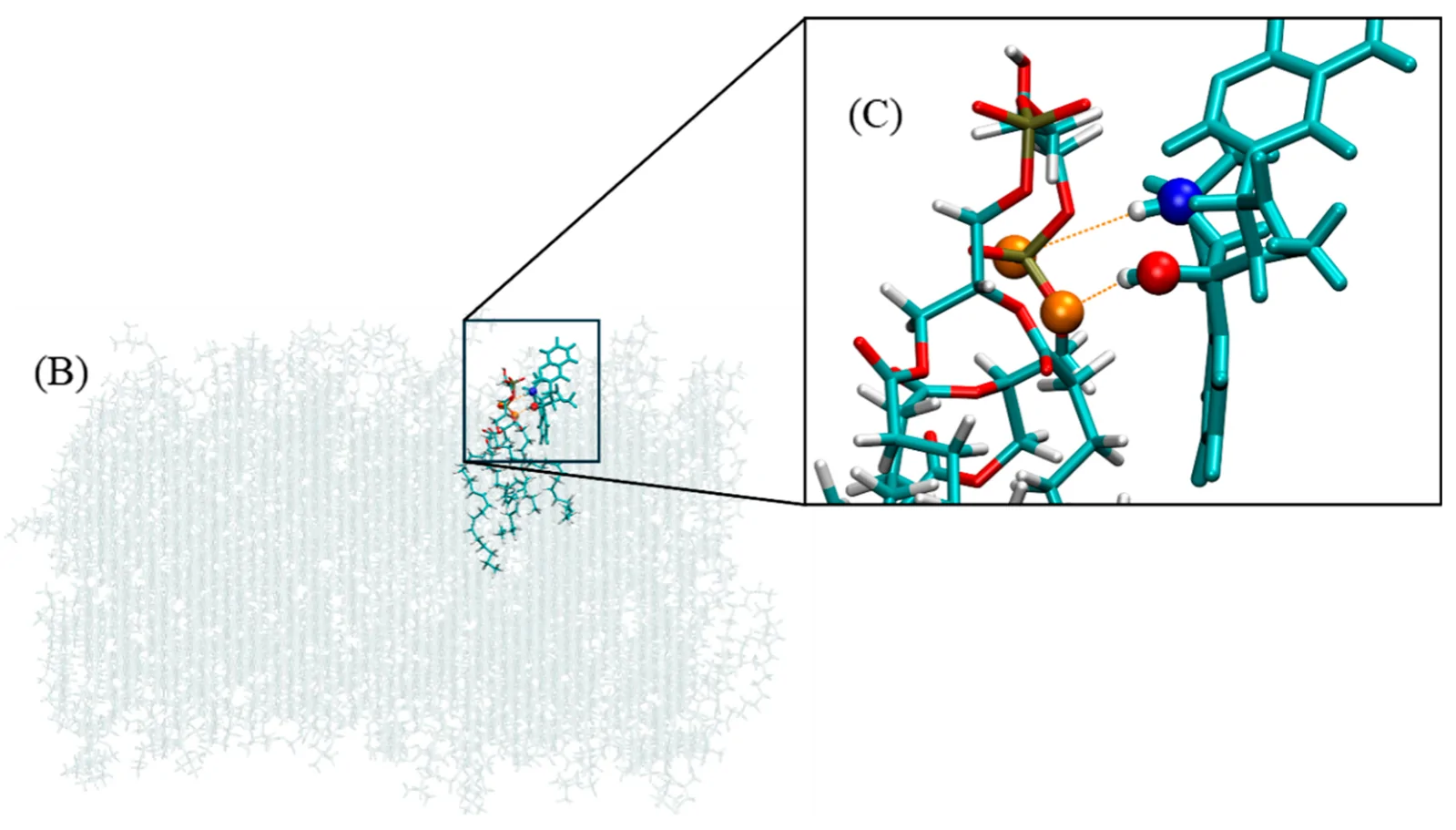
Preferential interaction and structural anchoring of compound **2** within the mitochondrial membrane model. (**A**) Preferential Binding Enrichment Scores evaluating the compound’s thermodynamic binding preference for specific lipid species. Scores are normalized against the bulk membrane molar composition, with the dashed line at 1.0 representing neutral, concentration-driven distribution. The plot contrasts the strong selectivity for cardiolipin (TLCL2) against the spatial exclusion of palmitic acid (PAL) across both hydrogen bonding and van der Waals (VdW) interaction profiles. (**B**) Macroscopic 3D simulation snapshot of the membrane embedding environment. The bulk heterogeneous bilayer is rendered transparently to visualize the compound’s localized lipid solvation shell and spatial segregation. (**C**) High-resolution structural detail of the active TLCL2 targeting interface. The spatial arrangement highlights the geometrically complementary “clamp”, with dashed lines (yellow) denoting the hydrogen-bonding events between the compound’s terminal anchors (C3-OH/C2-NH; in white) and the superficial TLCL2 phosphate oxygens (OP12/OP14; in orange), facilitating the simultaneous coordination of deeper interfacial ester carbonyls.

The enrichment analysis supports a cardiolipin-associated membrane-modulation mechanism. While general VdW interactions demonstrated a baseline enrichment for TLCL2 (cardiolipin) at 1.37, the hydrogen-bonding enrichment profile was remarkably selective. The C3-OH/C2-NH “claw” exhibited a massive 2.27-fold preference for hydrogen bonding with TLCL2. Detailed atom-to-atom hydrogen bond analysis revealed that this selectivity is driven by a highly coordinated, multi-point binding interface. The spatial distance between the compound’s terminal functional groups—specifically the C3-OH and C2-NH pairs (2.6 ± 0.6 Å)—provides a geometrically compatible “clamp” for the cardiolipin headgroup (Figure 13). This motif anchors compound **2** by dynamically engaging two distinct depths of the lipid interface. As shown in Figure 14, the C3-OH and C2-NH donor groups strongly coordinate the negatively charged superficial phosphate oxygens (primarily OP12 and OP14), while simultaneously forming high-occupancy hydrogen bonds deeper in the hydrophobic core with the ester carbonyls (OB1, OC1, and OA1) of the TLCL2 glycerol backbone (Supplementary Table S6). This simultaneous engagement of both the deep carbonyls and the superficial phosphates provides a structural rationale for the compound’s observed preference for cardiolipin. Conversely, compound **2** appeared to avoid palmitic acid (PAL), yielding strongly depleted enrichment scores of 0.79 for VdW contacts and a mere 0.13 for hydrogen bonding.

Together, these metrics suggest a preferential interaction pattern: compound **2** does not distribute randomly. Driven by the multi-point hydrogen-bonding capacity of its functional anchors, the compound appears to preferentially interact with cardiolipin molecules while showing reduced association with the rigid PAL domains.

### 2.11. Local Membrane Fluidization and Structural Disruption

A comparison of the calculated deuterium order parameters (*S_CD_*) between the bulk membrane lipids and those actively bound in the compound’s solvation shell (<6.0 Å) revealed a distinct mechanical shift. The acyl tails of the bound POPC molecules exhibited a significantly decreased *S_CD_* compared to the bulk POPC (Supplementary Figure S34A). This reduction in order indicates that the compound’s intercalation into the upper hydrophobic core physically forces the neighboring lipid tails to adopt more disordered, gauche conformations, effectively melting the rigid packing.

The selective localization and flat orientation of the compound directly translate into targeted structural disruption of the lipotoxic environment. To quantify this fluidization, deuterium order parameters *S_CD_* were calculated vectorially for the acyl tails of POPC (sn-1; sn-2), POPE (sn-1; sn-2), and TLCL2 (two tails—A and B) (Supplementary Figure S35B–D). The analysis revealed a global decrease in *S_CD_* across all lipid tails in the experimental system compared to the control.

The localized fluidization was further corroborated by mapping the lateral spatial distribution of the lipids. Local APL analysis via 2D Voronoi tessellation demonstrated that the bulk lipids (>12.0 Å) maintained a tightly packed mean APL of 0.822 ± 0.263 nm^2^. In stark contrast, the lipids actively bound to compound **2** exhibited a locally expanded mean APL of 0.940 ± 0.288 nm^2^ (Supplementary Figure S35E). This 14.3% localized expansion physically forces adjacent lipid tails apart, disrupting pathological packing geometries [19,20] and imparting critical fluidity to the previously rigid environment.

S_cd_ analyses on the localized and on the global level are consistent with the hypothesis that fluidization originates at the compound–lipid interface and propagates throughout the bilayer.

### 2.12. Modulation of the Membrane Electrostatic Environment

The macroscopic fluidization and localized spatial expansion induced by the compound carry profound functional consequences for the mitochondrial membrane. To understand the energetic basis of these structural changes, the spatial electrostatic potential (*ϕ*(*z*)) was mapped across the *Z*-axis for both systems. In the control system, the high degree of order heavily aligns the lipid ester carbonyls and interfacial water molecules, resulting in a notably high maximum dipole potential of 0.676 V. Biologically, this abnormal rigidity impairs the conformational dynamics of electron transport chain proteins or induces phase separations that leak protons, leading to the collapse of the transmembrane potential frequently observed in lipotoxic models [21,22].

Upon integration into the membrane, the compound acts as an electrostatic disruptor. The molecule possesses a highly specific negative Z-component projection of the dipole moment (*μ_z_*) averaging −0.379 Debye. Because of this negative vertical dipole, the compound actively antagonizes the highly aligned, rigid electrostatic architecture of the PAL-containing membrane. By physically forcing the lipids apart and increasing local fluidity, the compound disrupts the uniform alignment of the interfacial dipoles. In membrane biophysics, a reduction in the internal dipole potential is a classic, measurable hallmark of this increased fluidization [23,24]. Validating this mechanism, the maximum electrostatic potential in the experimental system dropped from 0.676 V to 0.658 V (Supplementary Figure S35). We hypothesize that by fluidizing the rigidified domains, the compound reconstitutes the physical and electrostatic membrane state necessary for proper protein function and proton gradient maintenance. Consequently, this localized structural rescue is consistent with the macroscopic repolarization of the mitochondrial membrane observed in vitro, further supporting a cardiolipin-associated membrane-modulation mechanism.

### 2.13. Limitations of Mechanistic Interpretation

The MD results presented here are based on a single 1-μs trajectory per system. While the simulations reached stable equilibration (RMSD plateaus after 558.5 ns for control and 604 ns for experimental), the observed changes in APL (0.645 to 0.658 nm^2^) and thickness (4.03 to 3.99 nm) are small. Therefore, these simulations should be viewed as supportive, not definitive, evidence for the proposed membrane-modulation mechanism. Future validation should include (1) surface plasmon resonance (SPR) or isothermal titration calorimetry (ITC) to measure cardiolipin binding affinity, (2) fluorescence anisotropy to assess membrane fluidity changes, and (3) molecular dynamics simulations with replicate trajectories and enhanced sampling methods to improve statistical robustness.

## 3. Materials and Methods

### 3.1. Isolation of Secondary Metabolites

#### 3.1.1. General Experimental Procedures

Optical rotations (ORs) were measured in MeOH on a JASCO P-2000 polarimeter (JASCO Corporation, Tokyo, Japan). UV were measured in MeOH on a Shimadzu UV-1800 scan spectrophotometer (Shimadzu Corporation, Kyoto, Japan). ECD spectra were recorded on a JASCO J-715 circular dichroism spectrometer (JASCO Corporation, Tokyo, Japan). IR spectra were recorded on a Nicolet IS 10 FT-IR spectrometer (Thermo Fisher Scientific, Waltham, MA, USA). One- and two-dimensional NMR data were obtained on a JEOL 600 MHz spectrometer (JEOL Ltd., Tokyo, Japan). Compounds were analyzed in DMSO-*d*_6_. Chemical shifts (^1^H and ^13^C) are expressed in *δ* (ppm). HRESIMS spectra were acquired from AB Sciex 5500 Q-TRAP (AB Sciex Pte. Ltd., Framingham, MA, USA) and LC/MS Q-Orbitrap (Q Exactive Focus, Thermo Fisher Scientific, Waltham, MA, USA). Semipreparative HPLC was performed on a U-3000 system (Chuangxin Tongheng Co., Ltd., Beijing, China) equipped with a Venusil RP-C18 column (10 mm × 250 mm, 10 μm, Agela Technologies, Torrance, CA, USA) at a flow rate of 10.0 mL/min. TLC was performed using Merck precoated plates (Silica gel HSGF254, 0.25 mm thickness; Merck KGaA, Darmstadt, Germany) of 0.25 mm thickness. Column chromatography (CC) was performed with silica gel (48–75 μm, Qingdao Haiyang Chemical Co., Ltd., Qingdao, China) or Lobar LiChroprep RP-C18 (ODS, 40–63 μm, Merck KGaA, Darmstadt, Germany).

#### 3.1.2. Fungal Material

The fungus *Aspergillus clavatus* C2WU was separated isolated from hydrothermal vent crab *Xenograpsus testudinatus*, which was collected from Kueishantao, Taiwan, and identified by its ITS-5.8s rDNA sequences. The surface of the shell of the crab sample was rinsed three times with sterile artificial seawater. The surface of the shell tissue was excised with a sterile scalpel and scraped into powder. Powder was homogenized using a blender containing 20 mL sterile natural seawater in aseptic conditions. The resulting homogenate was diluted with sterile seawater (1:5, 1:25, 1:125, 1:625). Under sterile conditions, 200 µL of each dilution was inoculated in quadruplicate onto GYP, containing 1.0 g glucose, 0.1 g yeast extract, 0.5 g peptone, 15 g agar per liter seawater. The plates were incubated at room temperature for 1–3 weeks until the morphology of fungi could be distinguished. Each isolate was picked. Pure strains of *A. clavatus* C2WU were isolated by reinoculation on agar plates. Subcultures of the organism are deposited at China Center for Type Culture Collection (CCTCC) (No. CCTCC M 201342).

#### 3.1.3. Fermentation, Extraction, and Isolation

*Aspergillus clavatus* C2WU grown on PDA (Potato Dextrose Agar) medium (0.5 cm × 0.5 cm) was added to the solid rice medium about 700 Erlenmeyer flasks at 28 °C for 30 days incubation.

The samples were extracted with ethyl acetate for three times. Then, the ethyl acetate was removed under vacuum using a rotary evaporator. About 105 g crude extract was obtained from the fermentation product. The crude extract of C2WU was subjected to silica gel liquid chromatography using a mixture of petroleum ether (PE) and ethyl acetate (EA) followed by CH_2_Cl_2_-MeOH as gradient elution solvent, which afforded 12 fractions (Fr. 1–12) based on the result of TLC analyses. Compound **1** (Rt. 29 min, 2.0 mg) was further purified by pre-HPLC (MeOH-H_2_O 85:15, 40 min) in Fr. 4. Fr. 12 was separated using pre-HPLC (MeOH-H_2_O 55:45, 40 min) to produce compounds **2** (Rt. 27 min, 4.0 mg) and **4** (Rt. 11 min, 4.5 mg). After the separation of subfraction Fr. 8 (MeOH-H_2_O 75:25, 40 min), compound **3** (Rt. 30 min, 5.1 mg) was obtained.

Tryptoquivaline Z_1_ (**1**): white powder; ${\left[\alpha \right]}_{D}^{20}\;$–16.0 (*c* 0.05, MeOH); UV (MeOH) *λ*_max_ (log *ε*) 192 (1.36), 233 (0.57), 279 (0.14) nm; ECD (0.5 mg/mL, MeOH) *λ*_max_ (Δ*ε*) 205 (+14.62), 227 (–1.07), 235 (–0.20), 247 (–5.94), 278 (+0.24) nm; IR *v*_max_ 3420, 68, 2931, 2873, 1786, 1728, 1682, 1598, 1484, 1468, 1251, 1185, 1132, 1108, 1071, 758, 711, 620 cm^−1^; ^1^H and ^13^C NMR (600 MHz, DMSO-*d*_6_) data are shown in Table 2; HRESIMS *m*/*z* 609.2345 [M + H]^+^ (calcd for C_34_H_33_N_4_O_7_; 609.2344).

Clavutoine V (**2**): white powder; ${\left[\alpha \right]}_{D}^{20}\;$+4.0 (*c* 0.05, MeOH); UV (MeOH) *λ*_max_ (log *ε*) 192 (0.78), 200 (0.62), 226 (0.41) nm; ECD (0.5 mg/mL, MeOH) *λ*_max_ (Δ*ε*) 214 (+6.42), 229 (–5.19) nm; IR *v*_max_ 3344, 2928, 1678, 1608, 1483, 1440, 1389, 1325, 1298, 1266, 1109, 773, 701 cm^−1^; ^1^H and ^13^C NMR (600 MHz, DMSO-*d*_6_) data are shown in Table 2; HRESIMS *m*/*z* 435.1663 [M + H]^+^ (calcd for C_23_H_23_N_4_O_5_; 435.1663).

Cytochalasin Z_29_ (**3**): white powder; ${\left[\alpha \right]}_{D}^{20}\;$–25.6 (*c* 0.05, MeOH); UV (MeOH) *λ*_max_ (log *ε*) 196 (3.67), 219 (0.65) nm; ECD (0.5 mg/mL, MeOH) *λ*_max_ (Δ*ε*) 230 (–6.97), 251 (+1.44), 301 (–6.67) nm; IR *v*_max_ 3390, 2965, 2930, 1766, 1710, 1455, 1319, 1231, 1117, 1020, 966, 932, 701 cm^−1^; ^1^H and ^13^C NMR (600 MHz, DMSO-*d*_6_) data are shown in Table 3; HRESIMS *m/z* 480.2379 [M + H]^+^ (calcd for C_28_H_34_NO_6_; 480.2381).

Methyl (*S*)-2-(2,5-dihydroxyphenyl)-2-methoxyacetate (**4**): brown oil; ${\left[\alpha \right]}_{D}^{20}\;$–10.4 (*c* 0.05, MeOH); UV (MeOH) *λ*_max_ (log *ε*) 195 (2.37), 235 (0.54) nm; ECD (0.5 mg/mL, MeOH) *λ*_max_ (Δ*ε*) 195 (–0.83), 197 (+3.34), 202 (–1.26), 206 (+0.92) nm; IR *v*_max_ 3243, 2954, 1736, 1599, 1506, 1455, 1352, 1200, 1085, 1047, 1023, 1003, 823, 765 cm^−1^; ^1^H and ^13^C NMR (600 MHz, DMSO-*d*_6_) data are shown in Table 4; HRESIMS *m*/*z* 235.0574 [M + Na]^+^ (calcd for C_10_H_12_NaO_5_; 235.0577).

#### 3.1.4. ECD Computational Analysis

Conformational search was accomplished using the MMFFs force field with the conformational search using an energy window of 10 kJ/mol by the Spartan 14 program. Conformers that were above 1% Boltzmann populations were re-optimized at the PBE1PBE/6-311G(d) level with PCM models by ORCA 5.0.3 [25]. Frequency analysis was also performed to confirm that the re-optimized geometries were at the energy minima. The optimized conformations, whose Boltzmann distributions of Gibbs free energies were more than 1.0%, were used for the ECD calculations using the TD-DFT method with the basis set wB97XD/def2-TZVP. Eventually, the Boltzmann-averaged ECD spectra of the compounds were obtained with SpecDis 1.71 [26].

### 3.2. Biological Activities of the Isolated Compounds

#### 3.2.1. Antimicrobial Assay

Antimicrobial assay was performed as described previously [27]. Cycloheximide was used as the positive control. Fungi and bacteria were cultivated in PDB and LB, respectively. Then, the test compounds were dissolved and diluted to different concentrations on a 96-well plate, and an aliquot (10 μL) of microbial suspension was added to each well. The MIC was recorded after incubating at 30 °C for 24 h.

#### 3.2.2. Antioxidant Activity by DPPH Assay

Measurement of DPPH radical scavenging activity. Various concentrations of the stock solutions (diluted to final concentrations of 100, 50, 25, 12.5, 6.25 and 3.125 μM) were mixed with 0.25 mM DPPH (Shanghai Macklin Biochemical Technology Co., Ltd., Shanghai, China) in ethanol to produce a final DPPH concentration of 0.1 mM. The mixture was vigorously shaken and left to stand for 10 min in the dark, and its absorbance was measured at 517 nm. L-Ascorbic acid was used as the positive control [28].

$$DPPH\;scavenging\;efficiency\;(\%)\;=\;[1-\frac{A-C}{B}]\times 100\%$$

A is the absorbance of the sample at 517 nm wavelength.
B is the absorbance of the control group at 517 nm wavelength.
C is the absorbance of the sample blank at 517 nm wavelength.

#### 3.2.3. Cell Culture and General Experimental Conditions

Mouse AML-12 hepatocytes were obtained from the Chinese Academy of Sciences Cell Bank (Shanghai, China) and cultured in F12 medium supplemented with 10% fetal bovine serum (FBS; Sijiqing Bioengineering Materials Co. Ltd., Hangzhou, China), 1% penicillin/streptomycin (P/S; Sigma-Aldrich, St. Louis, MO, USA), 1% ITS liquid medium supplement (Sigma, St. Louis, MO, USA), and 40 ng/mL of dexamethasone. HaCaT cells (human keratinocytes) and HDF cells (human dermal fibroblasts) were obtained from the American Type Culture Collection (ATCC) and cultured in DMEM supplemented with 10% FBS and 1% P/S.

All cells were maintained at 37 °C in a humidified incubator with 5% CO_2_. For all cell-based assays, cells were seeded at the following densities: AML-12 at 3.0 × 10^5^ cells/well (12-well plates) or 1.0 × 10^4^ cells/well (96-well plates); HaCaT at 3.0 × 10^5^ cells/well (12-well plates); HDF at 2.0 × 10^5^ cells/well (6-well plates). Stock solutions of test compounds (100 mM) were prepared in DMSO and diluted with culture medium to the desired concentration prior to use. The final DMSO concentration in all samples was kept below 0.1% (*v*/*v*). All experiments were performed in three independent biological replicates, each with three technical replicates (*n* = 3). Data are presented as mean ± standard deviation (SD). Statistical significance was determined using one-way ANOVA followed by Student’s *t*-test for two-group comparisons, using GraphPad Prism 9.0. Significance levels are denoted as * *p* < 0.05, ** *p* < 0.01, *** *p* < 0.001, and **** *p* < 0.0001. All cell lines were handled according to institutional biosafety guidelines. The fungal strain *Aspergillus clavatus* C2WU was isolated from environmental samples collected from non-protected areas (Kueishantao, Taiwan) and does not involve ethical concerns.

Palmitic acid (PA) treatment. To simulate fatty liver disease model, AML-12 cells were exposed to 250 μM PA (Sigma, USA) for 24 h. Palmitic acid was complexed with fatty-acid-free BSA (Beijing Solarbio Science & Technology Co., Ltd., Beijing, China). Briefly, palmitic acid was dissolved in 1× PBS, and a 250 mM stock solution was obtained following various cycles of incubation in a water bath at 70 °C and vortex. The stock solution was then added to serum-free DMEM containing 5% fatty-acid-free BSA to obtain a 250 µM palmitic acid solution, and the resulting diluted solution was used for the cell treatments [29,30]. All PA treatments were performed in serum-free medium for 24 h. Control cells received an equivalent volume of BSA vehicle.

Cell viability assay. Cells were seeded in 96-well plates at 1.0 × 10^4^ cells/well and incubated overnight. Cells were then treated with various concentrations of compounds (0.1–100 μM) for 24 h. The medium was then replaced with fresh medium containing 10% CCK-8 (Macklin, Shanghai, China). After incubation for 4 h at 37 °C, optical density (OD) values were measured at 450 nm using a microplate reader. Cell viability was calculated as percentage of the control (untreated) group. Only concentrations showing ≥90% viability were used for subsequent bioactivity assays.

Mitochondrial membrane potential (MMP) assay. The treated cells were then used for JC-1 fluorescent probe staining (JC-1, 500×, J675024, Macklin, Shanghai, China) according to the manufacturer’s protocols. Briefly, cells in 96-well plates were incubated with JC-1 staining solution (5 μg/mL) at 37 °C for 30 min in the dark. After incubation, cells were washed twice with PBS. Fluorescence was measured using a multi-mode microplate reader at 485/530 nm (excitation/emission, green, for monomeric JC-1) and 530/580 nm (excitation/emission, red, for aggregated JC-1). MMP was expressed as the ratio of red to green fluorescence intensity. The JC-1 ratio in each sample was normalized to the control (untreated) group.

Intracellular ROS measurement. AML-12 cells were cultured in 96-well plates at 1.0 × 10^4^ cells/well. After reaching 70–80% confluency, the medium was discarded, and the cells were washed with PBS for 3 times. The treated cells were incubated with 10 μM DCFH-DA fluorescent probe (CA1410, Solarbio, Beijing, China) for 20 min at 37 °C, following the manufacturer’s protocols. Then, cells were washed again with PBS for 3 times, and stained with 1 μg/mL Hoechst 33342 for 10 min. Fluorescence was measured using a microplate reader (Ex/Em: 485/528 nm for ROS; 352/461 nm for Hoechst). The average ROS fluorescence intensity per cell was calculated by dividing the total ROS fluorescence intensity by the total Hoechst fluorescence intensity in the same well. For imaging, cells were observed and images were collected through a fluorescence microscope (Nikon Corporation, Tokyo, Japan).

ATP production assay. Cellular ATP levels were measured using an ATP assay kit (Beyotime, Shanghai, China) according to the manufacturer’s instructions. Briefly, cells cultured in 96-well plates were lysed with ATP-releasing buffer provided by the kit. An aliquot (20 μL) of the lysate was transferred to a black opaque 96-well plate and mixed with 100 μL of ATP detection reagent. Luminescence intensity was measured using a microplate reader. Meanwhile, another aliquot of the same lysate was used to determine protein concentration using the BCA protein assay kit (Thermo Fisher Scientific, Waltham, MA, USA) to normalize ATP content. ATP levels were expressed as nmol/mg protein and presented as fold change relative to the control group.

#### 3.2.4. Protective Effects of Compounds Against UVB-Induced Damage in HaCaT Cells

HaCaT cells were cultured in DMEM with 10% FBS at 37 °C in 5% CO_2_. Cells were seeded into 12-well plates at 3.0 × 10^5^ cells per well and incubated overnight. After reaching 40–60% confluence, cells were pre-incubated for 4 h with either fresh medium (blank control and model groups), 10 μM compounds (sample group), or 20 μg/mL vitamin C (positive control group). The blank control group received no UVB irradiation, while all other groups were exposed to UVB irradiation (30 or 200 mJ/cm^2^) in HBSS using a UVB lamp (Philips, TL 20W/01RS, λ = 311 nm). After UVB exposure, cells were post-incubated overnight with the corresponding treatments.

For the assessment of mitochondrial membrane potential (MMP), cells were stained with JC-1 dye (5 μg/mL) at 37 °C for 30 min in the dark. After incubation, cells were washed twice with PBS, trypsinized, and resuspended in staining buffer. Fluorescence was analyzed using flow cytometry (BD FACSCalibur) with excitation at 488 nm, detecting green fluorescence (monomeric JC-1, FL1 channel) and red fluorescence (aggregated JC-1, FL2 channel). MMP was expressed as the ratio of red to green fluorescence intensity, normalized to the blank control group.

Intracellular ROS levels were assessed using the DCFH-DA probe. After probe incubation (10 μM, 30 min at 37 °C), cells were washed, trypsinized, and analyzed by flow cytometry (488 nm excitation, FITC channel) to measure geometric mean fluorescence intensity (GE mean). ROS levels were expressed as fold change relative to the blank control group. All experiments were performed in three independent biological replicates (*n* = 3). Data are presented as mean ± SD. Statistical significance was determined using one-way ANOVA with Student’s *t*-test.

#### 3.2.5. Anti-Glycation Effects of Compound **4** on Glucose-Induced Injury in HDF Cells

Human dermal fibroblasts (HDF) were cultured in DMEM with 10% FBS at 37 °C in 5% CO_2_. HDF were seeded into 6-well plates at 2.0 × 10^5^ cells per well and incubated overnight. Cells were then treated for 24 h with either complete medium (blank control), 125 mM glucose (model group), or 125 mM glucose combined with 10 μM compound **4** (sample group). After incubation, the culture supernatants were collected and centrifuged at 1000× *g* for 20 min. The levels of advanced glycation end products (AGEs) in the supernatants were measured using an AGE-specific ELISA kit according to the manufacturer’s instructions. Absorbance was read at 450 nm using a microplate reader. AGE levels were expressed as percentage of the model group. All experiments were performed in three independent biological replicates (*n* = 3). Data are presented as mean ± SD. Statistical significance was determined using one-way ANOVA with Student’s *t*-test.

#### 3.2.6. Statistical Analysis

All data are presented as mean ± standard deviation (SD) from at least three independent biological replicates, each performed with three technical replicates (*n* = 3). Statistical analyses were performed using GraphPad Prism (version 9.0). One-way ANOVA and Student’s *t*-test were employed in this research for the analysis of statistics. A *p*-value of < 0.05 was considered statistically significant. Significance levels are denoted as follows: * *p* < 0.05, ** *p* < 0.01, *** *p* < 0.001, **** *p* < 0.0001. All statistical tests, sample sizes, and replicate numbers are specified in the corresponding figure legends.

### 3.3. Molecular Dynamics (MD) Simulations

To investigate the structural and dynamical effects of compound **2** on a lipotoxic mitochondrial environment, molecular dynamics (MD) simulations were performed for compound **2** using GROMACS (version 2025.1) [31]. An asymmetric-like complex lipid mixture mimicking the mitochondrial membrane was constructed using CHARMM-GUI [32,33,34,35,36,37]. The membrane composition consisted of 45% POPC (90 lipids), 34% POPE (68 lipids), 11% TLCL2/Cardiolipin (22 lipids), and 10% PAL/Palmitic Acid (20 lipids). Two systems were prepared: a control system containing 54,335 total atoms, which included the lipids, TIP3P water, and neutralizing ions, and an experimental system of 59,996 total atoms, in which five molecules of compound **2** were randomly placed in the aqueous phase above the membrane leaflets (three on the upper leaflet, two on the lower). The control represents a “sick” or leaky membrane state characterized by PAL-induced rigid, dehydrated gel-phase domains, whereas the experimental system models the “rescued” state.

Compound **2** was parameterized using the CHARMM General Force Field (CGenFF). The resulting topology and parameter files yielded minimal penalty scores, validating the direct use of the CGenFF-assigned parameters for the simulations (https://cgenff.com/) [38,39,40].

Both the control and experimental systems underwent minimization and equilibration utilizing standard CHARMM-GUI protocols [32,34]. Energy minimization was completed in a single step, followed by a six-step equilibration process. System temperature was maintained at 303.15 K using the v-rescale thermostat [41] with a coupling time constant of 1.0 ps, applied separately to the membrane and solvent groups. Pressure was maintained at 1.0 bar using the C-rescale barostat [42] with a semi-isotropic coupling scheme and a time constant of 5.0 ps, ensuring proper calculation of the lipid bilayer dynamics. Long-range electrostatic interactions were evaluated using the Particle Mesh Ewald (PME) method [43] with a 1.2 nm cut-off. Van der Waals interactions were calculated using a force-switch modifier tapering from 1.0 nm to a cut-off of 1.2 nm. The LINCS algorithm [44] was employed to constrain all bonds involving hydrogen atoms, allowing for an integration time step of 2 fs. Production simulations were conducted for 1 μs (1000 ns) per system, with coordinates saved every 0.1 ns to yield 10,000 frames per trajectory. Trajectory analysis was executed using the MDAnalysis Python (version 2.7.0) [45], supplemented by NumPy (version 1.26.4) [46], SciPy (version 1.14.1) [47], and Pandas (version 2.2.2) [48] for array operations and statistical calculations, alongside Matplotlib (version 3.9.2) [49] for data visualization. Visual inspections and rendering were performed using VMD 2.0 [50].

#### 3.3.1. Equilibration and Global Membrane Properties

To ensure the system reached a stable thermodynamic state before analytical measurements, system equilibration was evaluated by calculating the root-mean-square deviation (RMSD) of the lipid headgroups (phosphorus atoms). A 50 ns sliding window was applied across the trajectory to systematically define the equilibration cutoff. A linear regression was performed within this window, and the system was deemed fully equilibrated once the absolute slope fell below a defined threshold of 0.001, signifying the plateau.

Global structural expansion, condensation, or thinning induced by the compounds was quantified through Area Per Lipid (APL) and membrane thickness measurements. The global APL was calculated by extracting the simulated box dimensions at each frame, dividing the total XY planar area by the number of lipids per leaflet:

$$APL=\frac{L_{x}\times L_{y}}{N_{lipids/leaflet}}$$

Membrane thickness was defined as the absolute *Z*-axis distance between the center of mass (COM) of the upper and lower leaflet phosphorus headgroups.

#### 3.3.2. Compound Localization and Orientation

The spatial distribution and thermal noise of the compounds within the lipotoxic membrane environment were mapped by calculating the absolute distance to the membrane center. This was achieved by tracking the Z-coordinate of the compound’s center of mass relative to the membrane’s center of mass. To filter thermal noise, the depth trajectories were smoothed using a 5 ns (50-frame) moving average.

To visualize where the compounds partition relative to the internal architecture of the lipid bilayer, a 1D partial mass density profile (*ρ*_m_ (*z*)) was computed along the *Z*-axis. The box was divided into 150 bins, and the Z-coordinates of specific structural proxies (compounds, bulk water, lipid headgroups, glycerol backbone, and upper tails) were histogrammed. Counts were weighted by exact atomic masses and converted to physical density (kg/m^3^) using the volume of each bin slice.

The specific spatial alignment of the inserted compound relative to the lipid tails was determined by measuring the tilt angle. This angle (*θ*) was measured using an internal molecular axis defined by atoms H35 and H47, relative to the local membrane normal $\hat{n}$:

$$\theta =arccos\left(\hat{v}\cdot \hat{n}\right)$$

Additionally, the inherent polarity and charge distribution of the compound were evaluated by calculating the total dipole moment vector (μ) relative to the compound’s center of mass (*r_com_*), utilizing the partial charges assigned by the CGenFF force field:

$$\vec{\mu }=\sum q_{i}\left(\vec{r_{i}}-\vec{r_{com}}\right)$$

#### 3.3.3. Compound–Lipid Interactions

Specific polar interactions anchoring the compound to the lipid headgroups or glycerol backbone were dynamically mapped using strict geometric hydrogen bonding criteria: a donor-to-acceptor distance cutoff of 3.5 Å and a donor-hydrogen-acceptor angle cutoff of 150°. The broad non-covalent binding footprint of the compound within the acyl core was quantified by calculating the total number of bound lipids per compound, applying a 6.0 Å radial cutoff around the entire compound molecule.

To determine if the compound actively targets specific lipids or interacts by random spatial chance, a Preferential Binding Enrichment Factor was calculated for both H-bonds and Van der Waals contacts. This is defined as the ratio of the interaction fraction to the molar composition fraction of the lipid in the bulk membrane, where a score greater than 1.0 indicates active preference:

$$Enrichment=\frac{N_{bound\_lipid\_type}/N_{total\_interactions}}{N_{lipid\_type}/N_{total\_lipids}}$$

#### 3.3.4. Structural Disruption Metrics

Changes in the rigidity and structural organization of the lipid acyl tails were quantified vectorially via deuterium order parameters (*S_cd_*) for the carbon-hydrogen bonds along the POPC, POPE, and TLCL2 tails:

$$S_{cd}\;=\;\frac{1}{2}\;\langle 3\;{cos}^{2}\theta -1\rangle$$

To evaluate local disruption, the membrane was dynamically segmented into “bound” lipids (within 6.0 Å of the compound) and “bulk” lipids (no contact) per frame, and the S*_cd_* values were averaged separately.

Physical splaying and expansion of the lipids immediately surrounding the compounds were measured via local APL. A 2D Voronoi tessellation was applied to the XY planar coordinates of the lipid phosphorus atoms. A 3 × 3 periodic tiling was implemented to eliminate boundary artifacts before calculating polygon areas, which were then distributed into bound (<12.0 Å from compound COM) and bulk sets.

#### 3.3.5. Electrostatic Profile

The effect of the polar compounds on the deep thermodynamic electrostatic environment of the lipotoxic membrane was evaluated by tracking the Z-component projection of the compound’s dipole moment (*μ_z_*) continuously over the production trajectory:

$$\mu_{z}=\sum q_{i}\left(z_{i}-z_{com}\right)$$

The spatial electrostatic potential (*ϕ*(*z*)) across the entire system was mapped by generating a 1D *Z*-axis charge density (*ρ*(*z*)) from binned atomic partial charges, and double-integrating the Poisson equation:

$$\phi \left(z\right)=-\int \left(\int \frac{\rho \left(z\right)}{\epsilon_{0}}dz\right)dz$$

Boundary conditions were calibrated by centering the potential to 0 Volts in the bulk aqueous phase to accurately isolate the internal membrane dipole potential.

## 4. Conclusions

*Aspergillus* species are well known for producing tryptoquivaline and cytochalasin types of compounds. In our current research, two new tryptoquivaline derivatives (**1**−**2**), one new cytochalasin derivative (**3**), and methyl (*S*)-2-(2,5-dihydroxyphenyl)-2-methoxyacetate (**4**) were isolated from a hydrothermal vent crab-associated fungal strain *A. clavatus* C2WU. And we proposed a hypothetical biosynthetic pathway for compounds **1** and **2**. Compound **4** showed a relatively strong ability to scavenge free radicals. Compound **4** (10 μM) reduced UVB-induced ROS levels in HaCaT cells and decreased glucose-induced AGE formation in HDF cells, exhibiting a relatively strong free radical scavenging activity. In conclusion, compound **4** demonstrates promising cytoprotective effects against oxidative and glycative stress, likely mediated through its antioxidant capacity. Further studies are warranted to elucidate its molecular mechanism and evaluate its in vivo efficacy for skin anti-aging applications.

Compounds **1**, **2**, and **5** significantly restored the UVB-induced reduction in the JC-1 red/green fluorescence ratio and decreased intracellular ROS levels, indicating that they protect skin cells from mitochondrial damage and oxidative stress, thereby exerting anti-photoaging effects.

In addition, we demonstrated that compound **2** protects against PA-induced mitochondrial dysfunction in AML-12 cells. Specifically, compound **2** restored mitochondrial membrane potential (MMP) and enhanced ATP production, albeit with slightly lower efficacy than the positive control acadesine in the MMP assay. Notably, although compound **2** effectively reduced intracellular ROS levels, it exhibited no direct antioxidant activity in cell-free systems. This suggests that its ROS-lowering effect is likely secondary to mitochondrial protection rather than direct radical scavenging. By preserving mitochondrial function, compound **2** may limit mitochondrial ROS release, thereby breaking the vicious cycle of oxidative stress and mitochondrial damage. Additionally, our simulations support a cardiolipin-associated membrane-modulation mechanism, suggesting that the functional rescue may originate at the membrane interface, where compound **2** may utilize a selective multi-point anchor to interact with cardiolipin. By physically melting rigid lipotoxic domains, the compound reconstitutes the internal dipole architecture required to maintain the mitochondrial membrane potential. These findings highlight compound **2** as a promising candidate for mitigating high-fat-induced hepatic injury, though its upstream molecular targets remain to be elucidated.

Due to the limited number of compounds and their structural diversity, a comprehensive structure–activity relationship (SAR) cannot be established at this stage. However, the observation that quinazoline–indole alkaloids (**1** and **2**) exhibit mitochondrial protection suggests that the quinazoline core may be associated with mitochondrial preservation. In contrast, compound **4**, a simple phenolic ester lacking the quinazoline moiety, showed strong ROS scavenging but negligible mitochondrial protection, indicating that direct radical scavenging and mitochondrial preservation are distinct activities. Future SAR studies through chemical derivatisation and analogue synthesis are required to validate these preliminary observations.

While the in vitro results are promising, several challenges must be addressed before translational development. First, the pharmacokinetic properties of compounds **1**–**5**, including bioavailability, metabolic stability, and tissue distribution, remain unexplored. Second, for skin anti-aging applications, topical delivery would require formulation optimisation and skin penetration studies. Third, for hepatic protection, systemic administration would necessitate evaluation of hepatotoxicity and off-target effects. Future work will include in vivo efficacy studies in appropriate animal models (e.g., UVB-induced skin photodamage in mice and high-fat diet-induced hepatic steatosis models), as well as pharmacokinetic profiling and formulation development.

## Figures and Tables

**Table 1:** Summary of compounds isolated from *A. clavatus* C2WU.

Compound No.	Name	Type	Purity
1	Tryptoquivaline Z_1_	Quinazoline-containing indole alkaloid	87%
2	Clavutoine V	Quinazoline-containing indole alkaloid	89%
3	Cytochalasin Z_29_	Cytochalasan alkaloid	86%
4	Methyl (*S*)-2-(2,5-dihydroxyphenyl)-2-methoxyacetate	Phenolic ester	88%
5	Arthriniumnin A	Known compound	96%

**Table 2:** ^1^H NMR (600 MHz) and ^13^C NMR (150 MHz) Data of **1**–**2** in DMSO-*d*_6_.

No.	1		2	
	*δ*_C_ (ppm), Type	*δ*_H_ (ppm), (Mult., *J* in Hz)	*δ*_C_ (ppm), Type	*δ*_H_ (ppm), (Mult., *J* in Hz)
2	86.7, CH	5.24, s	83.0, CH	5.32, s
3	84.7, C		76.5, C	
3-OH				5.46, s
4	135.1, C		139.1, C	
5	126.5, CH	8.04, d (7.7)	125.3, CH	7.32, m
6	125.8, CH	7.32, t (7.4)	124.9, CH	7.09, t (7.3)
7	132.0, CH	7.50, m	130.1, CH	7.29, m
8	115.3, CH	7.47, m	115.6, CH	7.32, m
9	137.6, C		138.0, C	
10	171.1, C		175.5, C	
11	71.3, C		61.0, CH	3.78, d (7.3)
12				
13	34.7, CH_2_	a 3.32, m b 2.99, dd (13.8, 9.6)	36.1, CH_2_	a 2.97, dd (15.3, 9.0) b 2.60, dd (15.3, 2.7)
14	55.3, CH	6.16, t (9.6)	57.9, CH	5.19, d (8.0)
15	170.7, C		170.0, C	
16				
17	161.0, C		160.4, C	
18	120.1, C		122.1, C	
19	126.9, CH	8.16, d (7.9)	126.7, CH	8.13, d (7.8)
20	128.7, CH	7.59, t (7.6)	127.7, CH	7.54, t (7.8)
21	136.3, CH	7.86, t (7.6)	135.2, CH	7.83, t (7.0)
22	127.9, CH	7.66, m	127.7, CH	7.66, d (8.1)
23	146.7, C		148.3, C	
24				
25	155.3, C		149.0, CH	8.41, s
26	17.5, CH_3_	1.33, s	18.3, CH_3_	1.35, d (7.0)
27	23.4, CH_3_	1.30, s		
28	76.8, CH	6.11, d (8.4)		
29	32.9, CH	2.62, m		
30	18.8, CH_3_	1.24, d (6.6)		
31	18.3, CH_3_	1.03, d (6.6)		
32	166.4, C			
33	129.1, C			
34	130.4, CH	7.98, d (7.1)		
35	129.6, CH	7.52, m		
36	134.5, CH	7.66, m		
37	129.6, CH	7.52, m		
38	129.9, CH	7.98, d (7.1)		
15-OCH_3_			53.3, CH_3_	3.62, s

**Table 3:** ^1^H NMR (600 MHz) and ^13^C NMR (150 MHz) Data of **3** in DMSO-*d*_6_.

No.	3	
	*δ*_C_ (ppm), Type	*δ*_H_ (ppm), (Mult., *J* in Hz)
1	170.1, C	
2		8.13, s
3	56.0, CH	2.94, m
4	49.3, CH	2.67, t (3.7)
5	34.3, CH	2.42, br
6	141.4, C	-
7	122.9, CH	5.17, s
8	48.1, CH	2.80, m
9	89.6, C	-
10	43.2, CH_2_	2.84, d (6.8)
11	13.7, CH_3_	0.60, d (7.3)
12	20.2, CH_3_	1.62, s
13	128.2, CH	5.53, dd (15.0, 11.0)
14	133.1, CH	5.29, dd (15.0, 3.6)
15	37.5, CH_2_	2.27, q (11.1) 1.92, m
16	38.4, CH	3.59, d (2.2)
17	217.6, C	-
18	77.6, C	-
19	122.1, CH	5.35, d (12.4)
20	139.9, CH	6.45, d (12.4)
21		
22	150.1, C	-
23	19.8, CH_3_	0.94, d (6.9)
24	28.0, CH_3_	1.28, s
1′	138.4, C	-
2′	128.9, CH	7.14, m
3′	130.1, CH	7.26, t (7.3)
4’	127.0, CH	7.17, m
5’	130.1, CH	7.26, t (7.3)
6’	128.9, CH	7.14, m
18-OH		5.83, s

**Table 4:** ^1^H NMR (600 MHz) and ^13^C NMR (150 MHz) Data of **4** in DMSO-*d*_6_.

No.	4	
	*δ*_C_ (ppm), Type	*δ*_H_ (ppm), (Mult., *J* in Hz)
1	171.7, C	
2	76.1, CH	5.01, d (1.9)
3	123.7, C	
4	148.1, C	
5	116.7, CH	6.64, d (8.6)
6	116.7, CH	6.52, dd (8.6, 2.3)
7	150.3, C	
8	114.5, CH	6.55, d (2.3)
1-OCH_3_	52.2, CH_3_	3.57, s
2-OCH_3_	57.3, CH_3_	3.22, s

## Data Availability

All relevant data supporting the findings of this study are contained within the article and its Supplementary Materials. Raw data (NMR, MS and IR spectra) and MD trajectories are available from the corresponding author upon reasonable request.

## Supplementary Materials

The following supporting information can be downloaded at https://www.mdpi.com/article/10.3390/md24080268/s1. Figures S1–S31: NMR, IR and MS spectra of 1–4; Figures S32−S36: Membrane equilibration, structural modulation, compound orientation, membrane fluidization, and electrostatic potential; Figure S37: DPPH radical scavenging activity of compound 4; Tables S1–S4: Key 2D NMR correlations for compounds 1–4; Tables S5–S6: Highest-occupancy intermolecular hydrogen bond interactions; Tables S7–S9: MD simulation parameters, optimized conformers and Z-matrixes.

## Abbreviations

The following abbreviations are used in this manuscript:

NMR	Nuclear Magnetic Resonance
ECD	Electronic Circular Dichroism
MMP	mitochondrial membrane potential
ROS	Reactive Oxygen Species
AGEs	Advanced Glycation End Products
HDF	Human Dermal Fibroblast
HPLC	High-Performance Liquid Chromatography
