# Supplementary material for: New Alkaloids from the Hydrothermal Vent-Derived Fungus Aspergillus clavatus C2WU and Their Mitochondrial Protective and Anti-Photoaging Effects
Source: Mar Drugs. 2026 Aug 3;24(8):268. doi: 10.3390/md24080268 (PMC13514577; doi:10.3390/md24080268)
Supplement: Supplementary file 1 [file marinedrugs-24-00268-s001.zip › marinedrugs-4385602-supplementary.pdf]

Supporting Information

New Alkaloids from the Hydrothermal Vent Derived Fungus *Aspergillus clavatus* C2WU and Their Mitochondrial Protective and Anti-Photoaging Effects

Jiayu Pan,[a,+] Chengzeng Zhou,[c,+] Jihua Wei,[*,b] David Simeunovic,[a] Qizhao Yin,[a] Mengji Zou,[a] Xiaodan Wu,[c] Zhe Feng,[b] Minjie Zhang,[b] Hu Huang,[*,b] Bin Wu[*,a]

[a] *Ocean College, Zhejiang University, Zhoushan 316000, China.*

[b] *Proya Cosmetics Co., Ltd, Hangzhou, 310012, China*

[c] *Fujian Provincial Key Laboratory of Screening for Novel Microbial Products, Fujian Institute of Microbiology, Fuzhou, 350007, China*

[c] *Analysis Center of Agrobiology and Environment Sciences, Zhejiang University, Hangzhou 310058, China.*

* Corresponding authors.

E-mail addresses: wubin@zju.edu.cn; huanghu@proya.com

+ These authors contributed equally to this work.

Table of contents

Supplementary Figures 3
Figure S1. ^{1}H NMR of compound **1** (in DMSO-d_6, 600 MHz) 3
Figure S2. ^{13}C NMR of compound **1** (in DMSO-d_6, 150 MHz). 3
Figure S3. HSQC of compound **1** (in DMSO-d_6). 4
Figure S4. COSY of compound **1** (in DMSO-d_6). 4
Figure S5. HMBC of compound **1** (in DMSO-d_6) 5
Figure S6. NOESY of compound **1** (in DMSO-d_6). 5
Figure S7. HRESIMS of compound **1**. 6
Figure S8. ^{1}H NMR of compound **2** (in DMSO-d_6, 600 MHz) 6
Figure S9. ^{13}C NMR of compound **2** (in DMSO-d_6, 150 MHz). 7
Figure S10. HSQC of compound **2** (in DMSO-d_6). 7
Figure S11. COSY of compound **2** (in DMSO-d_6) 8
Figure S12. HMBC of compound **2** (in DMSO-d_6). 8
Figure S13. NOESY of compound **2** (in DMSO-d_6) 9
Figure S14. HRESIMS of compound **2**. 9
Figure S15. ^{1}H NMR of compound **3** (in DMSO-d_6, 600 MHz). 10
Figure S16. ^{13}C NMR of compound **3** (in DMSO-d_6, 150 MHz). 10
Figure S17. HSQC of compound **3** (in DMSO-d_6). 11
Figure S18. COSY of compound **3** (in DMSO-d_6) 11
Figure S19. HMBC of compound **3** (in DMSO-d_6). 12
Figure S20. NOESY of compound **3** (in DMSO-d_6) 12
Figure S21. HRESIMS of compound **3**. 13
Figure S22. ^{1}H NMR of compound **4** (in DMSO-d_6, 600 MHz). 13
Figure S23. ^{13}C NMR of compound **4** (in DMSO-d_6, 150 MHz). 14
Figure S24. HSQC of compound **4** (in DMSO-d_6). 14
Figure S25. COSY of compound **4** (in DMSO-d_6) 15
Figure S26. HMBC of compound **4** (in DMSO-d_6). 15
Figure S27. HRESIMS of compound **4**. 16
Figure S28. IR spectrum of compound **1**. 16
Figure S29. IR spectrum of compound **2**. 16
Figure S30. IR spectrum of compound **3**. 17
Figure S31. IR spectrum of compound **4**. 17
Figure S32. Thermodynamic equilibration of the simulated membrane systems 17
Figure S33. Macroscopic structural modulation of the membrane upon compound insertion. 18
Figure S34. Orientational distribution of the compound within the lipid bilayer. 19
Figure S35. Compound-induced local and global membrane fluidization and structural disruption. 21
Figure S36. Spatial electrostatic potential profile of the simulated membrane systems. 21
Figure S37. DPPH radical scavenging activity of compound **4**. 22

Supplementary Tables 22
Table S1. Key 2D NMR correlations for compound **1**. 22
Table S2. Key 2D NMR correlations for compound **2**. 23
Table S3. Key 2D NMR correlations for compound **3**. 24
Table S4. Key 2D NMR correlations for compound **4**. 25
Table S5. Top 15 highest-occupancy intermolecular hydrogen bond interactions. 25
Table S6. Highest-occupancy intermolecular hydrogen bonds formed specifically between the compound and TLCL2........ 26
Table S7. MD simulation parameters at-a-glance. 26
Table S8. Optimized conformers and their boltzmann populations. 27
Table S9. Optimized Z-matrixes of conformers. 27

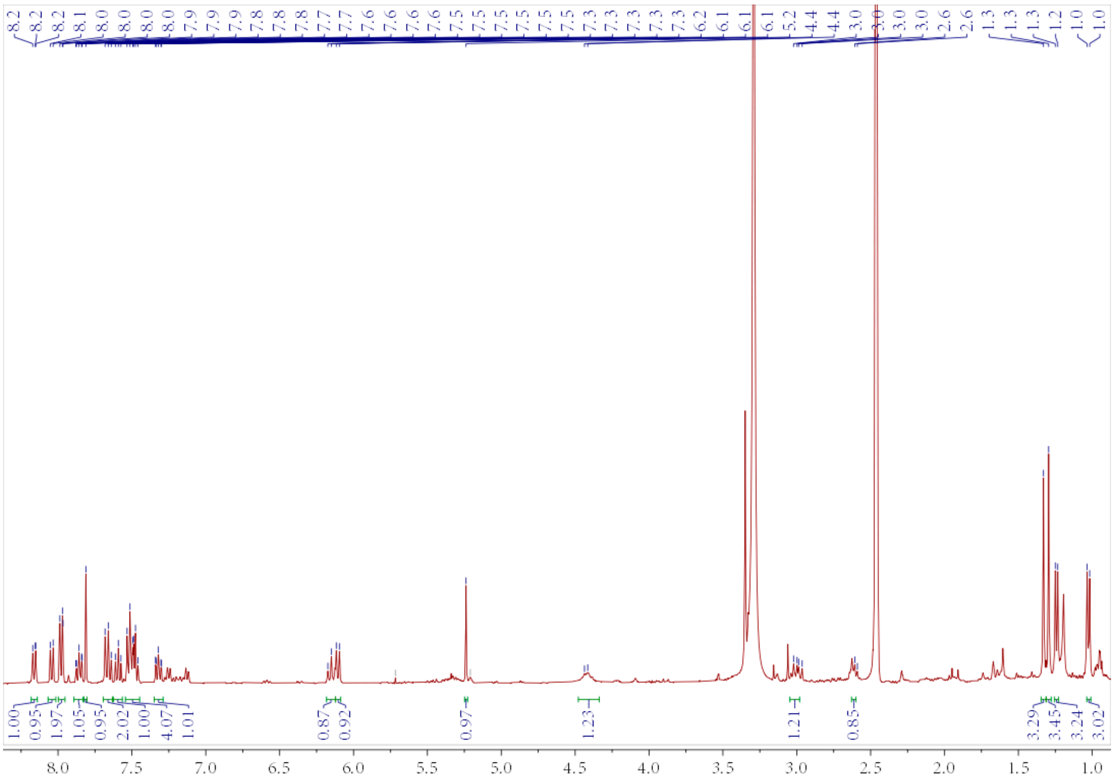

Figure S1. ^{1}H NMR of compound **1** (in DMSO-d_6, 600 MHz).

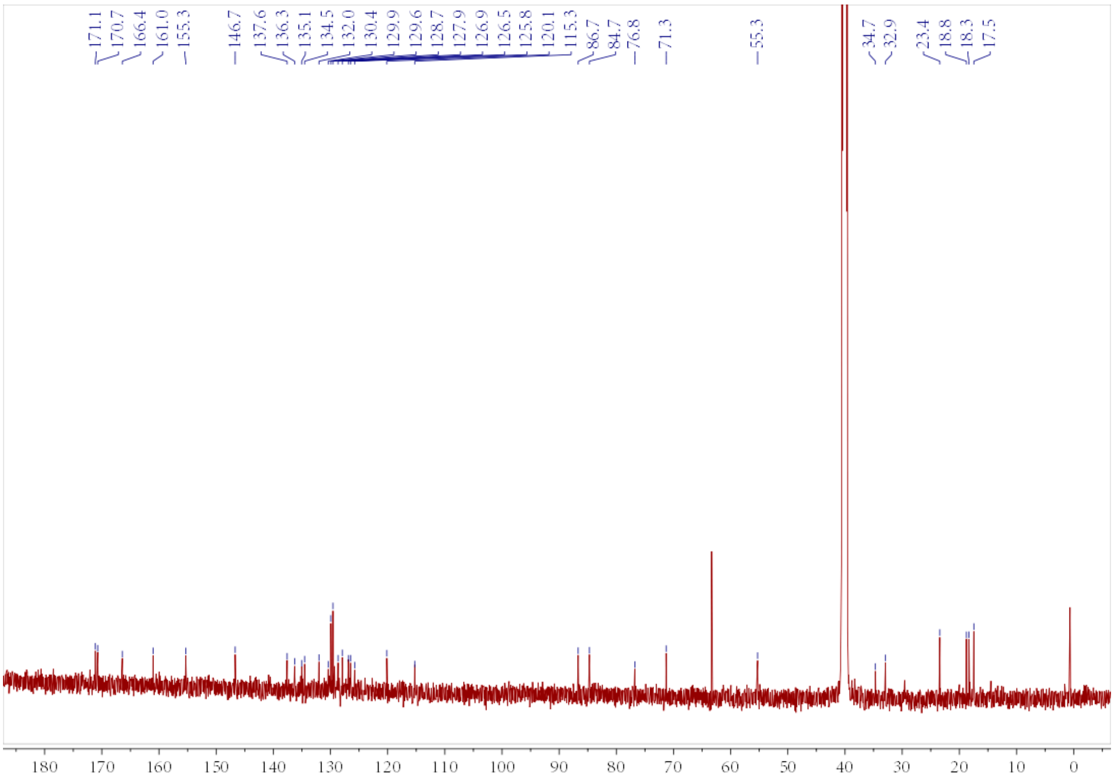

Figure S2. ^{13}C NMR of compound **1** (in DMSO-d_6, 150 MHz).

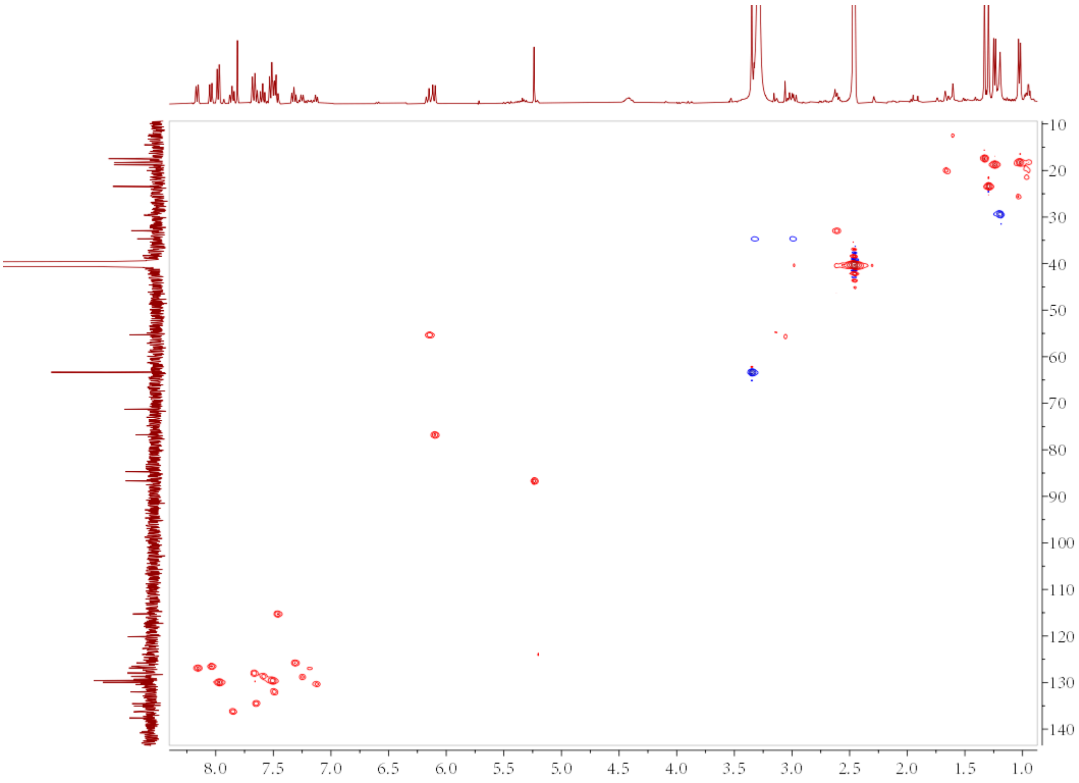

Figure S3. HSQC of compound **1** (in DMSO-d_6).

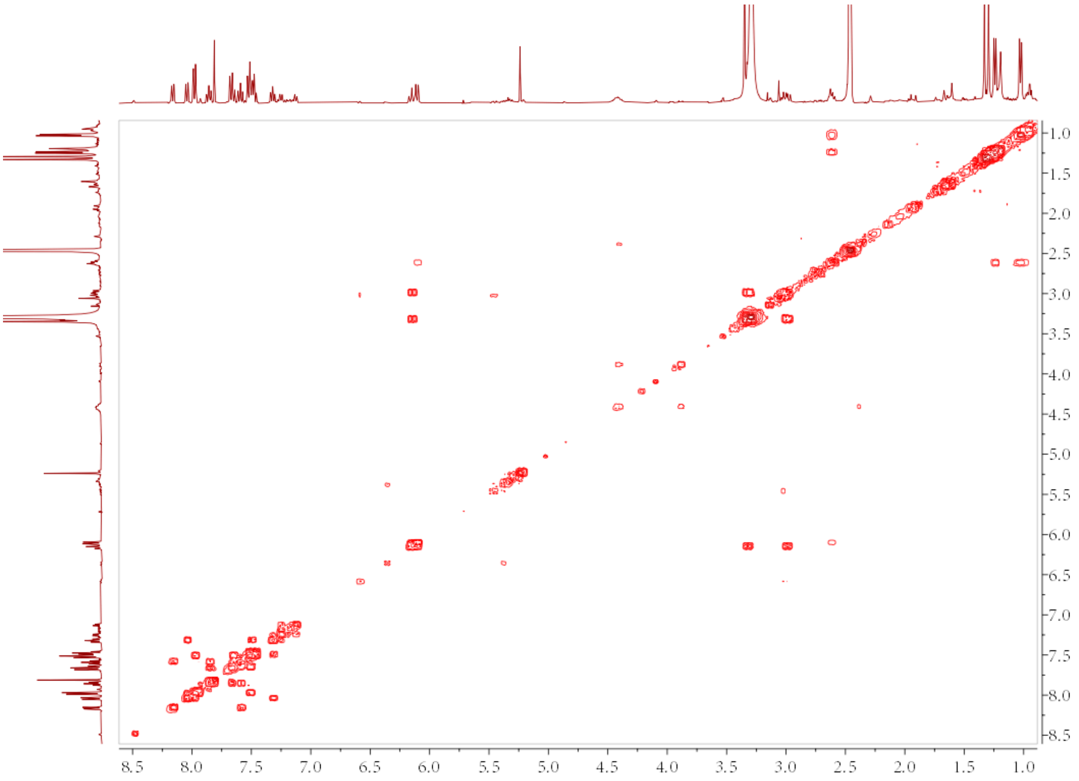

Figure S4. COSY of compound **1** (in DMSO-d_6).

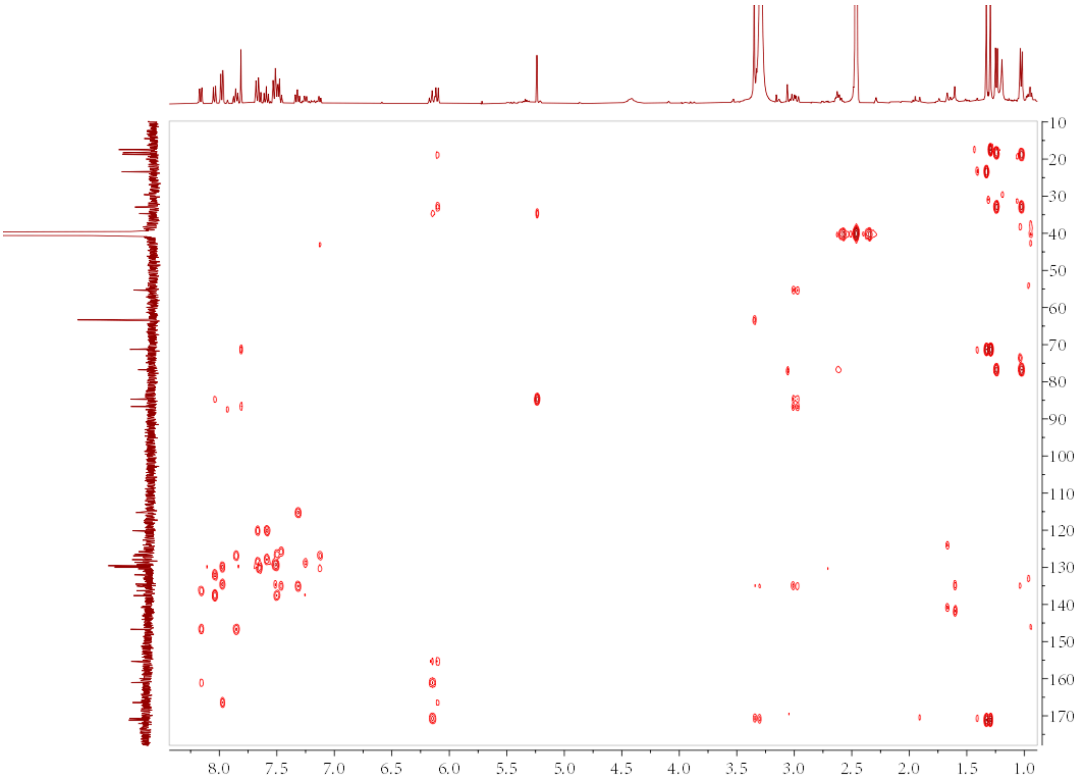

Figure S5. HMBC of compound **1** (in DMSO-d_6).

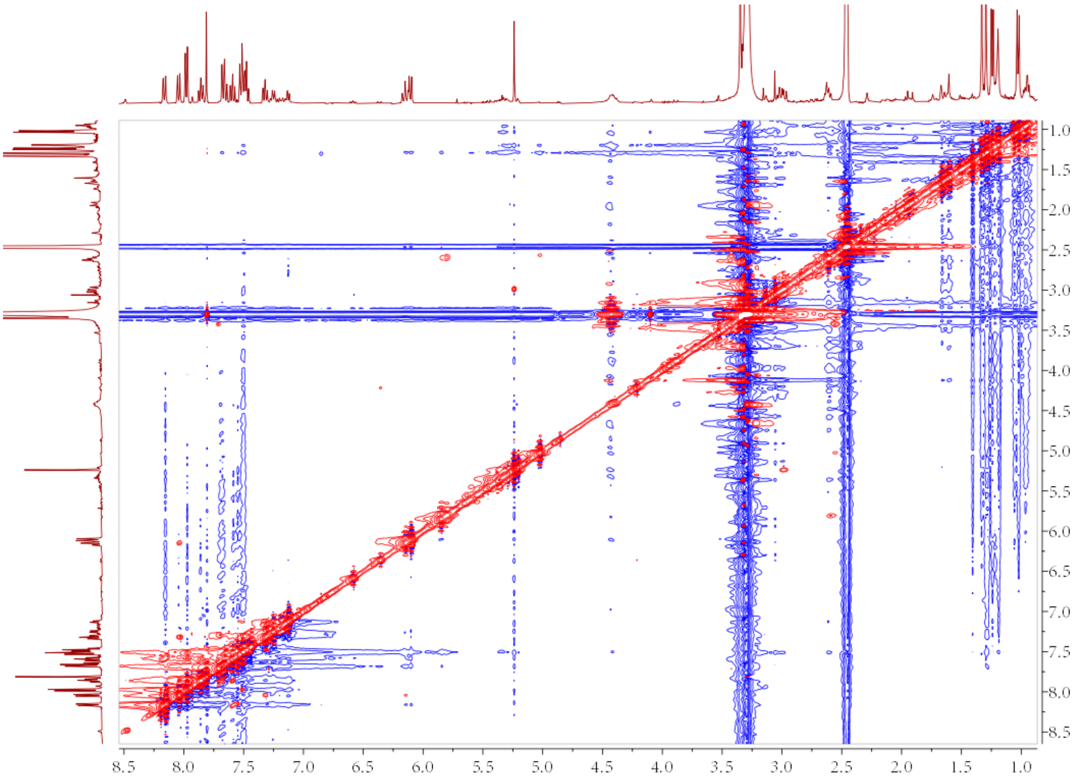

Figure S6. NOESY of compound **1** (in DMSO-d_6).

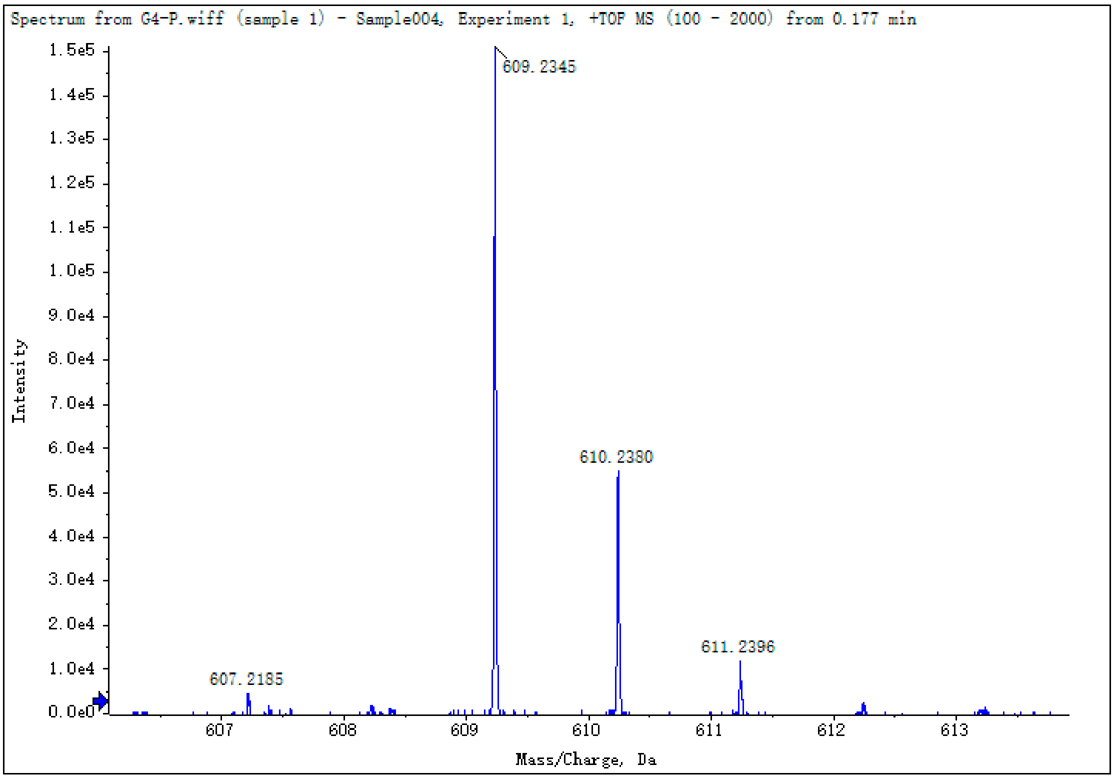

Figure S7. HRESIMS of compound **1**.

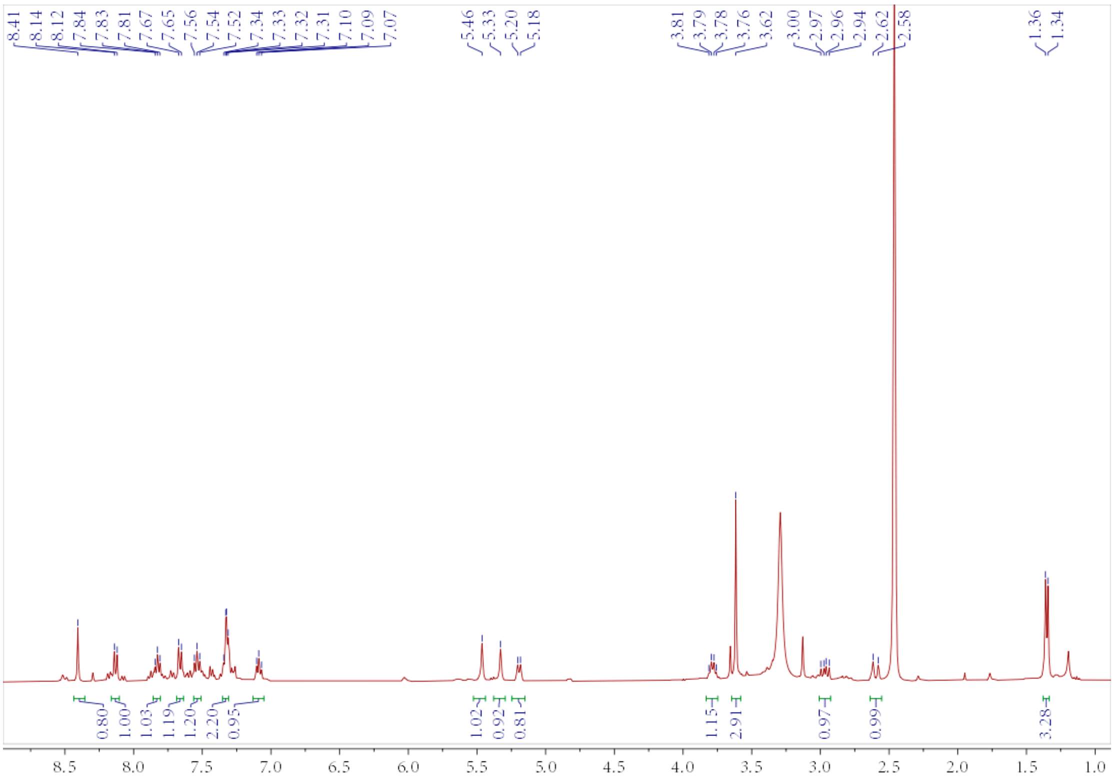

Figure S8. ^{1}H NMR of compound **2** (in DMSO-d_6, 600 MHz).

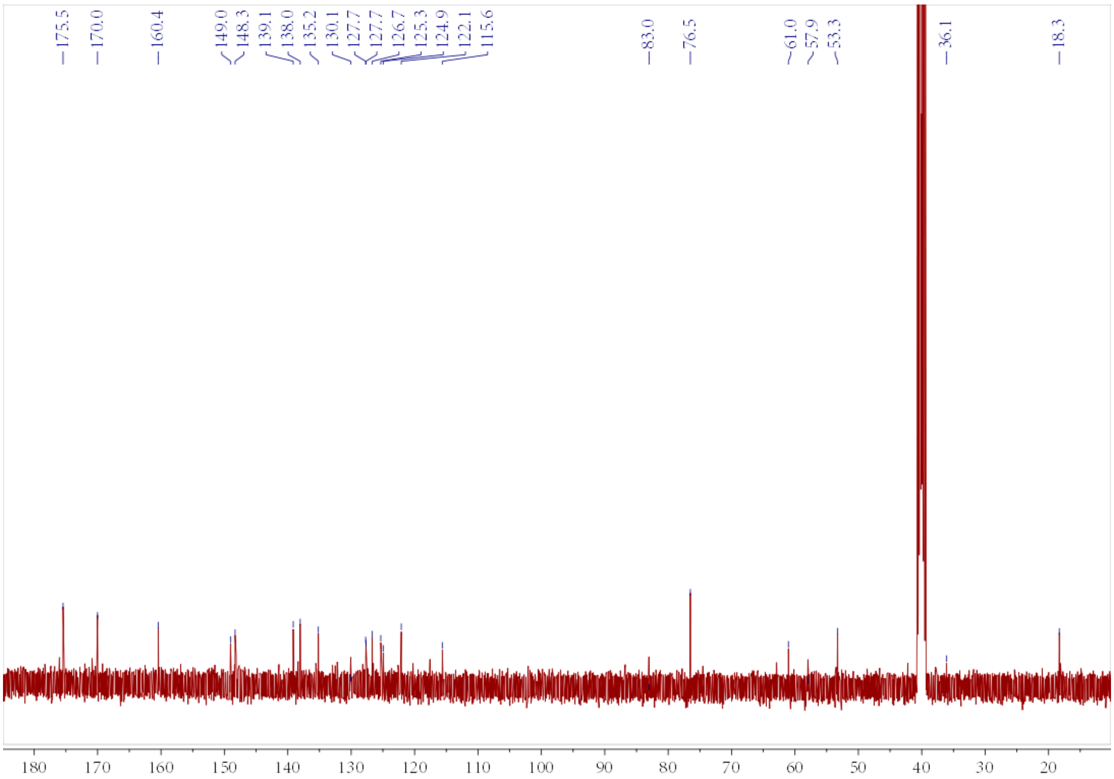

Figure S9. ^{13}C NMR of compound **2** (in DMSO-d_6, 150 MHz).

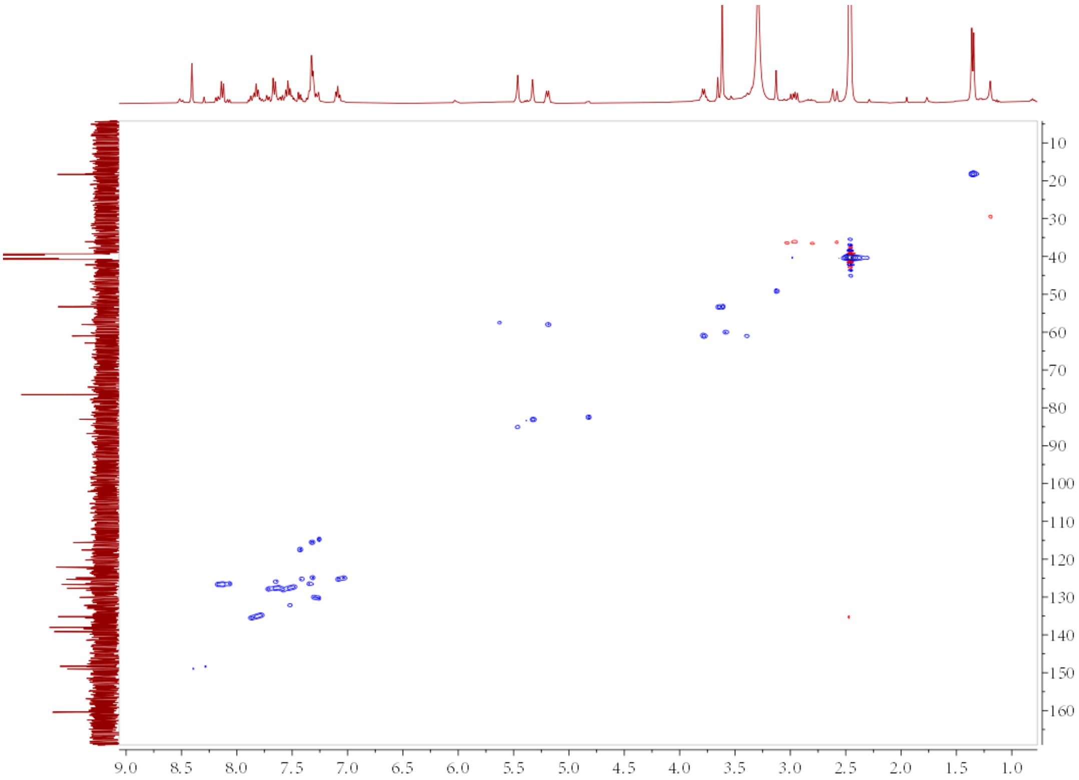

Figure S10. HSQC of compound **2** (in DMSO-d_6).

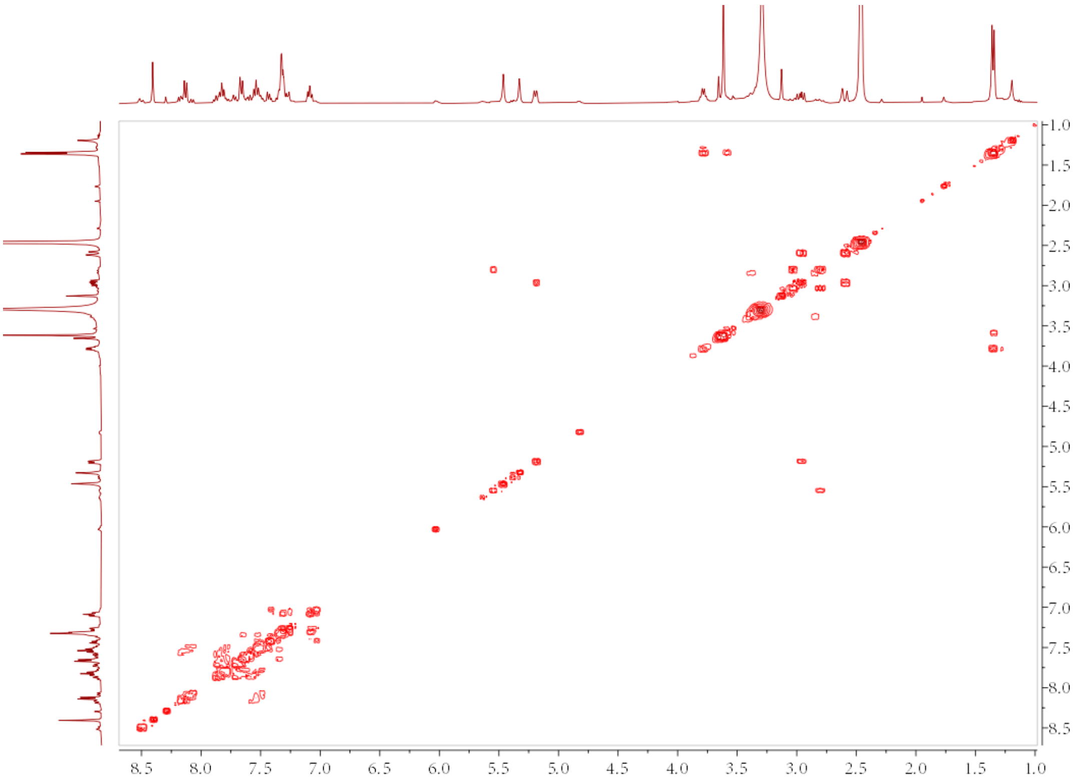

Figure S11. COSY of compound **2** (in DMSO-d_6).

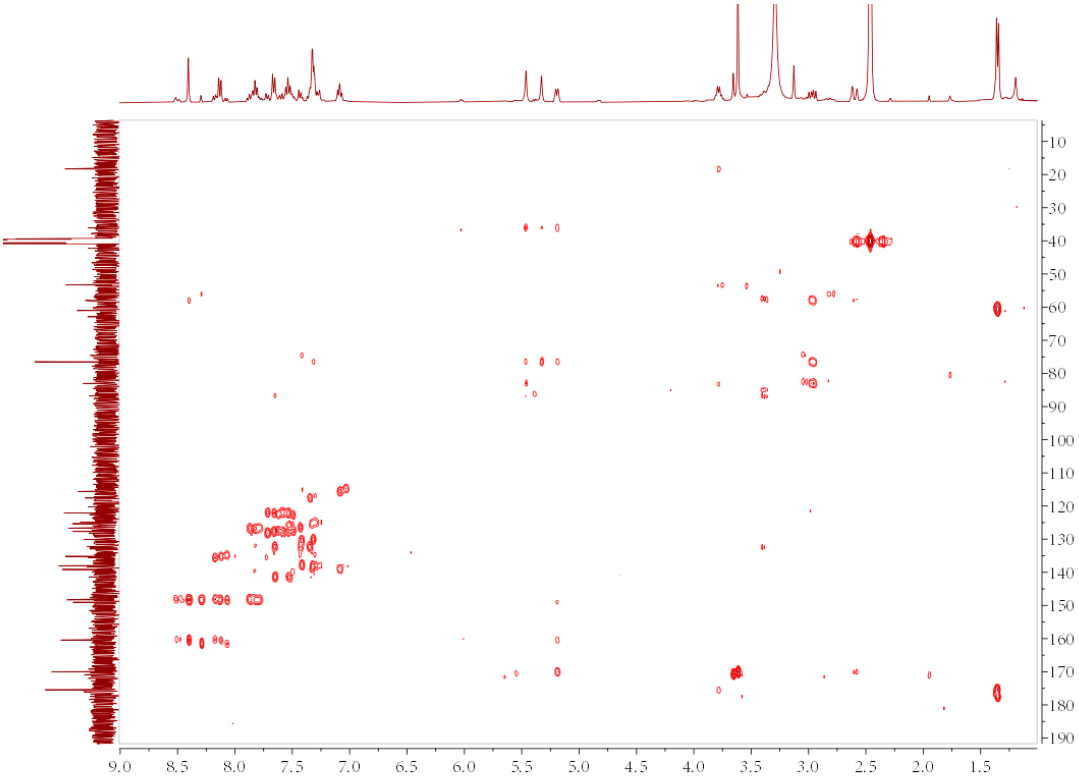

Figure S12. HMBC of compound **2** (in DMSO-d_6).

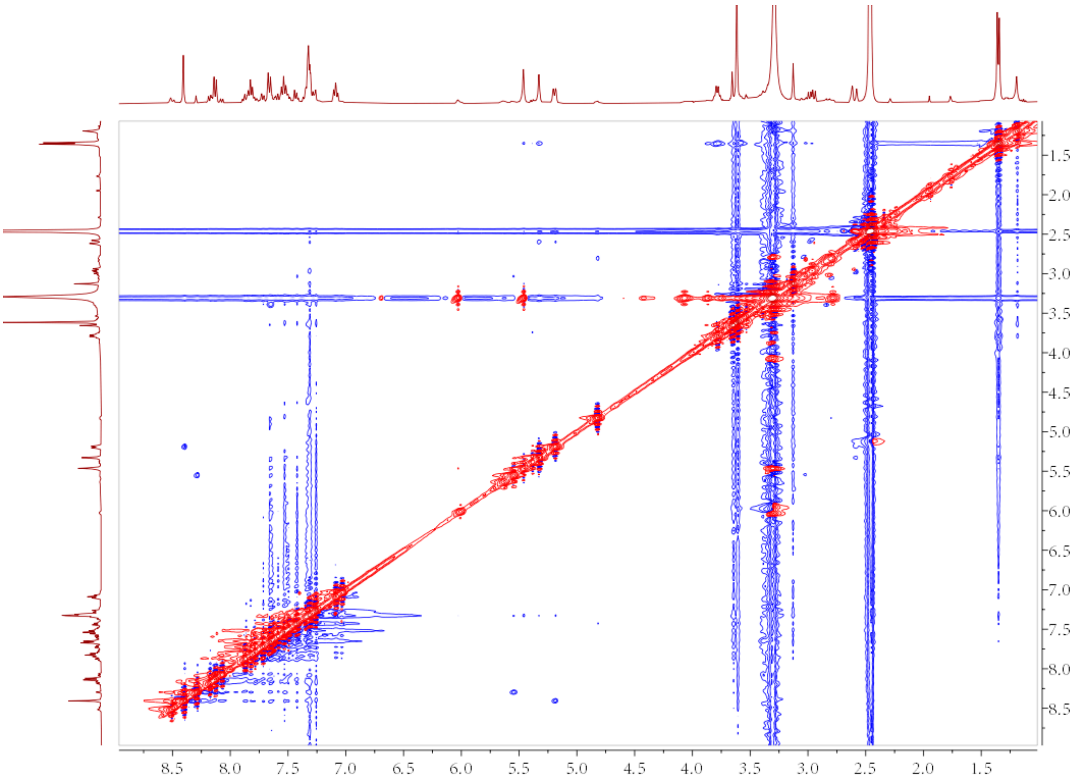

Figure S13. NOESY of compound **2** (in DMSO-d_6).

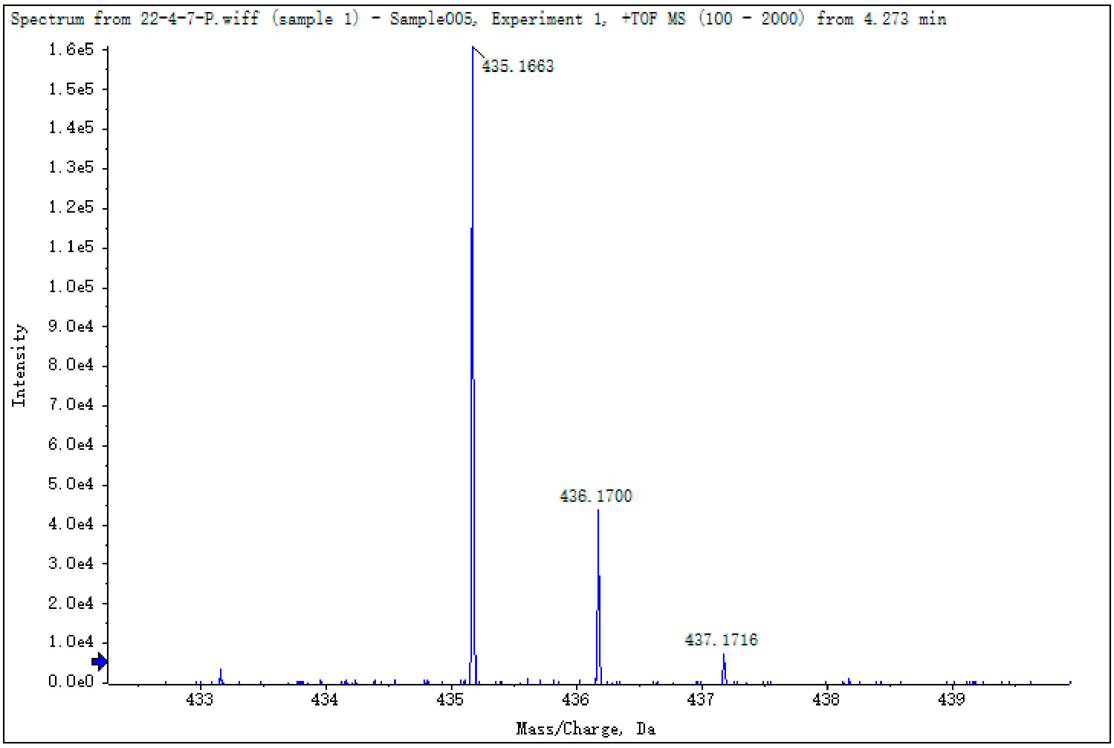

Figure S14. HRESIMS of compound **2**.

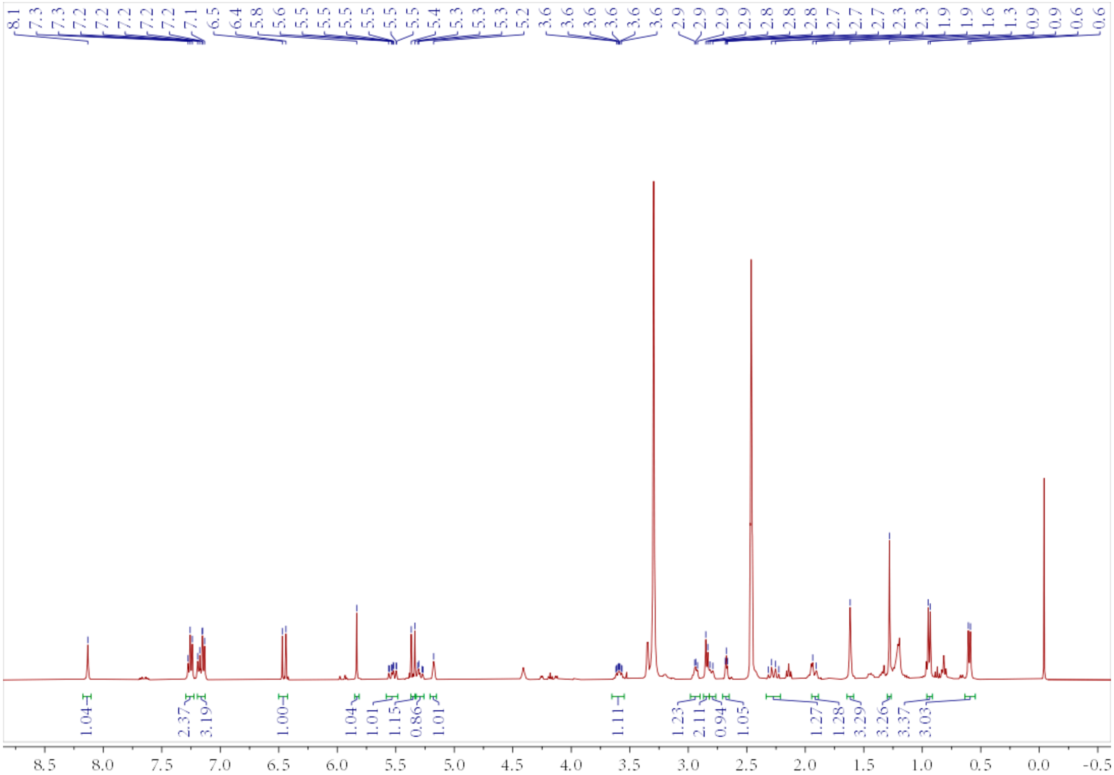

Figure S15. ^{1}H NMR of compound **3** (in DMSO-d_6, 600 MHz).

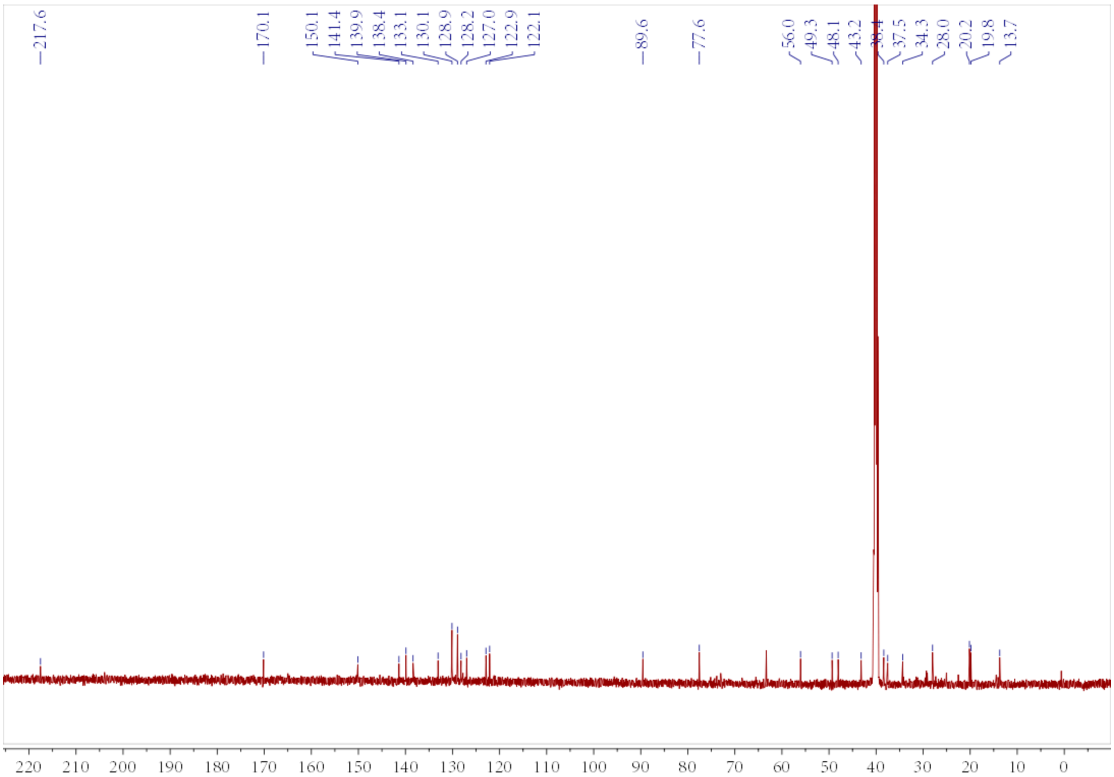

Figure S16. ^{13}C NMR of compound **3** (in DMSO-d_6, 150 MHz).

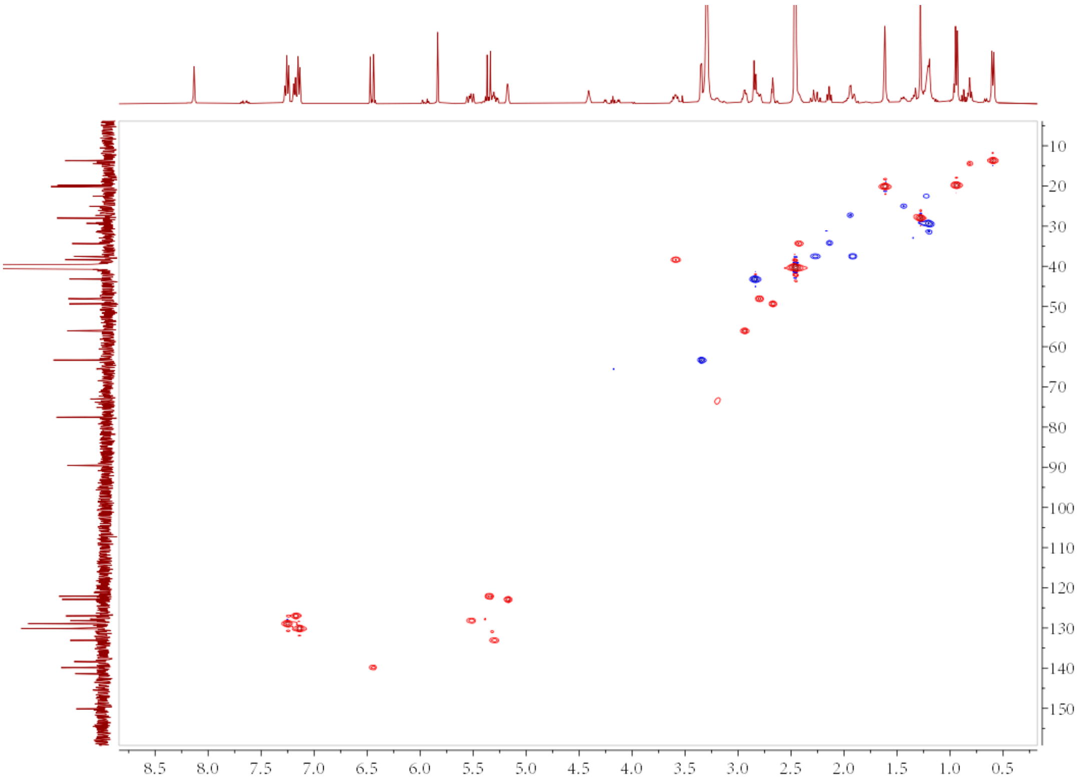

Figure S17. HSQC of compound **3** (in DMSO-d_6).

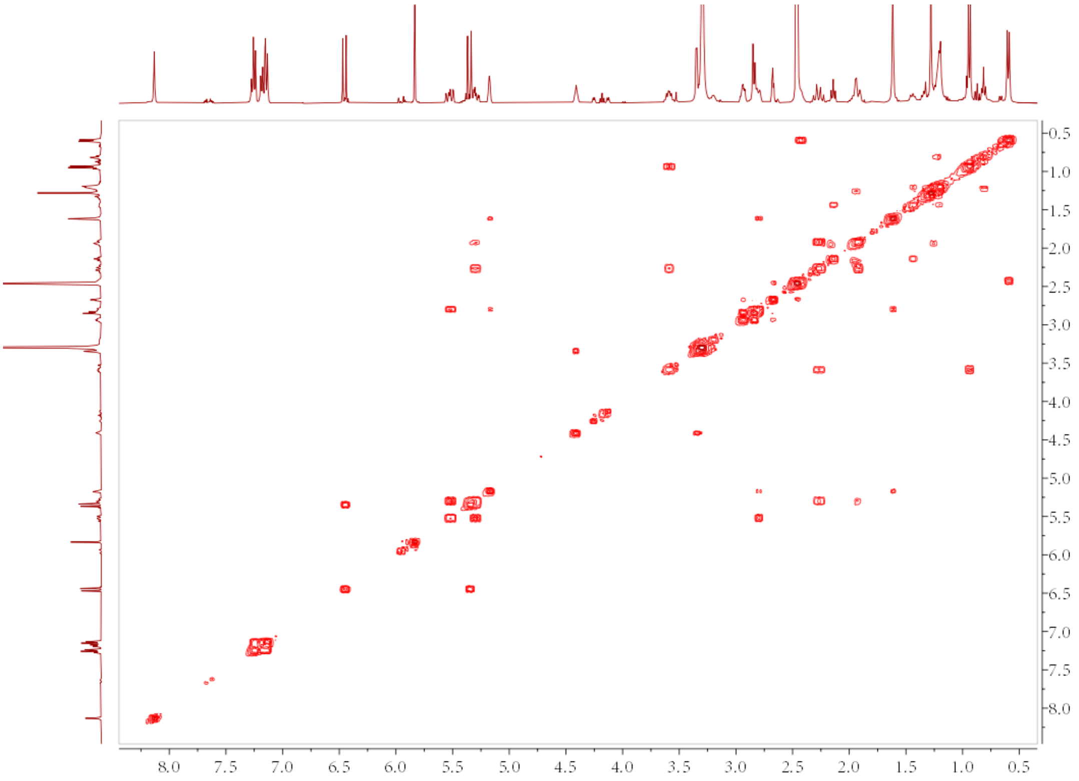

Figure S18. COSY of compound **3** (in DMSO-d_6).

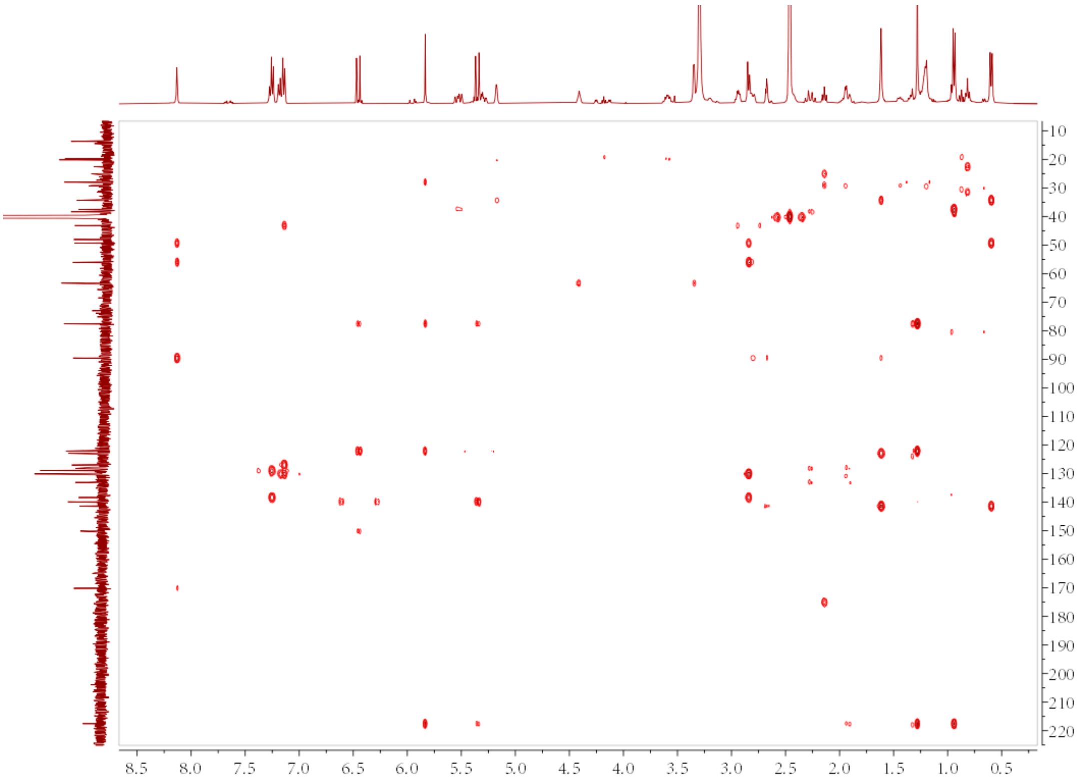

Figure S19. HMBC of compound **3** (in DMSO-d_6).

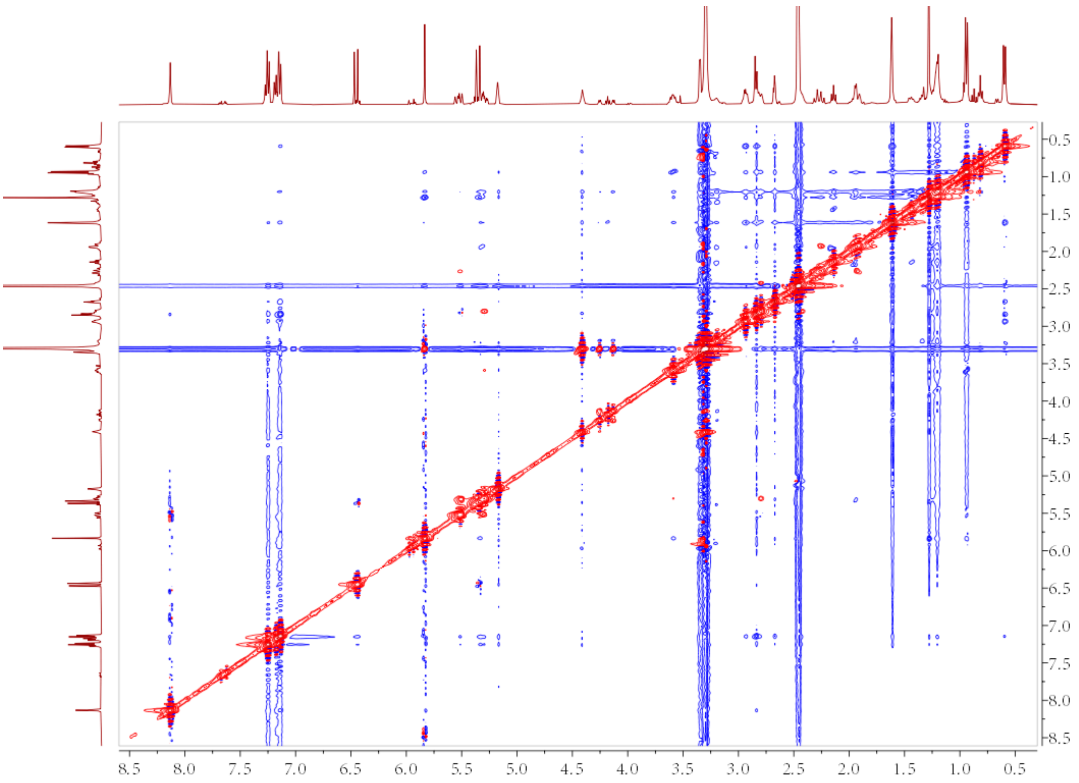

Figure S20. NOESY of compound **3** (in DMSO-d_6).

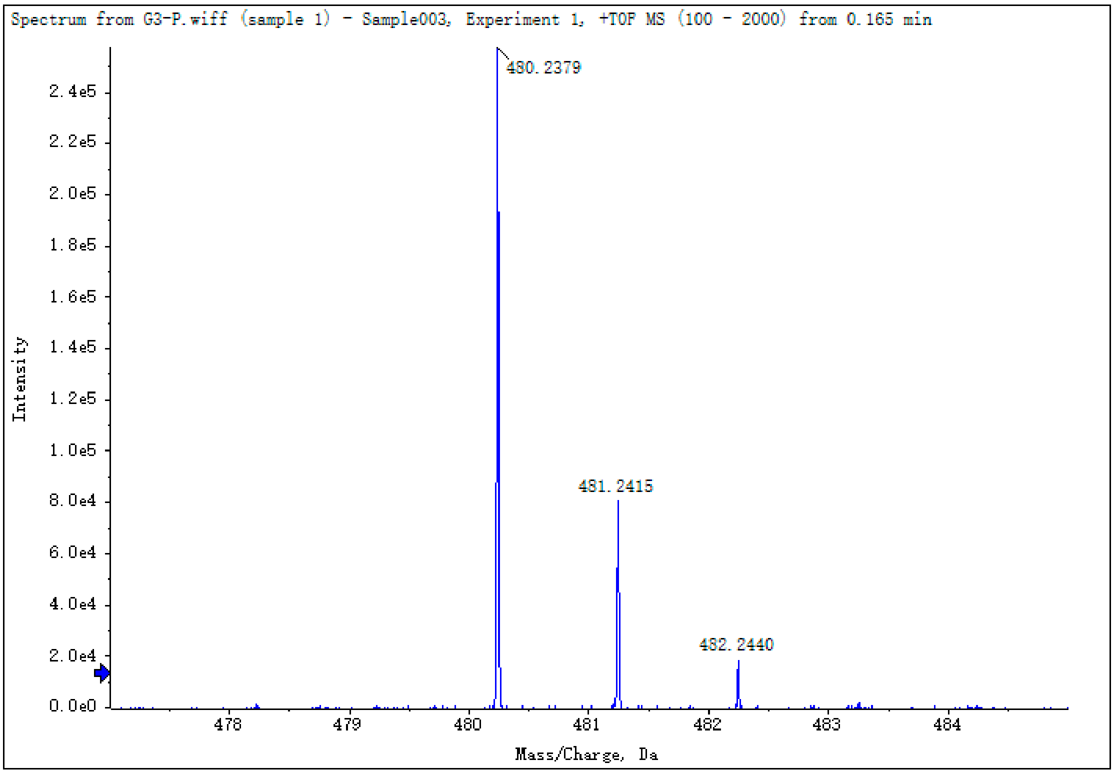

Figure S21. HRESIMS of compound **3**.

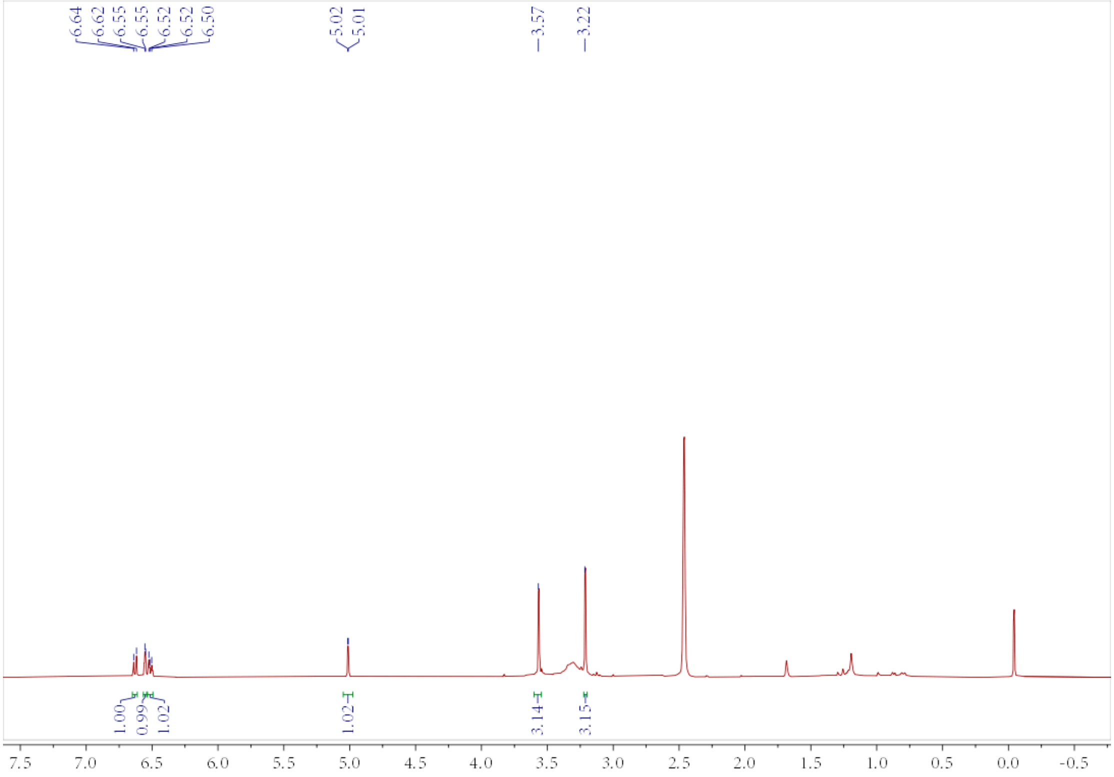

Figure S22. ^{1}H NMR of compound **4** (in DMSO-d_6, 600 MHz).

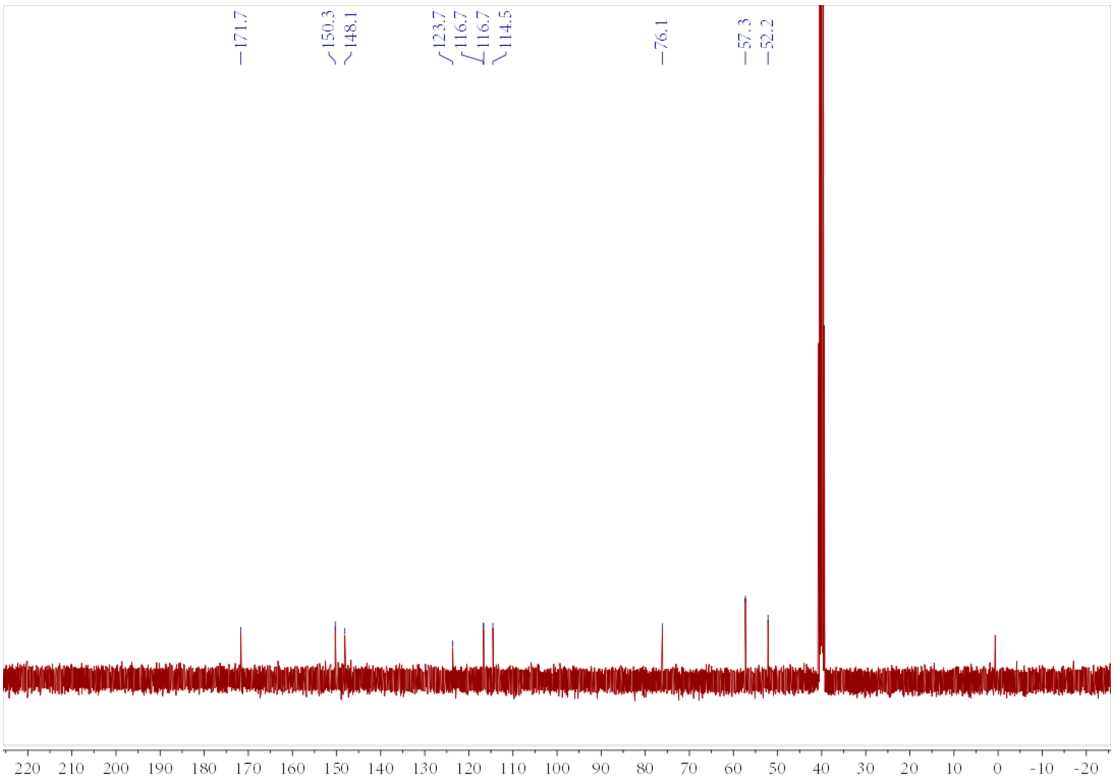

Figure S23. ^{13}C NMR of compound **4** (in DMSO-d_6, 150 MHz).

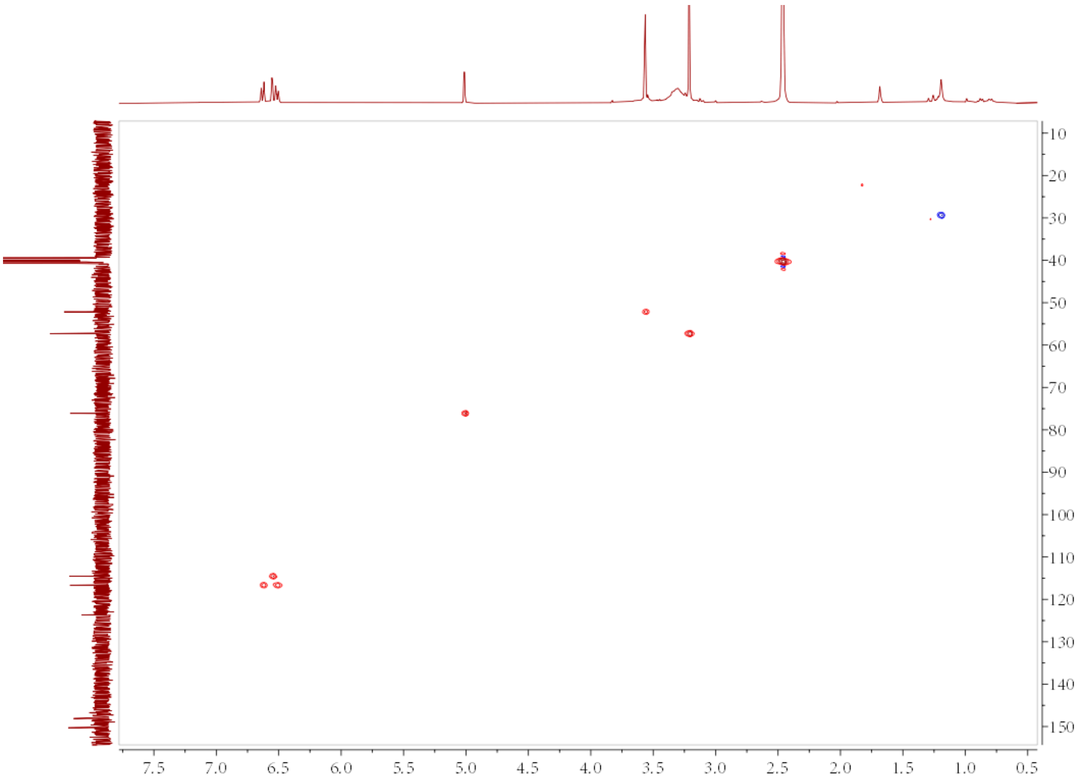

Figure S24. HSQC of compound **4** (in DMSO-d_6).

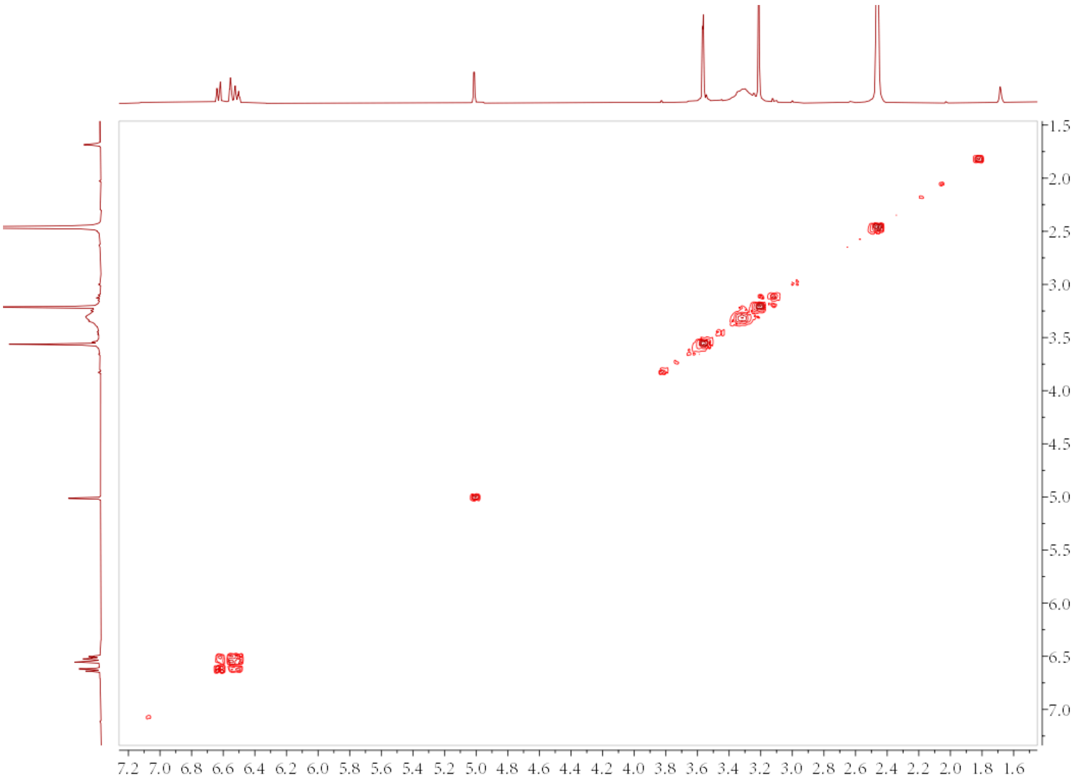

Figure S25. COSY of compound **4** (in DMSO-d_6).

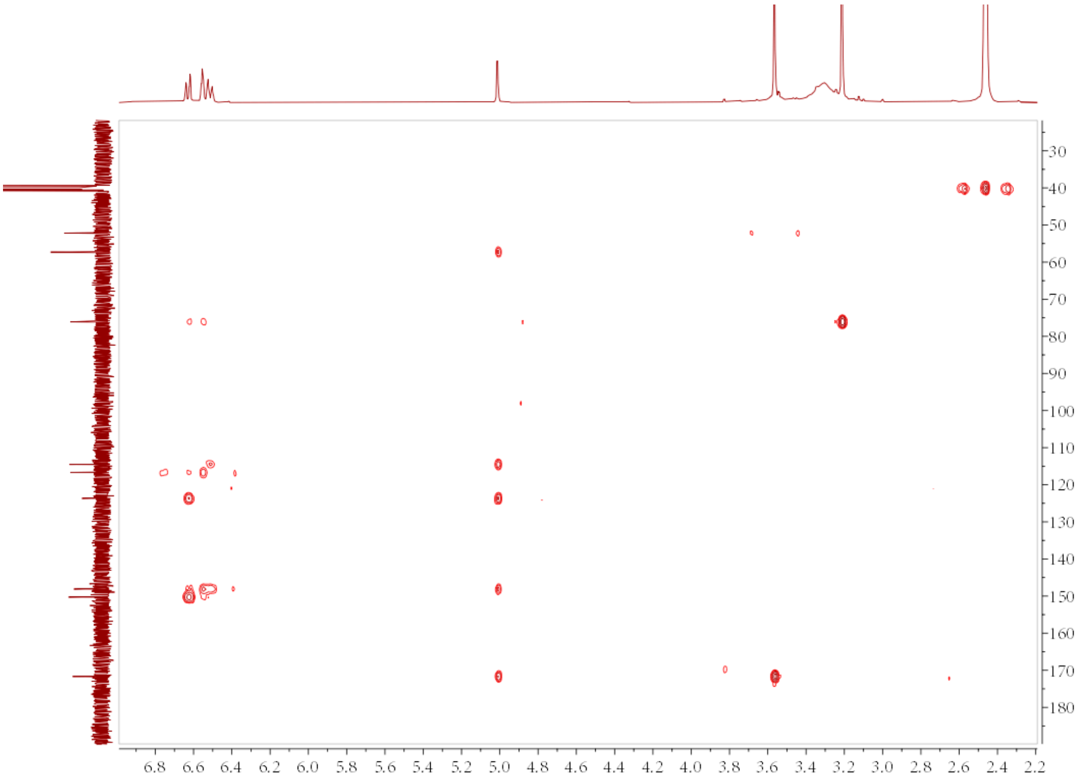

Figure S26. HMBC of compound **4** (in DMSO-d_6).

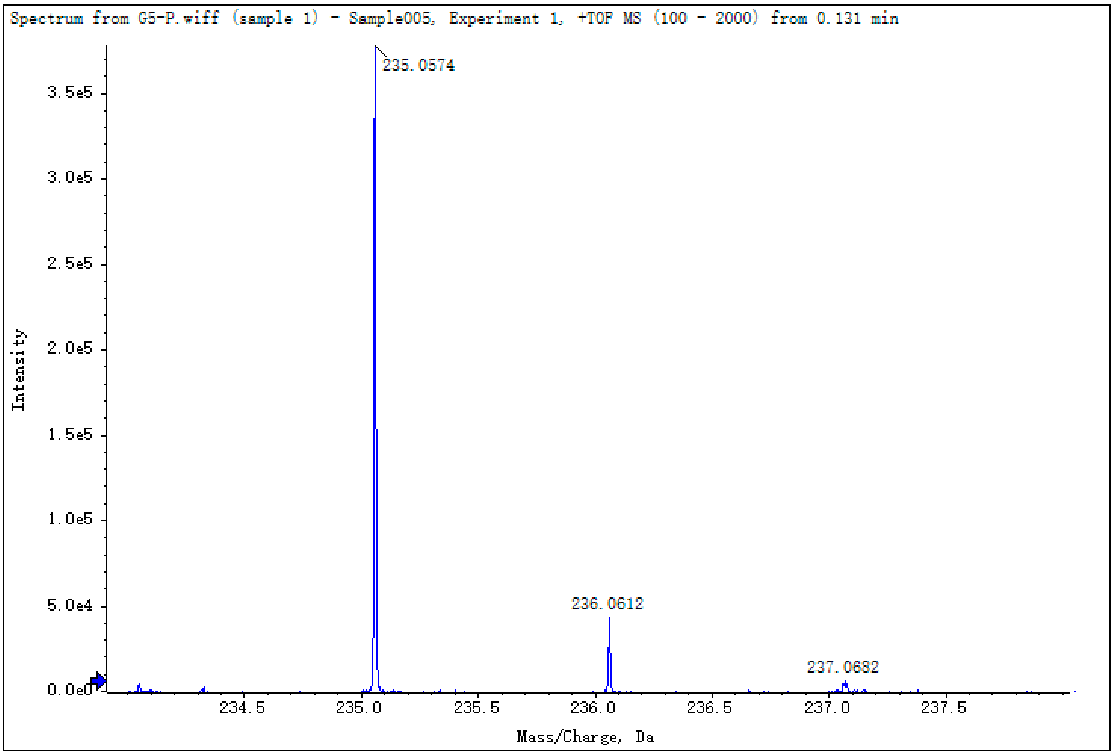

Figure S27. HRESIMS of compound **4**.

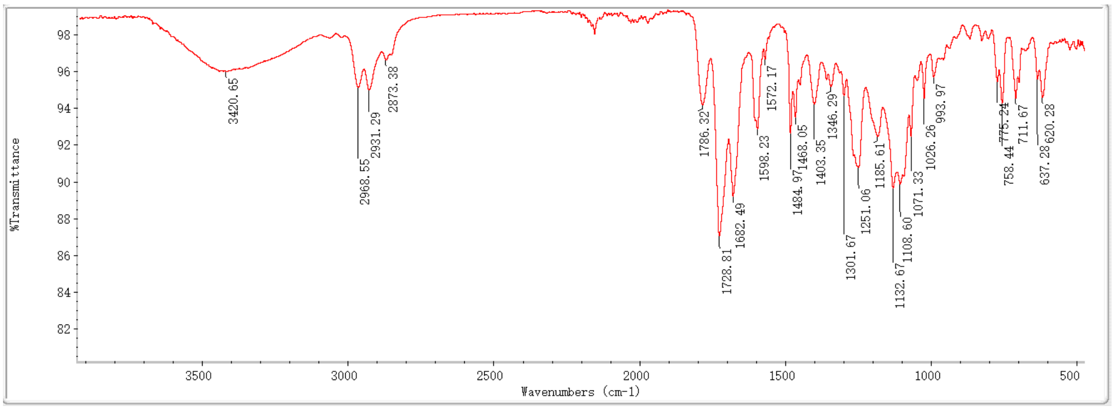

Figure S28. IR spectrum of compound **1**.

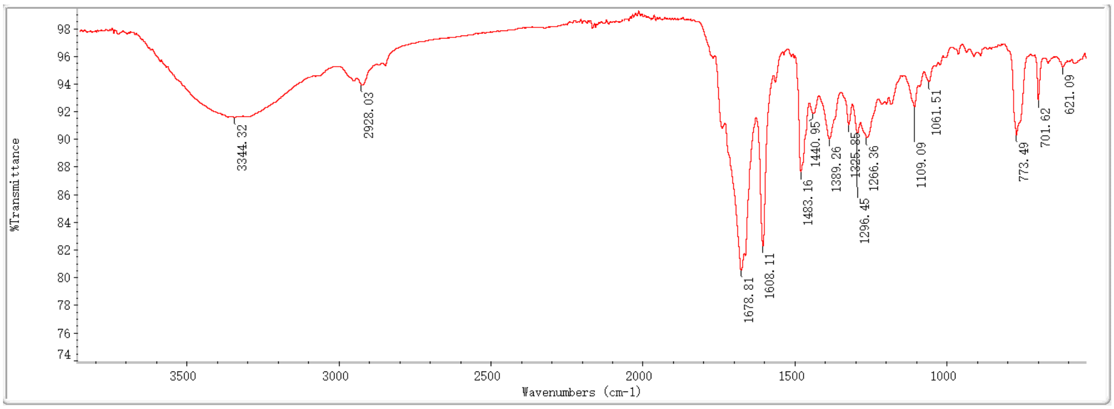

Figure S29. IR spectrum of compound **2**.

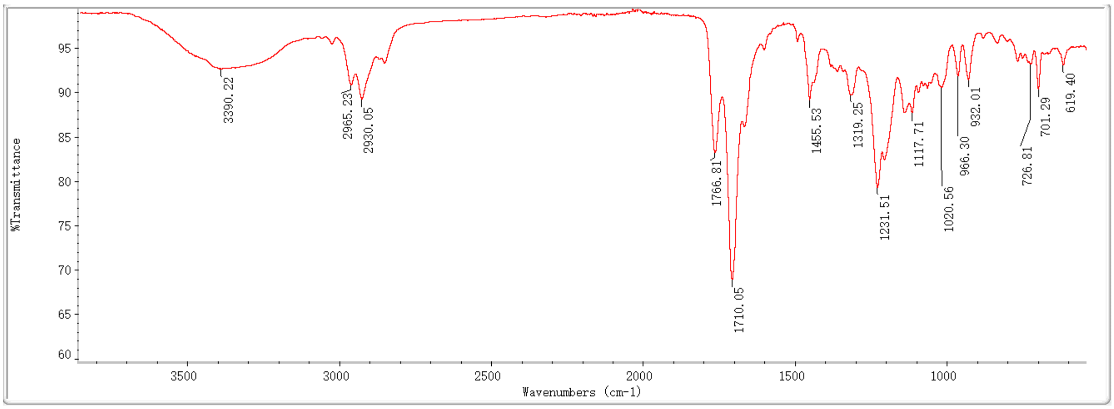

Figure S30. IR spectrum of compound **3**.

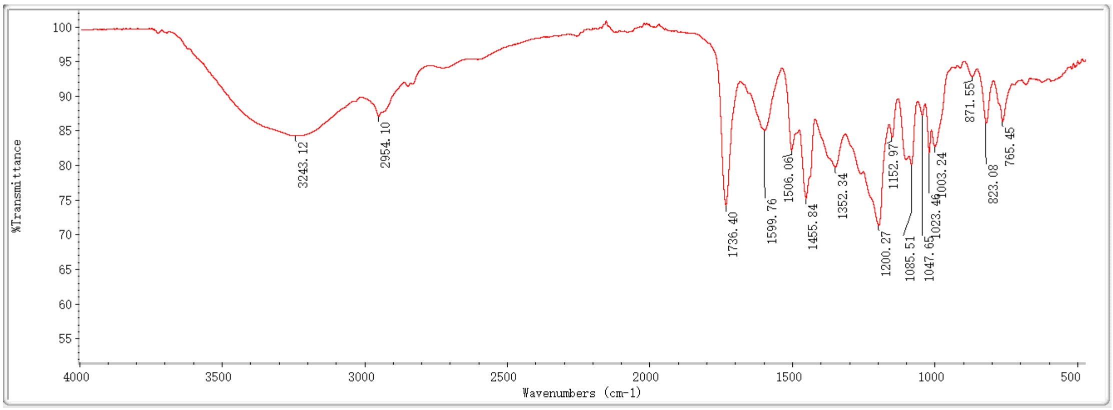

Figure S31. IR spectrum of compound **4**.

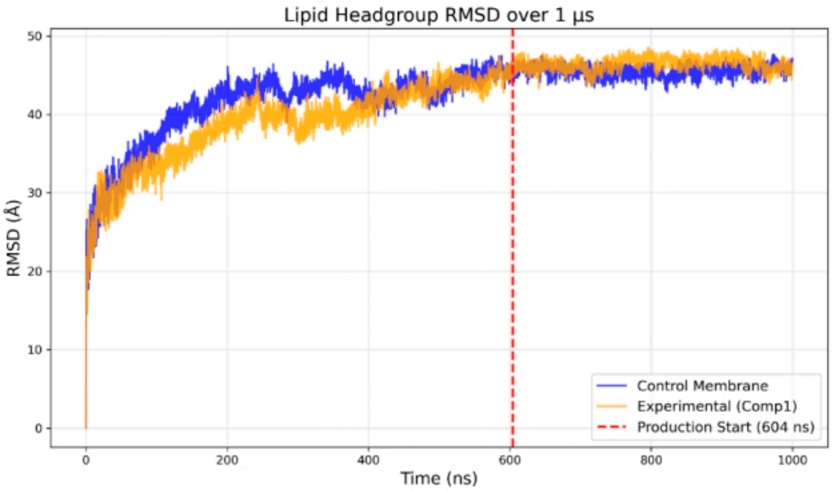

Figure S32. Thermodynamic equilibration of the simulated membrane systems.

Time evolution of the root-mean-square deviation (RMSD) for the lipid phosphorus

headgroups over the 1 µs trajectories. The control membrane (blue) and the experimental system containing the novel compound (orange) establish stable baselines at 558.5 ns and 604 ns, respectively. The final 394 ns sampling window denotes the fully equilibrated phase utilized for all subsequent structural extractions.

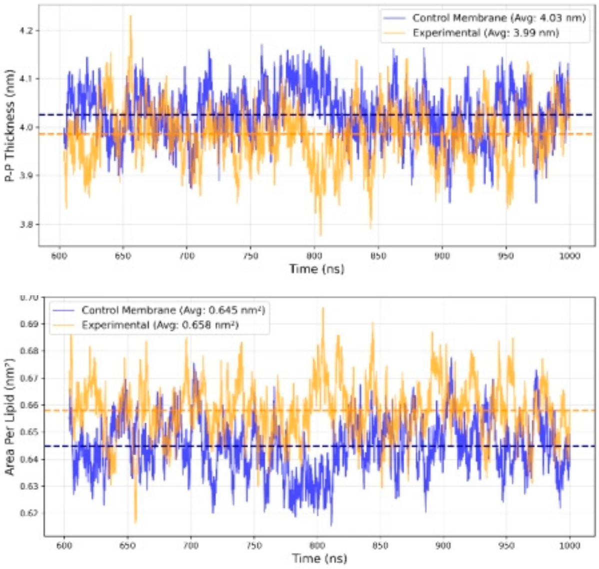

Figure S33. Macroscopic structural modulation of the membrane upon compound insertion. (A) Average Area Per Lipid (APL) and **(B)** overall membrane thickness for the control (blue) and experimental (orange) systems. Values and corresponding standard deviations were calculated exclusively from the fully equilibrated final 394 ns of the simulation trajectories. The lateral expansion and concurrent transverse thinning demonstrate the compound's capacity to physically disrupt the tightly packed palmitic acid (PAL) environment, inducing a more relaxed architectural state.

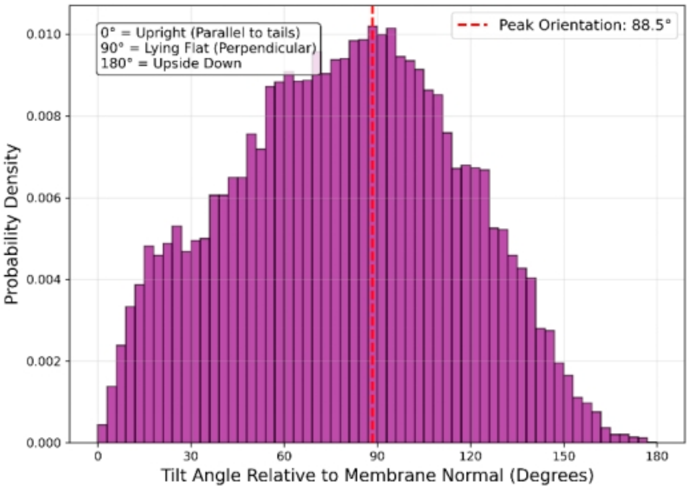

Figure S34. Orientational distribution of the compound within the lipid bilayer.

Probability distribution of the compound's tilt angle calculated relative to the membrane normal (Z-axis).

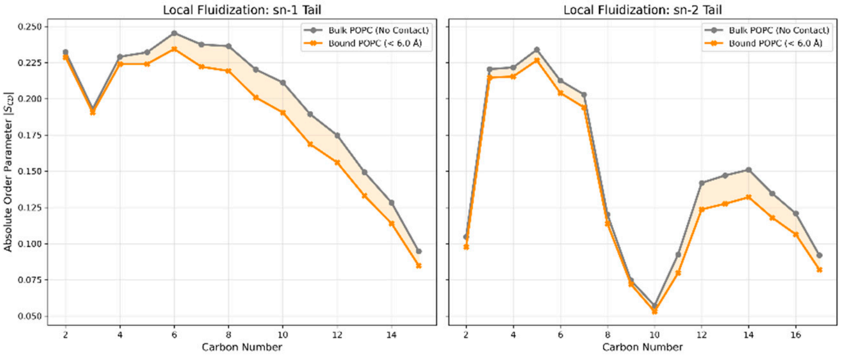

(A)

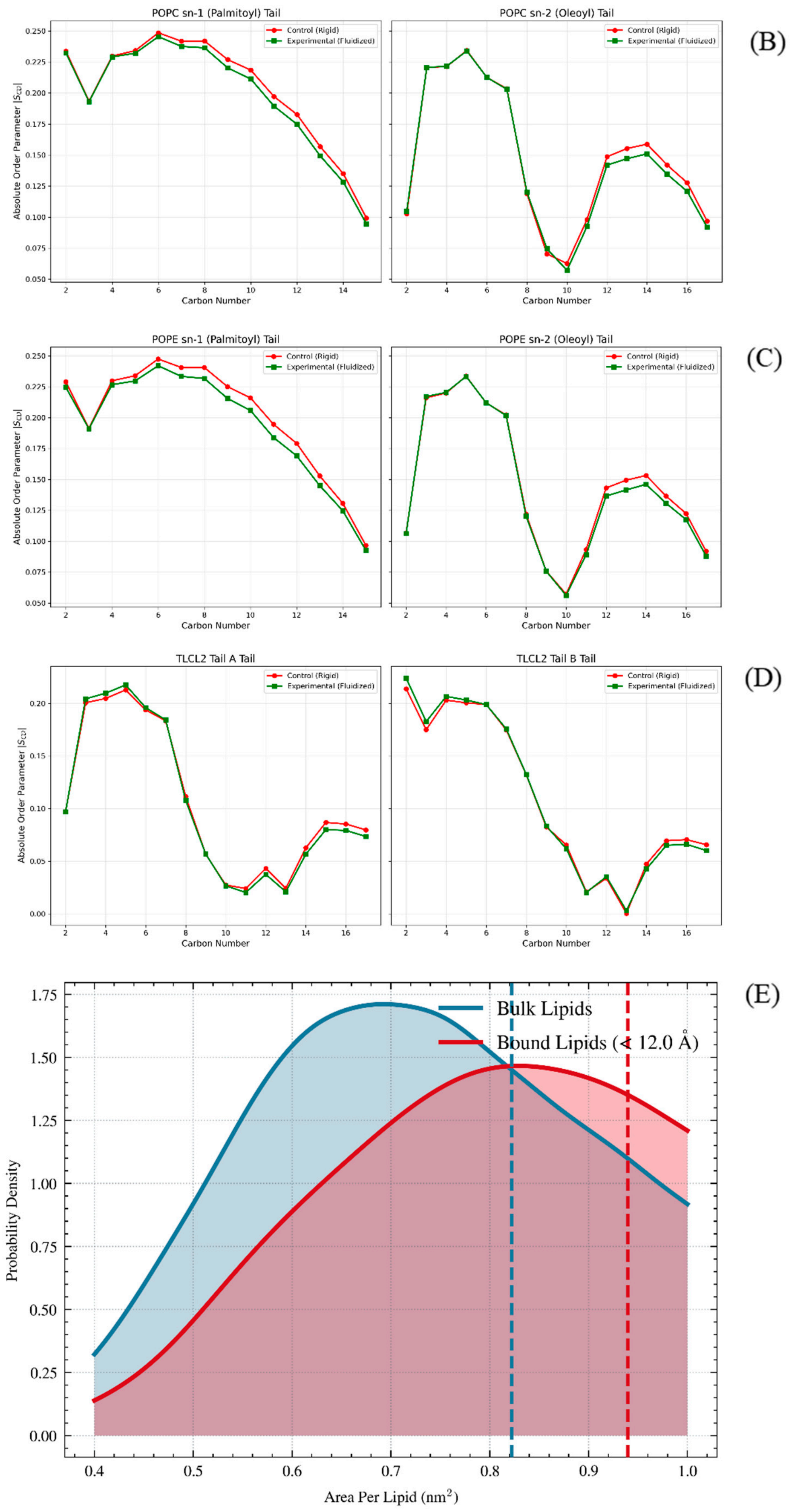
POPC sn-1 (Palmitoyl) Tail
POPC sn-2 (Oleoyl) Tail
(B)
POPE sn-1 (Palmitoyl) Tail
POPE sn-2 (Oleoyl) Tail
(C)
TLCL2 Tail A Tail
TLCL2 Tail B Tail
(D)
Control (Rigid)
Experimental (Fluidized)
Absolute Order Parameter |S_CD|
Carbon Number
(E)
Bulk Lipids
Bound Lipids (< 12.0 Å)
Probability Density
Area Per Lipid (nm²)

Figure S35. Compound-induced local and global membrane fluidization and structural disruption. (A) Comparison of deuterium order parameters (S_{CD}) for POPC acyl tails, contrasting the bulk lipid environment with lipids actively bound within the compound's local solvation shell (< 6.0 Å). **(B–D)** Global S_{CD} profiles comparing the control (black) and experimental (red) systems across the acyl tails of **(B)** POPC (sn-1, sn-2), **(C)** POPE (sn-1, sn-2), and **(D)** TLCL2 (tails A and B). The universal reduction in S_{CD} indicates a shift toward disordered, gauche tail conformations. **(E)** Local Area Per Lipid (APL) distributions calculated via 2D Voronoi tessellation. The analysis contrasts the tightly packed packing geometries of the unperturbed bulk lipids (> 12.0 Å) against the significant localized lateral expansion experienced by lipids confined to the compound's immediate solvation shell.

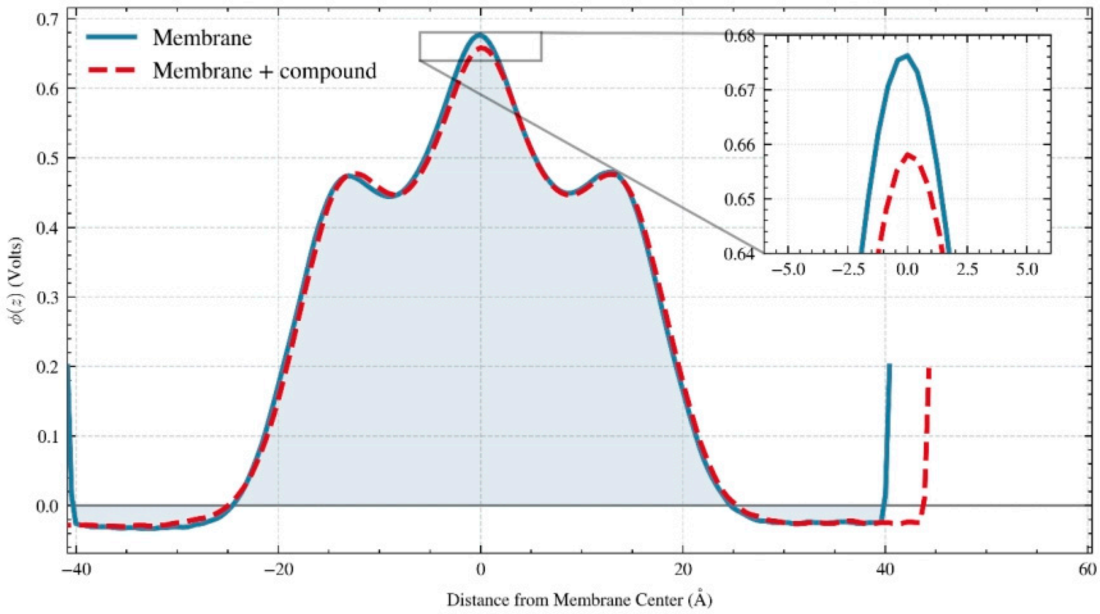

Figure S36. Spatial electrostatic potential profile of the simulated membrane systems. 1D electrostatic potential $\phi(z)$ mapped across the membrane normal (Z-axis) for the control (blue) and experimental (red) states. The maximum internal dipole potential decreases from 0.676 V in the highly ordered control to 0.658 V upon compound insertion.

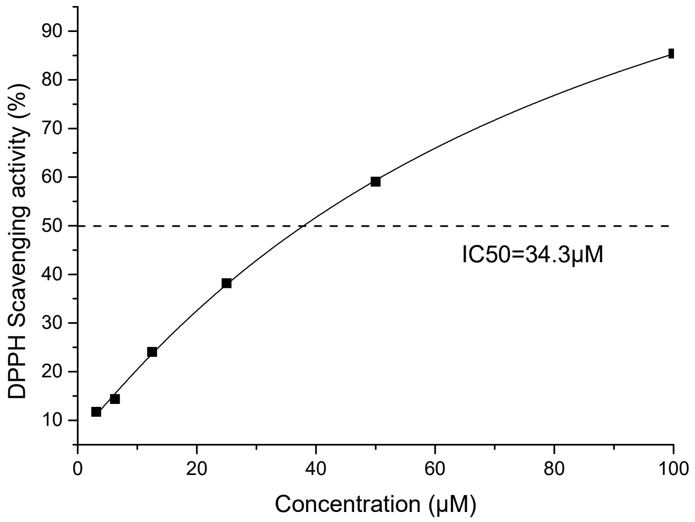

Figure S37. DPPH radical scavenging activity of compound 4.

Table S1. Key 2D NMR correlations for compound 1

Position	δH (ppm)	δC (ppm)	Key HMBC(H→C)	COSY	NOESY
2	5.24, s	86.7	C-3, C-10	-	H_b-13
5	8.04, d (7.7)	126.5	C-9, C-14	H-6	H-14
6	7.32, t (7.4)	125.8	C-4, C-8	H-5, H-7	-
7	7.50, m	132.0	C-5, C-9	H-6, H-8	-
8	7.47, m	115.3	C-4, C-6	H-7	-
13	a 3.32, m<br>b 2.99, dd (13.8, 9.6)	34.7	C-2	H-14	H-2
14	6.16, t (9.6)	55.3	C-3, C-15, C-17, C-25	H-13	H-5
19	8.16, d (7.9)	126.9	C-18, C-21	H-20	-
20	7.59, t (7.6)	128.7	-	H-19, H-21	-
21	7.86, t (7.6)	136.3	C-23	H-20, H-22	-
22	7.66, m	127.9	C-18	H-21	-
26	1.33, s	17.5	C-10, C-11, C-27	-	-
28	6.11, d (8.4)	76.8	C-25, C-32	H-29	-

29	2.62, m	32.9	-	H-28, H-30, H-31	-
30	1.24, d (6.6)	18.8	-	H-29	-
31	1.03, d (6.6)	18.3	C-28, C-30	H-29	-
34	7.98, d (7.1)	130.4	-	H-35	-
35	7.52, m	129.6	C-33	H-34	-
36	7.66, m	134.5	C-34	H-35, H-37	-
37	7.52, m	129.6	-	H-36, H-38	-
38	7.98, d (7.1)	129.9	C-32	H-37	-

Table S2. Key 2D NMR correlations for compound 2

Position	δH (ppm)	δC (ppm)	Key HMBC(H→C)	COSY	NOESY
2	5.32, s	83.0	C-10, C-11	-	H_b-13, H_3-26
5	7.32, m	125.3	-	H-6	-
6	7.09, t (7.3)	124.9	C-4, C-8	H-5, H-7	-
7	7.29, m	130.1	-	H-6, H-8	-
8	7.32, m	115.6	-	H-7	-
11	3.78, d (7.3)	61.0	-	H-26	-
13	a 2.97, dd (15.3, 9.0)<br>b 2.60, dd (15.3, 2.7)	36.1	C-2, C-3	H-14	H-2
14	5.19, d (8.0)	57.9	C-15, C-17, C-25	H-13	-
19	8.13, d (7.8)	126.7	C-17, C-21	H-20	-
20	7.54, t (7.8)	127.7	-	H-19, H-21	-
21	7.83, t (7.0)	135.2	C-23	H-20, H-22	-
22	7.66, d (8.1)	127.7	C-18	H-21	-
25	8.41, s	149.0	C-17, C-23		-
26	1.35, d (7.0)	18.3	C-10	H-11	H-2

Table S3. Key 2D NMR correlations for compound 3

Position	δH (ppm)	δC (ppm)	Key HMBC(H→C)	COSY	NOESY
2	8.13, s	-	C-1, C-9	-	-
3	2.94, m	56.0	C-1	H-4, H-10	H_3-11
4	2.67, t (3.7)	49.3	C-9	H-3, H-5	-
5	2.42, br	34.3	-	H-4, H-11	H2-10
7	5.17, s	122.9	-	H-8	-
8	2.80, m	48.1	C-9	H-7, H-13	H-14
10	2.84, d (6.8)	43.2	C-1’, C-6’	H-3	H-5
11	0.60, d (7.3)	13.7	C-4, C-6	H-5	H-3
12	1.62, s	20.2	C-5, C-6, C-7	-	-
13	5.53, dd (15.0, 11.0)	128.2	-	H-8, H-14	-
14	5.29, dd (15.0, 3.6)	133.1	-	H-13, H-15	H-8
15	2.27, q (11.1)<br>1.92, m	37.5	-	H-14, H-16	-
16	3.59, d (2.2)	38.4	-	H-15, H-23	-
19	5.35, d (12.4)	122.1	-	H-20	-
20	6.45, d (12.4)	139.9	C-22	H-19	-
23	0.94, d (6.9)	19.8	C-15, C-17	H-16	18-OH
24	1.28, s	28.0	C-17, C-18, C-19	-	-
2’	7.14, m	128.9	-	H-3’	-
3’	7.26, t (7.3)	130.1	C-1’, C-5’	H-2’, H-4’	-
4’	7.17, m	127.0	-	H-3’, H-5’	-
5’	7.26, t (7.3)	130.1	-	H-4’, H-6’	-
6’	7.14, m	128.9	-	H-5’	-
18-OH	5.83, s	-	-	-	H_3-23

Table S4. Key 2D NMR correlations for compound 4

Position	δH (ppm)	δC (ppm)	Key HMBC(H→C)	COSY
2	5.01, d (1.9)	76.1	C-3, C-4, C-8	-
5	6.64, d (8.6)	116.7	C-3, C-7	H-6
6	6.52, dd (8.6, 2.3)	116.7	-	H-5
1-OCH3	3.57, s	52.2	C-1	-
2-OCH3	3.22, s	57.3	C-2	-

Table S5. Top 15 highest-occupancy intermolecular hydrogen bond interactions.

Absolute hydrogen bonding events were calculated utilizing a 3.5 Å distance and >150° angle geometric cutoff. Occupancy represents the percentage of the simulated timeframe that the specific donor-acceptor pair remained active per compound.

Rank	Donor Atom	Acceptor Lipid (Atom)	Active Frames	Average Occupancy
1	Compound (C3-OH)	POPC (O22)	1774	9.0%
2	Compound (C2-NH)	POPC (O22)	1616	8.2%
3	Compound (C3-OH)	POPE (O32)	1394	7.0%
4	Compound (C3-OH)	POPC (O32)	1323	6.7%
5	Compound (C3-OH)	POPC (O13)	1216	6.1%
6	Compound (C2-NH)	POPC (O13)	1214	6.1%
7	Compound (C3-OH)	POPE (O22)	1191	6.0%
8	Compound (C2-NH)	POPE (O32)	1114	5.6%
9	Compound (C2-NH)	POPE (O22)	1110	5.6%
10	Compound (C2-NH)	POPC (O32)	996	5.0%
11	Compound (C3-OH)	POPC (O11)	826	4.2%
12	Compound (C3-OH)	TLCL2 (OB1)	640	3.2%
13	Compound (C3-OH)	TLCL2 (OP14)	592	3.0%

14	Compound (C3-OH)	TLCL2 (OP12)	559	2.8%
15	Compound (C3-OH)	TLCL2 (OC1)	531	2.7%

Table S6. Highest-occupancy intermolecular hydrogen bonds formed specifically between the compound and TLCL2.

Rank	Compound Atom (Donor)	TLCL2 Atom (Acceptor)	Total H-Bond Count	Occupancy (%)*
1	com (C3-OH)	TLCL2 (OB1)	640	3.23%
2	com (C3-OH)	TLCL2 (OP14)	592	2.99%
3	com (C3-OH)	TLCL2 (OP12)	559	2.82%
4	com (C3-OH)	TLCL2 (OC1)	531	2.68%
5	com (C2-NH)	TLCL2 (OP14)	384	1.94%
6	com (C2-NH)	TLCL2 (OB1)	267	1.35%
7	com (C3-OH)	TLCL2 (OA1)	257	1.30%
8	com (C3-OH)	TLCL2 (OP13)	229	1.16%
9	com (C5-H)	TLCL2 (O12)	217	1.10%
10	com (C2-NH)	TLCL2 (OP12)	208	1.05%

Table S7. MD simulation parameters at-a-glance

Parameter	Detail
Force field	CHARMM36 (lipids) + CGenFF
Membrane composition	POPC/POPE/TLCL2/PAL = 45:34:11:10 (200 lipids total)
Water model	TIP3P
Ions	Neutralizing Na^+/Cl^- (0.15 M)
Temperature	303.15 K (v-rescale, τ = 1.0 ps)
Pressure	1.0 bar (C-rescale, semi-isotropic, τ = 5.0 ps)
Simulation time	1 μs per system (control + experimental)
Replicates	Single trajectory per system (*limitation acknowledged*)
Electrostatics	PME, cut-off 1.2 nm

Van der Waals	Force-switch, 1.0–1.2 nm
Constraints	LINCS (all H-bonds), 2 fs time step
Equilibration	Standard CHARMM-GUI 6-step protocol
Convergence criteria	RMSD of phosphorus headgroups; sliding window 50 ns; slope < 0.001
Analysis metrics	APL, membrane thickness, deuterium order parameters (Scd), hydrogen bonds, Voronoi APL, electrostatic potential

Table S8. Optimized conformers and their boltzmann populations.

Conformers	Boltzmann populations	Conformers	Boltzmann populations
1-1	0.3176	2-7	0.0889
1-2	0.3176	2-8	0.0831
1-3	0.2345	2-9	0.0643
1-4	0.0534	2-10	0.0223
1-5	0.0383	2-11	0.0204
1-6	0.0212	2-12	0.0179
1-7	0.0174	3-1	0.9916
2-1	0.1382	3-2	0.0084
2-2	0.1225	4-1	0.4510
2-3	0.1194	4-2	0.2880
2-4	0.1177	4-3	0.2050
2-5	0.1071	4-4	0.0560
2-6	0.0982		

Table S9. Optimized Z-matrixes of conformers.

1-1							
C	4.0211	0.5534	-2.4528	O	0.6416	1.6092	3.5614
C	5.0900	1.1867	-1.8183	C	1.2286	-4.2193	-0.8184
C	4.9901	1.6524	-0.5076	C	2.5391	-4.6204	-1.0354
C	3.7761	1.4658	0.1383	C	3.2275	-4.1762	-2.1624

C	2.6862	0.8547	-0.4930	C	2.6014	-3.3328	-3.0764
C	2.8030	0.3917	-1.7913	C	1.2919	-2.9223	-2.8622
N	3.4175	1.8029	1.4471	H	4.1366	0.1913	-3.4697
C	2.1180	1.2322	1.7755	H	6.0270	1.3148	-2.3525
C	1.4642	0.9425	0.3965	H	5.8173	2.1425	-0.0071
C	0.4828	-0.2428	0.3853	H	1.9648	-0.0888	-2.2905
C	-0.6932	0.2672	-0.4454	H	2.2329	0.2668	2.3071
C	-0.5342	1.7898	-0.4627	H	0.9482	-1.1336	-0.0448
O	0.7041	2.1117	-0.0407	H	0.1647	-0.4733	1.4048
C	3.7236	2.8883	2.2372	H	-0.5901	-0.0459	-1.4846
C	2.6214	2.9491	3.3115	H	2.3652	2.2313	5.3417
N	1.5058	2.2462	2.6163	H	4.0281	2.7228	4.9306
C	3.1309	2.2166	4.5626	H	3.3851	1.1732	4.3480
C	2.2295	4.3829	3.6253	H	1.8873	4.8924	2.7209
O	4.6845	3.6183	2.1206	H	1.4198	4.3868	4.3617
O	-1.3279	2.5836	-0.8866	H	3.0873	4.9240	4.0346
N	-2.0202	-0.1272	0.0277	H	-3.5499	1.1943	3.5557
C	-2.3929	0.4310	1.2659	H	-5.7982	0.4955	4.3757
C	-3.6959	0.0362	1.7542	H	-7.1827	-1.0470	3.0136
C	-4.4771	-0.8382	0.9732	H	-6.3369	-1.8989	0.8335
N	-4.0407	-1.3221	-0.2382	H	-3.2524	-2.2703	-2.2425
C	-2.8738	-0.9676	-0.6684	H	-1.9500	0.3780	-3.0536
C	-4.1741	0.5178	2.9808	H	-2.7643	-0.2236	-5.3124
C	-5.4205	0.1275	3.4270	H	-1.7389	-1.5302	-4.6959
C	-6.2029	-0.7458	2.6539	H	-3.5045	-1.7045	-4.6850
C	-5.7441	-1.2245	1.4431	H	-4.2463	1.0639	-3.7855
O	-1.6194	1.1966	1.8401	H	-4.1056	0.9482	-2.0287
C	-2.5524	-1.4439	-2.0751	H	-4.9255	-0.3380	-2.9335
O	-1.2017	-1.9458	-2.2619	H	-0.2388	1.7396	3.1718
C	-2.7583	-0.3614	-3.1508	H	0.6731	-4.5527	0.0526
C	-2.6801	-0.9957	-4.5416	H	3.0275	-5.2801	-0.3248
C	-4.0885	0.3685	-2.9559	H	4.2541	-4.4889	-2.3294
C	-0.7865	-2.9337	-1.4258	H	3.1369	-2.9928	-3.9577
O	-1.4679	-3.3708	-0.5259	H	0.7999	-2.2668	-3.5728
C	0.6016	-3.3623	-1.7279				
1-2							
C	4.0211	0.5534	-2.4528	O	0.6416	1.6092	3.5614
C	5.0900	1.1867	-1.8183	C	1.2286	-4.2193	-0.8184
C	4.9901	1.6524	-0.5076	C	2.5391	-4.6204	-1.0354
C	3.7761	1.4658	0.1383	C	3.2275	-4.1762	-2.1624
C	2.6862	0.8547	-0.4930	C	2.6014	-3.3328	-3.0764
C	2.8030	0.3917	-1.7913	C	1.2919	-2.9223	-2.8622

N	3.4175	1.8029	1.4471	H	4.1366	0.1913	-3.4697
C	2.1180	1.2322	1.7755	H	6.0270	1.3148	-2.3525
C	1.4642	0.9425	0.3965	H	5.8173	2.1425	-0.0071
C	0.4828	-0.2428	0.3853	H	1.9648	-0.0888	-2.2905
C	-0.6932	0.2672	-0.4454	H	2.2329	0.2668	2.3071
C	-0.5342	1.7898	-0.4627	H	0.9482	-1.1336	-0.0448
O	0.7041	2.1117	-0.0407	H	0.1647	-0.4733	1.4048
C	3.7236	2.8883	2.2372	H	-0.5901	-0.0459	-1.4846
C	2.6214	2.9491	3.3115	H	2.3652	2.2313	5.3417
N	1.5058	2.2462	2.6163	H	4.0281	2.7228	4.9306
C	3.1309	2.2166	4.5626	H	3.3851	1.1732	4.3480
C	2.2295	4.3829	3.6253	H	1.8873	4.8924	2.7209
O	4.6845	3.6183	2.1206	H	1.4198	4.3868	4.3617
O	-1.3279	2.5836	-0.8866	H	3.0873	4.9240	4.0346
N	-2.0202	-0.1272	0.0277	H	-3.5499	1.1943	3.5557
C	-2.3929	0.4310	1.2659	H	-5.7982	0.4955	4.3757
C	-3.6959	0.0362	1.7542	H	-7.1827	-1.0470	3.0136
C	-4.4771	-0.8382	0.9732	H	-6.3369	-1.8989	0.8335
N	-4.0407	-1.3221	-0.2382	H	-3.2524	-2.2703	-2.2425
C	-2.8738	-0.9676	-0.6684	H	-1.9500	0.3780	-3.0536
C	-4.1741	0.5178	2.9808	H	-2.7643	-0.2236	-5.3124
C	-5.4205	0.1275	3.4270	H	-1.7389	-1.5302	-4.6959
C	-6.2029	-0.7458	2.6539	H	-3.5045	-1.7045	-4.6850
C	-5.7441	-1.2245	1.4431	H	-4.2463	1.0639	-3.7855
O	-1.6194	1.1966	1.8401	H	-4.1056	0.9482	-2.0287
C	-2.5524	-1.4439	-2.0751	H	-4.9255	-0.3380	-2.9335
O	-1.2017	-1.9458	-2.2619	H	-0.2388	1.7396	3.1718
C	-2.7583	-0.3614	-3.1508	H	0.6731	-4.5527	0.0526
C	-2.6801	-0.9957	-4.5416	H	3.0275	-5.2801	-0.3248
C	-4.0885	0.3685	-2.9559	H	4.2541	-4.4889	-2.3294
C	-0.7865	-2.9337	-1.4258	H	3.1369	-2.9928	-3.9577
O	-1.4679	-3.3708	-0.5259	H	0.7999	-2.2668	-3.5728
C	0.6016	-3.3623	-1.7279				
1-3							
C	1.5995	-0.9624	-4.3702	O	0.4595	3.8733	0.5272
C	2.1317	0.0926	-5.1121	C	1.9985	-3.6450	-0.8439
C	2.1780	1.3915	-4.6069	C	3.1201	-3.7863	-1.6510
C	1.6645	1.5929	-3.3334	C	4.3724	-3.4000	-1.1808
C	1.1063	0.5495	-2.5852	C	4.5070	-2.8804	0.1050
C	1.0725	-0.7348	-3.0982	C	3.3918	-2.7418	0.9188
N	1.6111	2.7729	-2.5853	H	1.5886	-1.9637	-4.7891
C	1.1819	2.4861	-1.2232	H	2.5266	-0.1002	-6.1054

C	0.4856	1.1012	-1.3194	H	2.5913	2.2156	-5.1770
C	0.5793	0.2477	-0.0420	H	0.6409	-1.5526	-2.5260
C	-0.8232	-0.3380	0.1082	H	2.0540	2.3792	-0.5480
C	-1.7142	0.5380	-0.7776	H	1.3487	-0.5228	-0.1394
O	-0.9386	1.2825	-1.5900	H	0.8273	0.8815	0.8129
C	1.3766	4.0829	-2.9387	H	-0.8680	-1.3349	-0.3307
C	0.9117	4.7871	-1.6494	H	2.9174	4.7082	-0.7651
N	0.3626	3.6347	-0.8800	H	1.8442	5.9335	-0.0620
C	2.1370	5.4420	-0.9928	H	2.5527	6.1869	-1.6776
C	-0.1802	5.8042	-1.9325	H	-1.0344	5.3245	-2.4173
O	1.5382	4.5756	-4.0346	H	-0.5135	6.2532	-0.9916
O	-2.9120	0.5038	-0.8377	H	0.2039	6.5914	-2.5875
N	-1.3351	-0.4106	1.4772	H	-2.2207	2.9149	3.6427
C	-1.5891	0.8385	2.0765	H	-3.0903	2.7905	5.9761
C	-2.0927	0.7825	3.4312	H	-3.4448	0.5802	7.0434
C	-2.2932	-0.4770	4.0297	H	-2.9347	-1.5123	5.7958
N	-2.0218	-1.6514	3.3669	H	-1.3982	-3.6612	2.3050
C	-1.5712	-1.5951	2.1560	H	-2.5232	-2.6429	-0.3547
C	-2.3812	1.9607	4.1340	H	-1.5292	-4.9621	-0.3955
C	-2.8640	1.8832	5.4247	H	-2.5892	-5.4188	0.9519
C	-3.0647	0.6305	6.0271	H	-3.2868	-4.9883	-0.6174
C	-2.7860	-0.5366	5.3446	H	-4.7487	-3.3496	0.5442
O	-1.3808	1.8689	1.4368	H	-4.0919	-1.9561	1.4096
C	-1.4252	-2.9457	1.4752	H	-4.0242	-3.5650	2.1509
O	-0.2109	-3.1075	0.6947	H	-0.3575	3.4751	0.8706
C	-2.5935	-3.2889	0.5326	H	1.0215	-3.9465	-1.2058
C	-2.4860	-4.7498	0.0892	H	3.0180	-4.1961	-2.6514
C	-3.9413	-3.0195	1.2045	H	5.2458	-3.5048	-1.8178
C	0.9680	-2.9075	1.3417	H	5.4844	-2.5815	0.4713
O	1.0379	-2.5640	2.5002	H	3.4721	-2.3380	1.9232
C	2.1312	-3.1175	0.4448				
1-4							
C	3.8599	-4.0313	-0.9971	O	3.5026	2.6144	1.1021
C	5.2410	-3.8410	-0.9450	C	-6.2911	-0.9029	-2.6968
C	5.7967	-2.6221	-0.5585	C	-7.6704	-1.0091	-2.8059
C	4.9155	-1.6006	-0.2326	C	-8.4311	-1.3780	-1.6985
C	3.5271	-1.7681	-0.2989	C	-7.8099	-1.6394	-0.4798
C	2.9915	-2.9857	-0.6808	C	-6.4292	-1.5378	-0.3648
N	5.2021	-0.3023	0.2005	H	3.4598	-4.9958	-1.2931
C	3.9805	0.3681	0.6227	H	5.9022	-4.6622	-1.2055
C	2.8412	-0.4394	-0.0590	H	6.8685	-2.4663	-0.5160
C	1.5106	-0.4412	0.7160	H	1.9150	-3.1284	-0.7483

C	0.4509	-0.2322	-0.3656	H	3.8447	0.2889	1.7198
C	1.2314	0.3230	-1.5705	H	1.3821	-1.3776	1.2650
O	2.5512	0.1293	-1.3688	H	1.4960	0.3863	1.4295
C	6.1885	0.5908	-0.1558	H	0.0434	-1.1871	-0.7045
C	5.6525	1.9834	0.2276	H	5.8207	3.3181	1.9286
N	4.1822	1.7409	0.1964	H	7.2851	2.3633	1.5842
C	6.1922	2.3376	1.6216	H	5.8888	1.6023	2.3743
C	6.0388	3.0295	-0.8042	H	5.6625	2.7502	-1.7916
O	7.2658	0.3203	-0.6407	H	5.6099	3.9965	-0.5230
O	0.7651	0.7677	-2.5786	H	7.1274	3.1225	-0.8523
N	-0.6643	0.6468	-0.0064	H	-0.1876	4.6472	0.5489
C	-0.3165	2.0025	0.1519	H	-2.1001	6.1509	1.0947
C	-1.4152	2.8879	0.4733	H	-4.3850	5.2115	1.3092
C	-2.7107	2.3476	0.5905	H	-4.7760	2.7743	0.9807
N	-2.9607	1.0054	0.4154	H	-1.7861	-1.6371	-0.9498
C	-1.9804	0.2211	0.1215	H	-0.7976	-1.9114	1.3346
C	-1.1960	4.2605	0.6548	H	-2.2508	-2.2677	3.2992
C	-2.2604	5.0866	0.9548	H	-2.4681	-0.6220	2.6745
C	-3.5535	4.5526	1.0754	H	-3.6857	-1.8595	2.3422
C	-3.7822	3.2027	0.8969	H	-1.6833	-4.1930	1.7204
O	0.8572	2.3449	0.0154	H	-1.5053	-3.8942	-0.0181
C	-2.2842	-1.2642	-0.0455	H	-3.1087	-3.8284	0.7334
O	-3.6899	-1.4502	-0.2163	H	2.6971	2.8493	0.6126
C	-1.8683	-2.1014	1.1780	H	-5.6783	-0.6134	-3.5445
C	-2.6114	-1.6826	2.4473	H	-8.1554	-0.8029	-3.7553
C	-2.0508	-3.5915	0.8831	H	-9.5109	-1.4598	-1.7849
C	-4.1907	-1.0388	-1.4148	H	-8.4046	-1.9219	0.3838
O	-3.4918	-0.6338	-2.3157	H	-5.9408	-1.7320	0.5834
C	-5.6648	-1.1709	-1.4763				
1-5							
C	3.9310	-0.5725	-2.3977	O	1.0160	1.0278	3.7424
C	5.1387	-0.3264	-1.7442	C	0.4483	-3.9644	-1.4508
C	5.1787	0.0697	-0.4075	C	1.6264	-4.5272	-1.9210
C	3.9624	0.2171	0.2440	C	2.1347	-4.1468	-3.1610
C	2.7416	0.0018	-0.4061	C	1.4620	-3.2032	-3.9329
C	2.7181	-0.3985	-1.7295	C	0.2905	-2.6242	-3.4619
N	3.7163	0.5601	1.5771	H	3.9334	-0.8977	-3.4334
C	2.3059	0.3730	1.8924	H	6.0730	-0.4547	-2.2832
C	1.5989	0.3919	0.5074	H	6.1130	0.2574	0.1090
C	0.3067	-0.4383	0.4228	H	1.7774	-0.5787	-2.2442
C	-0.6693	0.4704	-0.3257	H	2.1320	-0.6213	2.3499
C	-0.0559	1.8726	-0.2149	H	0.4864	-1.3830	-0.0940

O	1.2306	1.7633	0.1692	H	-0.0671	-0.6589	1.4255
C	4.3278	1.4522	2.4280	H	-0.6834	0.2468	-1.3926
C	3.2938	1.7603	3.5268	H	2.8547	1.0207	5.5186
N	2.0193	1.4555	2.8171	H	4.5853	1.0326	5.0922
C	3.5762	0.8339	4.7197	H	3.5128	-0.2225	4.4376
C	3.3357	3.2233	3.9341	H	3.1529	3.8660	3.0691
O	5.4610	1.8746	2.3391	H	2.5647	3.4154	4.6869
O	-0.5733	2.9098	-0.5252	H	4.3152	3.4660	4.3561
N	-2.0501	0.4477	0.1650	H	-3.0419	2.0215	3.7872
C	-2.2151	1.0203	1.4447	H	-5.3870	2.0423	4.6330
C	-3.5677	1.0366	1.9531	H	-7.2037	1.0685	3.2540
C	-4.5938	0.4845	1.1633	H	-6.6956	0.0701	1.0319
N	-4.3400	-0.0775	-0.0646	H	-3.8350	-1.4396	-1.9265
C	-3.1292	-0.0835	-0.5233	H	-4.3630	0.8131	-2.6682
C	-3.8543	1.5984	3.2054	H	-2.7117	2.2145	-3.8995
C	-5.1553	1.6080	3.6656	H	-2.2260	2.0435	-2.2135
C	-6.1829	1.0567	2.8825	H	-1.4149	1.0959	-3.4810
C	-5.9131	0.5010	1.6481	H	-4.0157	0.4076	-5.0927
O	-1.2311	1.4647	2.0345	H	-4.4195	-1.1069	-4.2664
C	-3.0621	-0.6642	-1.9365	H	-2.7434	-0.7690	-4.7403
O	-1.8196	-1.2848	-2.3354	H	0.2093	1.4383	3.3897
C	-3.4163	0.3764	-3.0130	H	0.0316	-4.2489	-0.4894
C	-2.3809	1.4924	-3.1465	H	2.1514	-5.2637	-1.3206
C	-3.6637	-0.3189	-4.3538	H	3.0575	-4.5879	-3.5265
C	-1.4485	-2.3975	-1.6487	H	1.8541	-2.9150	-4.9038
O	-2.0432	-2.8017	-0.6743	H	-0.2331	-1.8842	-4.0576
C	-0.2189	-3.0025	-2.2150				
1-6							
C	4.3047	-0.9783	-1.6633	O	0.2491	0.1031	3.9111
C	5.3330	-1.1230	-0.7318	C	0.1270	-3.7290	-2.1404
C	5.1027	-0.9922	0.6377	C	1.2642	-4.4708	-2.4268
C	3.8049	-0.7030	1.0350	C	2.1437	-4.0441	-3.4195
C	2.7687	-0.5259	0.1106	C	1.8833	-2.8746	-4.1287
C	3.0105	-0.6673	-1.2437	C	0.7552	-2.1185	-3.8369
N	3.2927	-0.5614	2.3286	H	4.5123	-1.1047	-2.7213
C	1.8380	-0.4883	2.2877	H	6.3369	-1.3529	-1.0768
C	1.5153	-0.0695	0.8262	H	5.8954	-1.1089	1.3678
C	0.1676	-0.5757	0.2870	H	2.2121	-0.5426	-1.9715
C	-0.4207	0.6376	-0.4342	H	1.3875	-1.4852	2.4647
C	0.3645	1.8345	0.1171	H	0.3139	-1.4313	-0.3753
O	1.4745	1.3867	0.7362	H	-0.4778	-0.8867	1.1122
C	3.8107	0.0010	3.4730	H	-0.1919	0.6087	-1.4995

C	2.5987	0.3278	4.3660	H	1.5332	-0.6401	5.9868
N	1.5170	0.4505	3.3478	H	3.2905	-0.9373	5.9692
C	2.3965	-0.8371	5.3469	H	2.2297	-1.7849	4.8243
C	2.7879	1.6465	5.0966	H	2.9562	2.4580	4.3839
O	4.9842	0.1692	3.7280	H	1.8911	1.8711	5.6825
O	0.1220	2.9967	-0.0566	H	3.6472	1.5817	5.7701
N	-1.8655	0.8191	-0.2791	H	-3.5069	1.8639	3.2974
C	-2.2640	1.1634	1.0298	H	-5.9670	2.1252	3.6066
C	-3.6868	1.3275	1.2263	H	-7.4988	1.7286	1.6969
C	-4.5494	1.1029	0.1369	H	-6.5904	1.0729	-0.5256
N	-4.0766	0.7410	-1.1018	H	-3.3258	-0.2112	-3.1066
C	-2.8003	0.6228	-1.2856	H	-2.1187	1.1761	-4.6345
C	-4.1988	1.6946	2.4787	H	-3.3884	3.2440	-4.3877
C	-5.5618	1.8382	2.6414	H	-4.4024	1.8945	-3.8336
C	-6.4275	1.6138	1.5583	H	-3.6327	2.9654	-2.6576
C	-5.9350	1.2504	0.3210	H	-0.9113	3.2417	-3.9669
O	-1.4084	1.3011	1.9034	H	-0.0852	1.8111	-3.3552
C	-2.4472	0.3317	-2.7431	H	-1.0587	2.8221	-2.2609
O	-1.2857	-0.5028	-2.9720	H	-0.3552	0.7362	3.4892
C	-2.2468	1.5851	-3.6212	H	-0.5744	-4.0449	-1.3742
C	-3.4953	2.4684	-3.6229	H	1.4676	-5.3837	-1.8756
C	-1.0035	2.4016	-3.2711	H	3.0339	-4.6256	-3.6408
C	-1.3282	-1.7608	-2.4595	H	2.5644	-2.5490	-4.9095
O	-2.2268	-2.1577	-1.7514	H	0.5513	-1.2040	-4.3835
C	-0.1260	-2.5436	-2.8370				
1-7							
C	1.8129	1.0398	-4.3900	O	-3.8924	1.8864	-0.4781
C	0.9770	1.3517	-5.4626	C	2.1423	-3.7802	0.1557
C	-0.3718	1.6523	-5.2752	C	2.0818	-5.0170	-0.4716
C	-0.8494	1.6229	-3.9728	C	2.7358	-5.2207	-1.6840
C	-0.0266	1.2969	-2.8877	C	3.4486	-4.1821	-2.2798
C	1.3113	1.0010	-3.0879	C	3.5069	-2.9407	-1.6643
N	-2.1425	1.8918	-3.5076	H	2.8606	0.8198	-4.5688
C	-2.1535	1.9310	-2.0525	H	1.3844	1.3674	-6.4693
C	-0.8639	1.1787	-1.6324	H	-1.0281	1.8934	-6.1035
C	-0.2578	1.6582	-0.3010	H	1.9618	0.7285	-2.2591
C	0.1768	0.3593	0.3759	H	-2.0758	2.9731	-1.6838
C	-0.6316	-0.7392	-0.3254	H	0.5827	2.3356	-0.4725
O	-1.1642	-0.2411	-1.4564	H	-1.0200	2.1770	0.2847
C	-3.3870	1.6140	-4.0264	H	1.2190	0.1712	0.1289
C	-4.3573	1.6336	-2.8279	H	-5.6710	3.0775	-1.8852
N	-3.4255	1.3221	-1.7078	H	-5.5508	3.2269	-3.6566

C	-4.9899	3.0305	-2.7383	H	-4.2345	3.8147	-2.6207
C	-5.4143	0.5500	-2.9567	H	-4.9453	-0.4344	-3.0328
O	-3.6591	1.4238	-5.1924	H	-6.0654	0.5651	-2.0769
O	-0.7274	-1.8856	0.0144	H	-6.0186	0.7248	-3.8513
N	0.0099	0.2965	1.8266	H	-3.6203	0.6090	3.6299
C	-1.3228	0.3746	2.2731	H	-3.8631	0.7476	6.1083
C	-1.4844	0.4236	3.7128	H	-1.8507	0.6350	7.5528
C	-0.3381	0.3511	4.5271	H	0.4073	0.3691	6.5391
N	0.9219	0.1699	3.9997	H	2.7070	0.6971	1.4469
C	1.0653	0.1251	2.7172	H	3.4031	0.6500	3.8852
C	-2.7560	0.5622	4.2845	H	2.5828	-1.6567	4.4902
C	-2.8816	0.6388	5.6576	H	3.7718	-2.3496	3.3657
C	-1.7413	0.5750	6.4738	H	4.3169	-1.4516	4.7889
C	-0.4835	0.4307	5.9219	H	4.9943	0.9227	1.9605
O	-2.2395	0.4238	1.4559	H	5.1515	-0.8354	1.8380
C	2.4585	-0.1086	2.1520	H	5.7283	-0.0025	3.2849
O	2.4071	-1.3617	1.4134	H	-3.6748	1.2001	0.1728
C	3.5739	-0.1920	3.2030	H	1.6154	-3.6091	1.0869
C	3.5491	-1.4927	4.0097	H	1.5136	-5.8231	-0.0179
C	4.9375	-0.0117	2.5282	H	2.6840	-6.1904	-2.1708
C	2.9339	-1.3922	0.1619	H	3.9535	-4.3410	-3.2277
O	3.3836	-0.4079	-0.3967	H	4.0485	-2.1149	-2.1145
C	2.8590	-2.7382	-0.4412				
2-1							
C	-2.9420	-0.2458	-2.7801	C	2.1548	2.5970	-0.4704
C	-2.7086	0.9418	-2.0527	N	1.0531	1.6176	-0.3584
C	-2.9977	2.1901	-2.6222	C	1.9528	3.8758	0.3442
C	-3.5128	2.2584	-3.9013	O	4.5391	2.0666	-0.1638
C	-3.7452	1.0805	-4.6285	C	-3.0026	-1.7584	4.2064
C	-3.4659	-0.1557	-4.0802	H	-2.8113	3.0841	-2.0357
N	-2.6717	-1.4955	-2.2571	H	-3.7392	3.2226	-4.3459
C	-2.1885	-1.5353	-1.0650	H	-4.1506	1.1422	-5.6345
N	-1.9174	-0.4433	-0.2700	H	-3.6405	-1.0759	-4.6285
C	-2.1637	0.8643	-0.7078	H	-1.9633	-2.4987	-0.6153
O	-1.9146	1.8373	0.0092	H	-1.2208	0.3804	1.4703
C	-1.3825	-0.6303	1.0783	H	0.2246	-1.4655	2.2052
C	-2.4359	-1.2913	1.9678	H	-0.2153	-2.4564	0.8191
C	-0.0621	-1.4217	1.1489	H	0.9799	0.9080	1.6773
O	-3.4160	-1.8773	1.5777	H	1.9936	-3.6065	0.6339
O	-2.0976	-1.1416	3.2602	H	4.3433	-4.2538	1.1128
C	1.1056	-0.8392	0.3463	H	6.0818	-2.5266	1.4040
C	2.4274	-1.4979	0.7217	H	5.5027	-0.1018	1.2584

C	3.4223	-0.5350	0.8999	H	0.8813	-0.0866	-1.4104
N	2.8675	0.7475	0.7491	H	2.3351	2.8523	-1.5223
C	1.4107	0.6660	0.6877	H	0.1508	2.0515	-0.1759
C	2.7497	-2.8423	0.7938	H	1.8064	3.6451	1.4053
C	4.0739	-3.2041	1.0495	H	2.8312	4.5213	0.2478
C	5.0550	-2.2276	1.2148	H	1.0770	4.4249	-0.0166
C	4.7464	-0.8694	1.1429	H	-2.5817	-1.5464	5.1889
O	0.8710	-0.9951	-1.0447	H	-4.0011	-1.3248	4.1087
C	3.3690	1.8102	0.0273	H	-3.0575	-2.8357	4.0299
2-2							
C	-0.7716	-1.3823	-3.7221	C	1.7066	2.9431	0.0405
C	-1.1511	-0.0818	-3.3241	N	0.8692	1.7452	-0.1834
C	-1.2193	0.9558	-4.2647	C	0.9194	4.2401	0.2349
C	-0.9133	0.7046	-5.5872	O	3.5234	3.0954	1.6942
C	-0.5346	-0.5866	-5.9874	C	-4.6119	-1.8486	2.2902
C	-0.4636	-1.6181	-5.0722	H	-1.5186	1.9432	-3.9276
N	-0.6902	-2.4320	-2.8277	H	-0.9659	1.5044	-6.3194
C	-0.9754	-2.1738	-1.5995	H	-0.2953	-0.7763	-7.0300
N	-1.3527	-0.9456	-1.1023	H	-0.1728	-2.6220	-5.3646
C	-1.4680	0.1783	-1.9302	H	-0.9252	-2.9725	-0.8642
O	-1.7972	1.2776	-1.4761	H	-1.8935	0.2730	0.4520
C	-1.6664	-0.7908	0.3181	H	-0.8971	-0.9994	2.2969
C	-2.9391	-1.5682	0.6555	H	-0.3457	-2.2754	1.2183
C	-0.5345	-1.1969	1.2824	H	-0.2744	1.3779	1.6073
O	-3.4304	-2.4377	-0.0220	H	1.6990	-2.8409	2.5392
O	-3.4240	-1.1551	1.8394	H	3.4115	-2.7847	4.3376
C	0.8005	-0.4719	1.0874	H	4.4417	-0.6474	5.0170
C	1.7399	-0.7044	2.2645	H	3.7555	1.4963	3.9344
C	2.3262	0.4960	2.6694	H	1.5427	-0.0927	-0.6479
N	1.8060	1.5546	1.9052	H	2.4259	3.0660	-0.7790
C	0.6712	1.0958	1.1078	H	-0.0185	1.9616	-0.6313
C	2.1263	-1.8940	2.8583	H	0.3533	4.4838	-0.6699
C	3.0992	-1.8615	3.8593	H	0.2166	4.1511	1.0710
C	3.6798	-0.6534	4.2431	H	1.6069	5.0646	0.4471
C	3.3027	0.5527	3.6530	H	-4.8618	-1.3987	3.2507
O	1.4206	-0.9046	-0.1140	H	-5.4236	-1.7094	1.5718
C	2.4981	2.5821	1.2988	H	-4.4064	-2.9164	2.4017
2-3							
C	1.3701	-2.1347	0.9739	C	1.9614	2.8847	1.1373
C	0.3655	-1.6580	1.8457	N	0.5204	3.0589	0.8170
C	0.5776	-1.6376	3.2309	C	2.1791	2.1857	2.4781
C	1.7813	-2.0768	3.7463	O	3.6537	1.9651	-0.4299

C	2.7842	-2.5469	2.8844	C	-4.3846	-3.2027	0.2927
C	2.5849	-2.5821	1.5180	H	-0.2159	-1.2634	3.8706
N	1.1964	-2.1812	-0.3976	H	1.9543	-2.0570	4.8178
C	0.1051	-1.6829	-0.8640	H	3.7294	-2.8880	3.2972
N	-0.9040	-1.1227	-0.1108	H	3.3502	-2.9435	0.8386
C	-0.8808	-1.1504	1.2922	H	-0.0647	-1.6736	-1.9413
O	-1.8391	-0.7319	1.9335	H	-1.9584	-0.7162	-1.8293
C	-2.0654	-0.5041	-0.7564	H	-2.9945	1.3418	-1.2200
C	-3.3732	-1.1742	-0.3229	H	-2.4674	1.2372	0.4601
C	-2.1729	1.0181	-0.5726	H	-0.0859	1.0738	0.9369
O	-4.4297	-0.5972	-0.2487	H	-1.7110	1.3502	-3.6822
O	-3.2016	-2.4929	-0.1377	H	-0.1789	0.4297	-5.4210
C	-0.9281	1.8446	-0.9477	H	2.1783	-0.0576	-4.8677
C	-0.1909	1.3202	-2.1604	H	3.0566	0.3410	-2.5665
C	1.1451	1.0433	-1.8620	H	-1.9068	3.4860	-0.5152
N	1.4135	1.3513	-0.5246	H	2.4726	3.8531	1.1303
C	0.1790	1.8014	0.1624	H	0.4382	3.7894	0.1106
C	-0.6783	1.1110	-3.4405	H	3.2492	2.0318	2.6460
C	0.1849	0.6058	-4.4135	H	1.7709	2.7937	3.2898
C	1.5179	0.3332	-4.0990	H	1.6897	1.2056	2.5006
C	2.0237	0.5491	-2.8193	H	-4.0736	-4.2411	0.4058
O	-1.3303	3.1903	-1.2334	H	-4.7344	-2.7958	1.2451
C	2.5135	2.0283	-0.0218	H	-5.1764	-3.1116	-0.4556
2-4							
C	-3.3417	-1.7780	1.4081	C	2.0822	1.1137	2.4406
C	-2.0557	-2.2005	1.8105	N	1.1205	0.4517	1.5333
C	-1.8566	-2.7490	3.0856	C	3.2841	0.2513	2.8288
C	-2.9235	-2.8758	3.9525	O	3.0452	3.3494	2.0746
C	-4.2034	-2.4565	3.5570	C	0.5723	-3.1837	-4.4162
C	-4.4145	-1.9154	2.3043	H	-0.8570	-3.0680	3.3631
N	-3.5812	-1.2313	0.1622	H	-2.7744	-3.3007	4.9404
C	-2.5707	-1.1148	-0.6263	H	-5.0380	-2.5598	4.2448
N	-1.2766	-1.4873	-0.3358	H	-5.3975	-1.5867	1.9825
C	-0.9352	-2.0594	0.8963	H	-2.7162	-0.6900	-1.6160
O	0.2272	-2.3864	1.1504	H	0.7035	-1.6361	-0.8351
C	-0.2178	-1.3066	-1.3285	H	0.7980	0.1278	-2.5381
C	-0.4512	-2.2435	-2.5140	H	-0.9229	0.4628	-2.3930
C	-0.0384	0.1396	-1.8309	H	2.1061	0.0891	-0.3506
O	-1.4922	-2.7930	-2.7798	H	-0.7377	2.8993	-2.9035
O	0.6764	-2.3492	-3.2382	H	0.2594	5.1032	-3.4733
C	0.2436	1.1894	-0.7518	H	2.2059	5.9561	-2.2188
C	0.7119	2.5056	-1.3600	H	3.2275	4.6060	-0.3828

C	1.8191	2.9999	-0.6681	H	-0.6549	1.2034	0.9507
N	2.1977	2.0840	0.3284	H	1.5730	1.4685	3.3456
C	1.4679	0.8290	0.1678	H	1.1010	-0.5594	1.6473
C	0.1398	3.2575	-2.3719	H	3.9678	0.8270	3.4601
C	0.6933	4.5018	-2.6805	H	2.9562	-0.6318	3.3868
C	1.7935	4.9823	-1.9716	H	3.8309	-0.0825	1.9399
C	2.3793	4.2376	-0.9482	H	1.5586	-3.1550	-4.8786
O	-0.9197	1.4107	0.0310	H	0.3054	-4.2044	-4.1310
C	2.5179	2.3453	1.6444	H	-0.1879	-2.7873	-5.0943
2-5							
C	2.7202	0.0361	-0.0862	C	-0.8586	3.4001	1.0876
C	2.2074	-0.3970	1.1570	N	-1.8007	2.2781	1.3387
C	2.8369	-0.0273	2.3533	C	0.3418	3.3743	2.0324
C	3.9611	0.7741	2.3168	O	0.0082	4.0402	-1.1497
C	4.4726	1.2101	1.0848	C	0.5016	-5.3453	0.9082
C	3.8677	0.8455	-0.1024	H	2.4145	-0.3770	3.2903
N	2.1348	-0.3145	-1.2893	H	4.4494	1.0693	3.2404
C	1.0372	-0.9845	-1.2299	H	5.3559	1.8422	1.0643
N	0.4099	-1.3898	-0.0717	H	4.2518	1.1739	-1.0629
C	0.9945	-1.2005	1.1903	H	0.5254	-1.2615	-2.1524
O	0.4595	-1.6604	2.1935	H	-1.0974	-2.2380	-1.1849
C	-0.8903	-2.0641	-0.1199	H	-2.9615	-1.8300	0.2545
C	-0.8319	-3.4492	0.5319	H	-1.9500	-1.2168	1.5631
C	-2.0552	-1.2557	0.4728	H	-0.4832	0.6846	1.1220
O	-1.7660	-3.9650	1.0941	H	-3.3292	-1.1604	-2.4106
O	0.3525	-4.0398	0.3065	H	-2.5886	-0.6887	-4.7447
C	-2.2547	0.1670	-0.0834	H	-0.8801	1.0405	-5.1804
C	-1.9816	0.2883	-1.5665	H	0.1443	2.3234	-3.3016
C	-1.0182	1.2656	-1.8271	H	-3.8327	0.3699	1.0606
N	-0.5969	1.8561	-0.6319	H	-1.3797	4.3598	1.1675
C	-1.2459	1.2003	0.5283	H	-2.7007	2.5212	0.9260
C	-2.5606	-0.4174	-2.6089	H	0.0094	3.4983	3.0664
C	-2.1512	-0.1432	-3.9144	H	0.8888	2.4278	1.9563
C	-1.1867	0.8365	-4.1585	H	1.0318	4.1837	1.7758
C	-0.6039	1.5606	-3.1209	H	1.5065	-5.6731	0.6428
O	-3.6129	0.5680	0.1397	H	0.3920	-5.2670	1.9930
C	-0.4069	3.2045	-0.3751	H	-0.2505	-6.0340	0.5144
2-6							
C	-1.9784	1.7266	-0.7012	C	2.7477	0.8727	-2.2646
C	-2.0173	0.4989	-1.3993	N	2.7035	-0.4857	-1.6628
C	-2.3713	0.4624	-2.7547	C	1.7444	1.0376	-3.4042
C	-2.6741	1.6368	-3.4155	O	2.7173	2.9900	-0.9685

C	-2.6328	2.8597	-2.7280	C	-4.0167	-3.2964	1.6048
C	-2.2952	2.9090	-1.3895	H	-2.3895	-0.4989	-3.2590
N	-1.6457	1.8039	0.6391	H	-2.9426	1.6162	-4.4672
C	-1.2778	0.7114	1.2120	H	-2.8698	3.7794	-3.2553
N	-1.2029	-0.5236	0.6050	H	-2.2598	3.8466	-0.8440
C	-1.6510	-0.7285	-0.7092	H	-0.9816	0.7284	2.2616
O	-1.6678	-1.8585	-1.1863	H	-0.5237	-1.3447	2.3645
C	-0.6856	-1.6863	1.3327	H	0.9620	-3.0037	1.5185
C	-1.7271	-2.8065	1.4177	H	0.4606	-2.7567	-0.1554
C	0.6417	-2.2452	0.7968	H	0.6924	-0.5449	-1.1427
O	-1.4482	-3.9790	1.4642	H	2.0276	-1.3882	3.5116
O	-2.9673	-2.3052	1.5329	H	2.0450	0.6039	5.0106
C	1.7954	-1.2362	0.6377	H	1.9188	2.8784	4.0562
C	1.8452	-0.1930	1.7319	H	1.7522	3.2188	1.5900
C	1.7782	1.0988	1.2053	H	2.9684	-2.6845	0.0361
N	1.7046	1.0473	-0.1906	H	3.7588	1.0993	-2.6188
C	1.6561	-0.3603	-0.6556	H	3.5772	-0.6398	-1.1605
C	1.9488	-0.3847	3.1003	H	0.7215	0.8298	-3.0703
C	1.9690	0.7329	3.9355	H	1.7738	2.0656	-3.7778
C	1.9000	2.0180	3.3935	H	1.9864	0.3512	-4.2199
C	1.8056	2.2254	2.0193	H	-4.9447	-2.7298	1.6796
O	3.0404	-1.9460	0.6565	H	-4.0082	-3.9112	0.7010
C	2.4135	1.8230	-1.0953	H	-3.8774	-3.9347	2.4814
2-7							
C	-0.7785	-3.4928	1.4901	C	2.1015	2.1000	1.8031
C	0.5440	-3.0351	1.3013	N	1.3287	1.0768	1.0676
C	1.5713	-3.4703	2.1499	C	3.5820	2.1715	1.4253
C	1.2877	-4.3490	3.1767	O	1.4318	4.4566	2.0418
C	-0.0256	-4.8051	3.3686	C	-0.2676	-3.5710	-4.1711
C	-1.0476	-4.3867	2.5392	H	2.5782	-3.1039	1.9763
N	-1.8199	-3.0910	0.6741	H	2.0800	-4.6885	3.8366
C	-1.5318	-2.2596	-0.2647	H	-0.2406	-5.4958	4.1791
N	-0.2853	-1.7456	-0.5336	H	-2.0687	-4.7291	2.6737
C	0.8364	-2.1077	0.2205	H	-2.3233	-1.9134	-0.9280
O	1.9527	-1.6492	-0.0334	H	0.9611	-0.5551	-1.6260
C	-0.1039	-0.8270	-1.6610	H	-0.7006	1.0317	-2.4958
C	-0.2773	-1.5228	-3.0136	H	-2.0209	0.2017	-1.6979
C	-0.9565	0.4482	-1.6057	H	1.2972	1.6260	-1.0187
O	-0.4388	-0.9209	-4.0484	H	-3.3953	2.2289	-1.2872
O	-0.1812	-2.8578	-2.9150	H	-4.1143	4.6054	-1.2571
C	-0.7791	1.3190	-0.3610	H	-2.5862	6.3650	-0.4455
C	-1.4285	2.6863	-0.5357	H	-0.2803	5.7911	0.3236

C	-0.5740	3.6970	-0.0920	H	-0.5947	0.5929	1.4136
N	0.6507	3.1341	0.3069	H	2.0019	1.9504	2.8856
C	0.7141	1.7282	-0.0844	H	1.8921	0.2768	0.7894
C	-2.7088	3.0034	-0.9560	H	4.0875	1.2334	1.6764
C	-3.1153	4.3389	-0.9264	H	3.7036	2.3565	0.3521
C	-2.2507	5.3324	-0.4693	H	4.0672	2.9848	1.9735
C	-0.9582	5.0287	-0.0421	H	-0.1772	-4.6249	-3.9095
O	-1.3334	0.6683	0.7756	H	-1.2269	-3.3695	-4.6545
C	1.3772	3.4001	1.4479	H	0.5440	-3.2622	-4.8350
2-8							
C	-3.5934	-1.3279	-0.6383	C	1.1885	2.6046	-2.0056
C	-3.3165	0.0425	-0.4384	N	0.5268	1.5931	-1.1547
C	-4.1160	1.0214	-1.0448	C	1.1125	4.0352	-1.4689
C	-5.1781	0.6433	-1.8423	O	3.4823	2.4102	-2.8778
C	-5.4558	-0.7174	-2.0442	C	-2.2135	-1.2368	4.8862
C	-4.6779	-1.6930	-1.4521	H	-3.8805	2.0662	-0.8690
N	-2.8313	-2.3249	-0.0578	H	-5.7997	1.3981	-2.3138
C	-1.8390	-1.9457	0.6679	H	-6.2927	-1.0065	-2.6737
N	-1.4773	-0.6446	0.9258	H	-4.8781	-2.7496	-1.5983
C	-2.1949	0.4348	0.3998	H	-1.2091	-2.6982	1.1407
O	-1.8670	1.5993	0.6396	H	-0.2565	0.7286	1.8143
C	-0.3298	-0.3684	1.7945	H	1.7578	-0.6623	2.0513
C	-0.5905	-0.7630	3.2504	H	0.9673	-2.0541	1.3377
C	1.0040	-0.9583	1.3149	H	1.5855	1.5151	0.7227
O	0.2895	-0.8711	4.0707	H	2.9788	-3.0060	0.2374
O	-1.8975	-0.9249	3.5089	H	5.3453	-3.2707	-0.4671
C	1.4628	-0.5311	-0.0801	H	6.5697	-1.3798	-1.4749
C	2.9114	-0.9303	-0.3361	H	5.4619	0.8388	-1.7716
C	3.6235	0.1302	-0.8983	H	0.2803	-0.3569	-1.5910
N	2.7914	1.2595	-0.9891	H	0.7861	2.5699	-3.0258
C	1.5408	1.0290	-0.2699	H	-0.2599	1.9683	-0.6300
C	3.5229	-2.1625	-0.1789	H	1.5442	4.1007	-0.4639
C	4.8494	-2.3125	-0.5876	H	1.6671	4.7102	-2.1280
C	5.5406	-1.2433	-1.1556	H	0.0718	4.3723	-1.4231
C	4.9382	0.0033	-1.3221	H	-3.2972	-1.3460	4.9145
O	0.6207	-1.1111	-1.0679	H	-1.7191	-2.1644	5.1856
C	2.6356	2.1116	-2.0620	H	-1.8853	-0.4247	5.5401
2-9							
C	1.8576	-2.7649	1.9570	C	1.3374	0.9482	-3.0553
C	1.4317	-2.9468	0.6229	N	0.7974	0.3753	-1.8038
C	2.1433	-3.7938	-0.2380	C	0.7660	0.3333	-4.3340
C	3.2669	-4.4524	0.2209	O	1.4614	3.3545	-3.5568

C	3.6942	-4.2737	1.5458	C	-3.7771	-3.0967	2.5378
C	3.0024	-3.4434	2.4057	H	1.7881	-3.9160	-1.2563
N	1.1836	-1.9405	2.8390	H	3.8210	-5.1095	-0.4422
C	0.1475	-1.3291	2.3828	H	4.5795	-4.7949	1.8989
N	-0.3522	-1.4296	1.1058	H	3.3192	-3.2931	3.4328
C	0.2485	-2.2502	0.1449	H	-0.4100	-0.6655	3.0425
O	-0.2078	-2.3368	-0.9976	H	-1.7260	-0.9391	-0.3208
C	-1.5692	-0.6989	0.7408	H	-2.4519	1.2245	0.5697
C	-2.8100	-1.2490	1.4489	H	-1.4138	1.0869	1.9729
C	-1.4920	0.8243	0.9110	H	-1.3045	0.8476	-1.6967
O	-3.8481	-0.6370	1.5327	H	-0.9788	3.5158	2.2141
O	-2.6188	-2.4929	1.9152	H	-1.0418	5.9609	1.7832
C	-0.3553	1.5284	0.1684	H	-0.5960	6.8446	-0.4777
C	-0.5485	3.0401	0.1576	H	-0.1104	5.2954	-2.3754
C	-0.3105	3.5578	-1.1163	H	1.4317	0.8165	0.0559
N	-0.0372	2.5053	-2.0071	H	2.4324	0.8775	-3.0677
C	-0.3139	1.2182	-1.3734	H	0.5094	-0.5948	-1.9068
C	-0.8109	3.8989	1.2112	H	1.1775	0.8456	-5.2090
C	-0.8331	5.2737	0.9691	H	1.0262	-0.7282	-4.3990
C	-0.5826	5.7718	-0.3087	H	-0.3255	0.4261	-4.3600
C	-0.3160	4.9196	-1.3799	H	-3.4477	-4.0838	2.8606
O	0.8925	1.2102	0.7719	H	-4.1035	-2.4956	3.3903
C	0.9718	2.4294	-2.9434	H	-4.5942	-3.1740	1.8159
2-10							
C	-4.0505	-2.3150	0.8204	C	3.4657	0.1516	-2.7373
C	-4.0431	-0.9513	1.1903	N	2.1555	-0.1625	-2.1097
C	-5.0734	-0.4247	1.9804	C	3.3810	1.3478	-3.6819
C	-6.1025	-1.2438	2.4016	O	5.6292	0.4259	-1.5443
C	-6.1148	-2.5993	2.0380	C	-2.8895	2.0757	-2.8298
C	-5.1062	-3.1318	1.2589	H	-5.0354	0.6275	2.2451
N	-3.0543	-2.8769	0.0413	H	-6.9031	-0.8409	3.0142
C	-2.1016	-2.0954	-0.3372	H	-6.9276	-3.2374	2.3733
N	-1.9955	-0.7604	-0.0330	H	-5.1012	-4.1777	0.9687
C	-2.9544	-0.0940	0.7445	H	-1.2884	-2.4928	-0.9449
O	-2.8233	1.1004	0.9879	H	-0.3203	-0.6022	-1.2204
C	-0.8545	0.0304	-0.4984	H	0.0961	1.5075	0.7770
C	-1.3035	1.2489	-1.3088	H	-0.2557	-0.0297	1.5765
C	0.1019	0.4230	0.6473	H	1.7004	1.5924	-1.0229
O	-0.6854	2.2847	-1.3625	H	1.5186	-0.1666	3.3001
O	-2.4025	0.9769	-2.0274	H	3.5225	0.3179	4.6904
C	1.5214	-0.0568	0.3891	H	5.6441	0.9967	3.6294
C	2.5346	0.3035	1.4587	H	5.8067	1.2591	1.1523

C	3.7418	0.7098	0.8710	H	2.2419	-1.8724	0.6572
N	3.5813	0.7823	-0.5119	H	3.8484	-0.7227	-3.2752
C	2.1598	0.6044	-0.8709	H	2.1586	-1.1447	-1.8377
C	2.4462	0.1545	2.8337	H	4.3766	1.5968	-4.0614
C	3.5758	0.4172	3.6109	H	2.7230	1.1156	-4.5235
C	4.7734	0.8048	3.0092	H	2.9792	2.2262	-3.1657
C	4.8797	0.9598	1.6279	H	-3.7794	1.6954	-3.3309
O	1.4574	-1.4963	0.2395	H	-3.1380	2.9235	-2.1861
C	4.4143	0.4566	-1.5564	H	-2.1312	2.3787	-3.5567
2-11							
C	4.3791	-1.8081	0.6660	C	-2.2826	3.2109	-2.0070
C	3.3215	-2.7427	0.5964	N	-1.1975	2.2986	-1.5593
C	3.5362	-4.0837	0.9413	C	-3.0379	2.6583	-3.2129
C	4.7891	-4.4948	1.3523	O	-4.0323	4.2213	-0.5578
C	5.8428	-3.5707	1.4238	C	0.6660	-2.2787	-3.7915
C	5.6457	-2.2456	1.0869	H	2.7014	-4.7746	0.8753
N	4.2123	-0.4748	0.3338	H	4.9607	-5.5328	1.6201
C	3.0360	-0.1137	-0.0502	H	6.8255	-3.9016	1.7478
N	1.9421	-0.9376	-0.1543	H	6.4504	-1.5189	1.1365
C	2.0040	-2.3030	0.1607	H	2.8520	0.9275	-0.3162
O	1.0031	-3.0028	0.0531	H	0.8390	0.5979	-0.9689
C	0.6448	-0.4133	-0.5872	H	-1.1613	-1.0887	0.4107
C	0.1005	-1.1897	-1.7897	H	0.1303	-0.5723	1.5029
C	-0.3800	-0.3407	0.5633	H	-2.2331	0.5327	-1.0339
O	-1.0740	-1.3745	-1.9995	H	-1.1268	0.2111	3.4773
O	1.0899	-1.5409	-2.6240	H	-2.9488	0.8910	5.0269
C	-1.0024	1.0419	0.6858	H	-4.8174	2.2860	4.2223
C	-2.0327	1.1970	1.7886	H	-4.9440	2.9970	1.8324
C	-3.1125	1.9732	1.3404	H	-0.2223	2.6527	1.5129
N	-2.9586	2.2353	-0.0197	H	-1.8743	4.2000	-2.2417
C	-1.8370	1.4456	-0.5663	H	-0.5039	2.8520	-1.0579
C	-1.9615	0.8062	3.1162	H	-2.3687	2.5880	-4.0744
C	-2.9805	1.1982	3.9865	H	-3.4303	1.6579	-3.0018
C	-4.0384	1.9840	3.5283	H	-3.8782	3.3134	-3.4619
C	-4.1259	2.3859	2.1963	H	1.5790	-2.4897	-4.3478
O	0.0879	1.9714	0.9037	H	0.1738	-3.2059	-3.4862
C	-3.2250	3.3426	-0.7893	H	-0.0252	-1.6781	-4.3889
2-12							
C	-4.2349	-0.9025	1.9873	C	2.0302	0.5939	-3.8752
C	-3.3645	-0.0781	2.7353	N	1.0066	0.4016	-2.8149
C	-3.6549	0.2329	4.0703	C	2.3479	2.0688	-4.1080
C	-4.7984	-0.2700	4.6594	O	4.2236	-0.5681	-4.0162

C	-5.6659	-1.0900	3.9215	C	-1.9357	4.0650	0.0222
C	-5.3926	-1.4045	2.6047	H	-2.9647	0.8700	4.6145
N	-3.9885	-1.2341	0.6664	H	-5.0277	-0.0318	5.6935
C	-2.9159	-0.7538	0.1367	H	-6.5635	-1.4818	4.3918
N	-2.0040	0.0530	0.7714	H	-6.0536	-2.0367	2.0203
C	-2.1624	0.4505	2.1075	H	-2.6733	-0.9924	-0.8992
O	-1.3270	1.1776	2.6334	H	-0.9516	0.2783	-0.9843
C	-0.8083	0.5319	0.0747	H	1.1023	0.6108	1.1030
C	-0.7161	2.0601	0.1003	H	0.2205	-0.9077	1.3133
C	0.4862	-0.1311	0.5902	H	1.9162	1.2296	-1.0948
O	0.3245	2.6723	0.1027	H	2.0569	-2.5362	1.6539
O	-1.9315	2.6199	0.0116	H	4.3020	-3.5882	1.8328
C	1.2797	-0.7755	-0.5359	H	6.0396	-3.1193	0.1452
C	2.5826	-1.4330	-0.1217	H	5.5963	-1.5445	-1.7405
C	3.5870	-1.1606	-1.0626	H	0.9504	-2.5175	-1.3990
N	3.1020	-0.2583	-2.0081	H	1.6998	0.1300	-4.8111
C	1.7829	0.2570	-1.5905	H	0.5493	-0.4958	-2.9706
C	2.8285	-2.3103	0.9225	H	2.6558	2.5535	-3.1754
C	4.0868	-2.9081	1.0148	H	3.1613	2.1671	-4.8332
C	5.0692	-2.6387	0.0615	H	1.4633	2.5875	-4.4867
C	4.8390	-1.7590	-0.9951	H	-2.9850	4.3515	-0.0449
O	0.4094	-1.7582	-1.1494	H	-1.4913	4.4301	0.9518
C	3.2810	-0.1582	-3.3677	H	-1.3695	4.4512	-0.8295
3-1							
C	-1.8808	2.8800	1.4148	O	-0.8207	1.1959	-2.6633
C	-2.1427	1.5225	0.7064	O	1.9613	-4.0161	-0.8575
C	-0.9483	1.1325	-0.1923	H	-1.1063	2.6483	2.1635
C	0.2982	2.0336	0.0416	H	0.5417	1.9345	1.1091
C	-0.1693	3.4438	-0.2100	H	0.3549	4.0614	-0.9351
C	-1.2567	3.8722	0.4427	H	-1.2561	5.8115	-0.4701
C	-1.8403	5.2384	0.2547	H	-2.8749	5.1879	-0.1076
C	-3.0935	3.3801	2.1980	H	-1.8652	5.8006	1.1963
C	-3.3833	1.5294	-0.2261	H	-2.8166	4.2136	2.8504
N	-2.8055	1.5231	-1.5561	H	-3.4860	2.5798	2.8350
C	-1.4749	1.2772	-1.6420	H	-3.9091	3.7285	1.5570
C	-4.3527	0.3426	0.0101	H	-3.9598	2.4543	-0.0894
C	-3.6767	-1.0020	-0.0094	H	-3.3554	1.6192	-2.3967
C	-3.3259	-1.6111	-1.2171	H	-4.8409	0.5049	0.9795
C	-2.6004	-2.7971	-1.2324	H	-5.1405	0.3877	-0.7538
C	-2.2209	-3.4018	-0.0384	H	-3.6093	-1.1449	-2.1571
C	-2.5912	-2.8224	1.1714	H	-2.3195	-3.2442	-2.1814
C	-3.3138	-1.6335	1.1835	H	-1.6342	-4.3154	-0.0503

C	1.4931	1.5721	-0.7404	H	-2.3106	-3.2932	2.1097
C	2.5503	0.9939	-0.1689	H	-3.5958	-1.1866	2.1348
C	3.7573	0.5217	-0.9198	H	1.4450	1.6741	-1.8227
O	-0.5764	-0.2672	-0.1209	H	2.5720	0.8737	0.9183
C	0.0358	-0.6754	0.9865	H	4.6076	1.1766	-0.6766
O	0.7432	-1.7815	0.6466	H	3.5780	0.6110	-1.9971
C	1.5876	-2.2952	1.6084	H	1.3122	-2.0959	2.6386
C	2.6520	-2.9818	1.2116	H	3.2920	-3.3990	1.9839
C	2.9928	-3.2663	-0.2398	H	4.4106	-1.0371	0.4585
C	3.1829	-1.9462	-1.0476	H	4.5884	-4.2557	-1.3376
C	4.2212	-0.9200	-0.6175	H	4.0648	-5.0773	0.1445
O	2.5282	-1.7878	-2.0577	H	5.0948	-3.6352	0.2625
C	4.2796	-4.1007	-0.3004	H	5.3347	-1.0991	-2.4746
C	5.5247	-1.1825	-1.3995	H	6.2733	-0.4328	-1.1240
H	-2.2703	0.7639	1.4825	H	5.9457	-2.1700	-1.2021
O	0.0000	-0.1774	2.0868	H	1.6005	-3.4383	-1.5539
3-2							
C	-1.1758	2.7339	0.1132	O	0.7083	0.1735	-3.1236
C	-1.4810	1.3216	-0.4584	O	1.8259	-4.9548	0.1865
C	-0.1797	0.5919	-0.8510	H	-0.7261	2.5329	1.0989
C	1.0945	1.3154	-0.3370	H	0.9831	1.3915	0.7535
C	1.0228	2.7064	-0.9159	H	1.8568	3.0853	-1.5013
C	-0.0969	3.4134	-0.7199	H	0.5786	5.1208	-1.8265
C	-0.3012	4.7920	-1.2672	H	-1.1674	4.8332	-1.9394
C	-2.4376	3.5527	0.3793	H	-0.4916	5.5186	-0.4678
C	-2.3542	1.3241	-1.7411	H	-2.2007	4.4448	0.9670
N	-1.4190	0.9735	-2.7940	H	-3.1622	2.9586	0.9466
C	-0.1982	0.5357	-2.3996	H	-2.9348	3.8868	-0.5364
C	-3.5331	0.3285	-1.6714	H	-2.7620	2.3263	-1.9298
C	-4.5553	0.7550	-0.6533	H	-1.6651	0.9702	-3.7726
C	-4.6063	0.1649	0.6113	H	-3.9971	0.2666	-2.6649
C	-5.5017	0.6215	1.5744	H	-3.1356	-0.6661	-1.4342
C	-6.3612	1.6763	1.2848	H	-3.9394	-0.6627	0.8429
C	-6.3269	2.2656	0.0235	H	-5.5278	0.1501	2.5527
C	-5.4316	1.8065	-0.9357	H	-7.0604	2.0330	2.0353
C	2.3527	0.5461	-0.6139	H	-7.0025	3.0826	-0.2137
C	3.0738	-0.0317	0.3475	H	-5.4124	2.2692	-1.9207
C	4.3467	-0.7812	0.1006	H	2.6457	0.4359	-1.6560
O	-0.1662	-0.8183	-0.4797	H	2.7543	0.0643	1.3893
C	0.0396	-1.0921	0.8099	H	5.1856	-0.2048	0.5189
O	0.5817	-2.3332	0.8647	H	4.5188	-0.8684	-0.9779
C	1.0106	-2.7537	2.1108	H	0.4809	-2.3322	2.9582

C	2.0126	-3.6197	2.1884	H	2.3037	-3.9437	3.1839
C	2.7306	-4.2303	0.9982	H	4.2711	-2.1319	1.8098
C	3.4005	-3.1311	0.1223	H	4.3747	-5.6126	0.6676
C	4.4336	-2.1903	0.7246	H	3.2862	-6.0387	2.0016
O	3.1227	-3.0829	-1.0587	H	4.4749	-4.7363	2.2240
C	3.7990	-5.2092	1.5049	H	6.5963	-2.0753	0.8424
C	5.8395	-2.7588	0.4443	H	5.9994	-3.7403	0.8950
H	-1.9927	0.7596	0.3271	H	5.9926	-2.8489	-0.6361
O	-0.2042	-0.3910	1.7626	H	1.8102	-4.4887	-0.6688
4-1							
C	1.4009	3.1973	1.0784	C	-0.2949	-1.0000	-1.2170
C	1.8762	2.0580	1.7262	H	1.9828	4.1169	1.0867
C	1.1244	0.8907	1.6948	H	1.5087	0.0024	2.1886
C	-0.1047	0.8474	1.0365	H	-0.2037	4.0473	-0.0813
C	-0.5928	2.0026	0.3975	H	3.4956	2.8872	2.3299
C	0.1796	3.1648	0.4210	H	-0.2548	-1.2518	1.4403
O	3.0740	2.0236	2.3966	H	-3.8716	-0.9942	3.7586
C	-0.8833	-0.4547	1.0123	H	-2.6177	-0.7813	5.0288
O	-1.3511	-0.8144	-0.2779	H	-3.2141	0.6308	4.0888
C	-2.1289	-0.2883	1.8746	H	-2.2934	1.2599	-0.1206
O	-3.1676	0.1944	1.4820	H	-0.7674	-1.3150	-2.1494
O	-1.8983	-0.6855	3.1339	H	0.2648	-0.0703	-1.3856
C	-2.9811	-0.4369	4.0609	H	0.4018	-1.7822	-0.8790
O	-1.7724	2.0659	-0.2736				
4-2							
C	1.3298	3.4118	1.3605	C	-1.5866	-1.7930	-0.8756
C	1.8455	2.2429	1.9182	H	1.8332	4.3636	1.5182
C	1.1948	1.0355	1.7008	H	1.6048	0.1294	2.1374
C	0.0290	0.9816	0.9380	H	-0.2361	4.2687	0.1537
C	-0.4945	2.1605	0.3784	H	3.3454	3.1121	2.7347
C	0.1737	3.3658	0.5950	H	-0.0191	-1.1564	1.1037
O	2.9877	2.2193	2.6809	H	-3.4904	-1.2367	3.6235
C	-0.6812	-0.3411	0.7718	H	-2.2377	-0.8361	4.8488
O	-1.0430	-0.5046	-0.5880	H	-3.0545	0.4673	3.9191
C	-1.9283	-0.3319	1.6701	H	-1.9419	1.3060	-0.5370
O	-3.0388	-0.0309	1.3001	H	-2.5249	-1.9662	-0.3332
O	-1.6005	-0.6639	2.9299	H	-1.7827	-1.8145	-1.9493
C	-2.6760	-0.5580	3.8907	H	-0.8616	-2.5810	-0.6243
O	-1.6332	2.2098	-0.3621				
4-3							
C	1.2050	3.5921	1.2661	C	-1.4507	-1.8289	-0.7983
C	1.7797	2.4694	1.8608	H	1.6711	4.5623	1.4061

C	1.1948	1.2233	1.6794	H	1.6362	0.3399	2.1381
C	0.0330	1.0810	0.9149	H	-0.3981	4.3238	0.0318
C	-0.5468	2.2123	0.3207	H	3.2111	1.8116	2.9546
C	0.0584	3.4588	0.5025	H	0.0945	-1.0513	1.1494
O	2.9205	2.6594	2.6014	H	-2.1188	-0.7116	4.8969
C	-0.6085	-0.2805	0.7947	H	-2.9965	0.5237	3.9309
O	-0.9660	-0.5076	-0.5568	H	-3.3581	-1.2066	3.6928
C	-1.8501	-0.3040	1.6997	H	-1.9443	1.2582	-0.5736
O	-2.9763	-0.0732	1.3280	H	-2.3797	-2.0257	-0.2482
O	-1.4986	-0.5733	2.9691	H	-1.6459	-1.8966	-1.8702
C	-2.5734	-0.4843	3.9328	H	-0.6901	-2.5737	-0.5214
O	-1.6842	2.1815	-0.4231				
4-4							
C	1.3152	3.3927	1.4661	C	-1.8097	-1.6915	-0.8397
C	1.9213	2.1990	1.8542	H	1.8000	4.3459	1.6676
C	1.2923	0.9912	1.5823	H	1.7696	0.0666	1.8936
C	0.0587	0.9589	0.9320	H	-0.3876	4.2913	0.4984
C	-0.5536	2.1631	0.5411	H	3.4588	3.0493	2.6186
C	0.0919	3.3694	0.8117	H	0.1361	-1.1819	0.8066
O	3.1335	2.1509	2.4966	H	-4.6198	0.3352	2.4503
C	-0.6146	-0.3804	0.7384	H	-3.8962	-1.1931	3.0635
O	-1.2607	-0.4119	-0.5280	H	-3.2957	0.3746	3.6650
C	-1.5719	-0.6069	1.9200	H	-2.0205	1.3343	-0.3465
O	-1.2424	-1.1547	2.9444	H	-2.5926	-1.9803	-0.1248
O	-2.7842	-0.0726	1.6887	H	-2.2450	-1.6122	-1.8376
C	-3.7079	-0.1491	2.7986	H	-1.0211	-2.4572	-0.8456
O	-1.7588	2.2334	-0.0880				
